# Immunoinformatic insights for design and clinical prospects of pan-RAS cancer vaccine

**DOI:** 10.3389/fmed.2026.1727976

**Published:** 2026-03-05

**Authors:** Ruby Srivastava, Thakur Rochak Kumar Rana

**Affiliations:** Department of Chemistry, Indian Institute of Technology Bombay, Mumbai, India

**Keywords:** immunity, Kirsten rat sarcoma viral oncogene homolog, mRNA vaccine, pancreatic cancer, toll like receptors

## Abstract

**Introduction:**

KRAS mutations, particularly at codon 12, occur in over 90% of pancreatic ductal adenocarcinoma (PDAC) cases and drive tumor initiation, progression, and therapeutic resistance. Targeting oncogenic KRAS variants through immunotherapy offers a promising strategy for PDAC treatment. This study evaluated Seq ID No. 70 of a pan-RAS mRNA cancer vaccine (WO 2022/081764 A1; PCT/US 2021/054859), incorporating KRAS variants G12D, G13D, L19F, A59T, G60D, Q61H, K117N, and A146T, for its suitability in PDAC.

**Methods:**

Immunoinformatic analyses were performed to predict cytotoxic T lymphocyte (CTL), helper T lymphocyte (HTL), and B-cell epitopes, which were assessed for antigenicity, allergenicity, and toxicity. Physicochemical profiling, secondary structure prediction, and 3D structural modeling with refinement were conducted. Molecular docking and 500 ns molecular dynamics simulations evaluated interactions with Toll-like receptors TLR7/8 and the agonist resiquimod (R848). Solvent-accessible surface area (SASA) analysis examined epitope exposure. Codon optimization assessed expression efficiency, and immune simulations predicted immunogenicity.

**Results:**

Multiple epitopes demonstrated favorable antigenicity with non-allergenic and non-toxic profiles. The construct was predicted to be stable, hydrophilic, and structurally refined. Docking and MD simulations showed stable interactions with TLR7/8; no direct R848–vaccine binding was observed. SASA analysis indicated strong solvent exposure, supporting immunogenic potential. Codon optimization yielded favorable parameters (CAI = 0.956; GC = 61.7%) and a molecular weight of 21,973.98 Da. Immune simulations predicted robust cellular and humoral responses.

**Discussion:**

The pan-RAS mRNA vaccine demonstrates structural stability and strong immunogenic potential for PDAC. Synergistic immune activation with R848 may enhance anti-tumor efficacy, warranting experimental validation and toxicity assessment for clinical translation.

## 1 Introduction

Pancreatic ductal adenocarcinoma (PDAC) continues to be one of the deadliest cancers, with only minimal improvement in the 5-year survival rate over the past three decades, despite extensive clinical trials. PDAC presents several specific challenges, such as its tendency for early metastatic spread and a strong preference for liver metastasis (1). It is also highly resistant to anti-tumor immune responses and immunotherapies because of its dense, immunosuppressive tumor microenvironment (TME), low immunogenicity, and overall systemic immune suppression. PDAC is further characterized by a low mutational burden, impaired antigen presentation, and increased expression of immune checkpoint molecules, all of which limit immune recognition. Together, these factors classify PDAC as an “immune cold” tumor, displaying very limited cytotoxic T-cell activity (1). Importantly, most patients with PDAC do not develop clear or specific symptoms at early stages, making timely diagnosis and treatment difficult. The main therapeutic approaches for PDAC are chemotherapy and surgery; however, only about 20% of patients are eligible for surgical resection at the time of diagnosis (2). Among those who undergo surgery, nearly 80% experience local recurrence within 2 years of tumor removal (3). For patients with locally advanced or metastatic pancreatic cancer, current treatment relies on systemic chemotherapy regimens, including gemcitabine combined with nab-paclitaxel, or folfirinox (a combination of fluorouracil, oxaliplatin, irinotecan, and leucovorin). While these chemotherapy regimens can extend survival, patients still face significant challenges due to long-term toxicities, reduced quality of life, and the eventual development of drug resistance (4). Additional treatment approaches, such as radiation therapy, targeted therapies, and immunotherapies, have also been evaluated in this patient population, but so far their clinical effectiveness has remained limited (5, 6). Overall, PDAC remains an extremely difficult cancer to treat, with poor long-term survival outcomes, highlighting the urgent need for new therapeutic approaches. One promising strategy to address PDAC’s resistance to immunotherapy is the identification of immunogenic tumor-associated and tumor-specific antigens for the design of personalized cancer vaccines.

The mRNA-based vaccine platform has emerged as an attractive option for efficient antigen presentation. When delivered through lipid nanoparticles (LNPs), mRNA cancer vaccines can enter cells effectively and provide a controlled release of their payload, thereby enhancing vaccine efficacy (7). mRNA vaccines offer multiple notable advantages as they are non-infectious, generally well tolerated, rapidly degradable, and do not integrate into the host genome (8, 9). These vaccines work by delivering synthetic mRNA sequences encoding tumor antigens (TAs), which then stimulate the immune system to recognize and eliminate cancer cells (9). A unique feature of mRNA vaccines is their ability to encode full-length TAs, enabling antigen-presenting cells (APCs) to display multiple epitopes via both class I and class II human leukocyte antigens (HLAs) (10). This feature reduces the dependence on specific HLA types and increases the likelihood of generating a broader T-cell response with stronger antitumor activity (10). Furthermore, mRNA vaccines only need to reach the cytoplasm to be translated into the target TAs (9). The transient expression of TA-encoding mRNA ensures that antigen exposure is time-limited, reducing the risk of adverse effects that might occur with prolonged antigen presence (11). Additionally, because mRNA does not integrate into the host genome, there is no concern of insertional mutagenesis (9). Nevertheless, mRNA is naturally unstable and highly prone to degradation, necessitating the use of stabilizing agents such as lipid nanoparticles, polymers, or peptides to improve its stability and effectiveness (12). Neoantigens are a specialized group of tumor-specific antigens (TSAs) that are exclusively expressed in tumor cells and arise from non-synonymous mutations (13). During cancer progression, numerous genetic alterations occur, including single-nucleotide substitutions, reading frameshifts, alternative splicing events, gene fusions, and other mutagenic mechanisms (14). These mutations can cause changes in amino acid sequences, resulting in the formation of abnormal proteins expressed only in malignant cells. Research has confirmed that somatic mutations in tumors can generate a wide array of neoantigens that the immune system recognizes as foreign (15, 16). Cancer vaccines utilize these neoantigens to activate the immune system (17). When introduced, they enable immune recognition of these components as foreign, prompting a targeted immune response that involves both antibody production and T-cell activation (18). Importantly, the immune response generated by such vaccines establishes immunological memory, allowing the immune system to mount a quicker and more effective defence when tumors are encountered again in the future (18).

Among the most clinically relevant neoantigen sources is the KRAS gene, which is mutated in approximately 30% of lung cancers, 50% of colon cancers, and up to 90% of pancreatic cancers (19). Mutations in KRAS, including G12D, G12V, and G12R, impair guanosine triphosphate (GTP) hydrolysis, leading to constant KRAS activation and subsequent stimulation of the RAF–MEK–ERK and PI3K-AKT–mTOR pathways that drive tumor growth and survival (20). Specifically, in pancreatic cancer, KRAS mutations such as G12D (33–52%), G12V (23–36%), and G12R (11–20%) are the most frequently observed (21). Given the central role of KRAS-driven oncogenesis, strategies that not only target KRAS mutations but also enhance immune recognition are of great importance. In this context, innate immune sensors such as Toll-like receptors (TLRs) provide additional opportunities to boost antitumor immunity. Notably, TLR7 and TLR8 are broadly expressed on many cell types, including immune cells, and are known to promote the activation and maturation of dendritic cells (DCs). They also have the ability to reprogram tumor-infiltrating macrophages and myeloid-derived suppressor cells (MDSCs) from an immunosuppressive state to an immuno stimulatory phenotype (22, 23).

**Figure 1:**
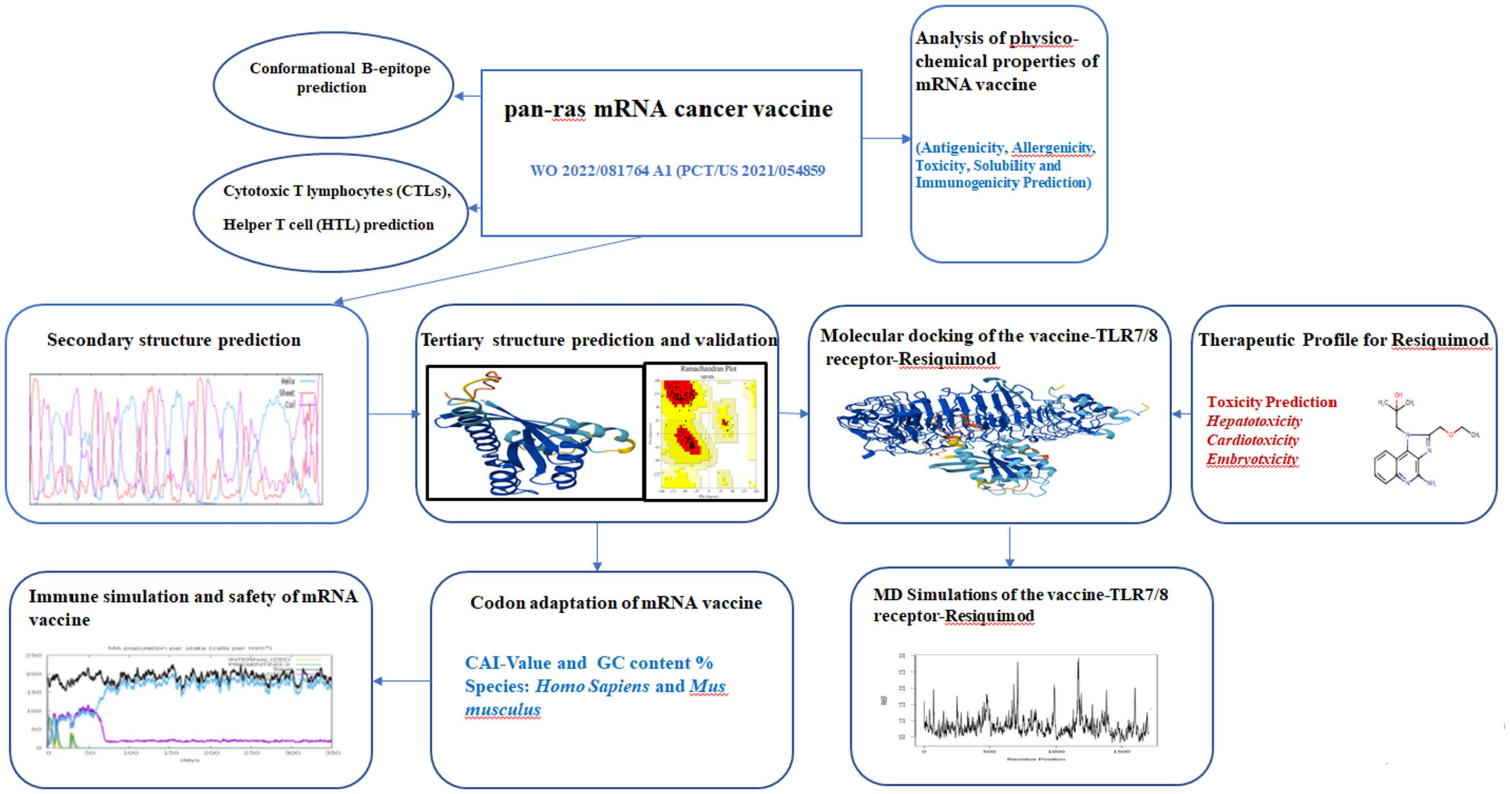
The flowchart for the immunoinformatics studies for pan-RAS mRNA vaccine.

Building on the importance of targeting neoantigens and enhancing immune recognition, this immunoinformatic studies focuses on evaluating the WO 2022/081764 A1 (PCT/US 2021/054859), a designed pan-RAS mRNA cancer vaccine against PDAC, by applying the reverse vaccinology approach. Reverse vaccinology, which relies on genomic information from pathogenic microorganisms to identify potential antigens, uses a variety of computational algorithms to predict T-cell and B-cell epitopes (24). Unlike conventional vaccinology, this method does not require pathogen culturing or protein extraction, processes that are both costly and time-consuming. For the current investigation, SEQ ID NO: 70, carrying specific mutations such as G12D, G13D, L19F, A59T, G60D, Q61H, K117N, and A146T, were selected for detailed study. A pan-RAS mRNA cancer vaccine could be used in multiple solid tumors (pancreatic, colorectal, lung, melanoma, head & neck, bladder) and some blood cancers, wherever KRAS, NRAS, or HRAS mutations drive tumor growth. The objective of this study is to compare bioinformatic predictions with experimental data, validate the predictive models, and enhance confidence in their application for future vaccine development. While tools such as immune simulation analysis serve as valuable complementary resources, they are not standalone predictive engines and currently lack extensive experimental validation. Nonetheless, these analyses play a crucial role in expediting vaccine design, epitope selection, and hypothesis generation. Therefore, it is essential to validate the predicted characteristics of final mRNA vaccine candidates through wet-lab experiments and clinical trials. The workflow of the entire studies is given in Figure 1.

## 2 Computational details

### 2.1 Details of pan-RAS mRNA cancer vaccine

The entire details of pan-RAS mRNA cancer vaccine is given in the patent file number WO 2022/081764 A1 (PCT/US 2021/054859) (25). The FASTA sequence of SEQ ID NO 70 with following mutations G12D, G13D, L19F, A59T, G60D, Q61H, K117N and A146T is selected for the study. See Supplementary material. As described earlier (26), NetMHCpan v4.0 (27)^1^ is used to calculate the binding affinity of both mutated and matching wild-type peptides with HLA (MHC-I) alleles. For further analysis, a peptide was classified as a neoantigen only if the mutated form showed strong binding affinity (IC50 < 500 nM) while the corresponding wild-type peptide exhibited weak binding (IC50 > 500 nM), and such peptides were included in the analysis.

### 2.2 Cytotoxic T lymphocyte (CTL) epitope prediction

Cytotoxic T lymphocytes generally play a role in the containment of infections (28), and the prevalence of cytotoxic T cells usually correlates with the rate of pathogen clearance. Since these peptides are CD8 + T cell epitopes, they first need to be processed by the proteasome in antigen-presenting cells (APCs), and then transported by the Transporter Associated with Antigen Processing (TAP) system to HLA molecules. The resulting HLA–peptide complex is recognized by the T cell receptor (TCR), which activates CD8 + T cells. NetCTL v1.2^2^ (20) is an immunoinformatics tool used to predict cytotoxic T lymphocyte (CTL) epitopes, which combines three factors: proteasomal cleavage, TAP transport efficiency, and MHC-I binding affinity. In NetCTL v1.2, MHC-I supertypes (A1, A2, A3, A24, A26, B7, B8, B27, B39, B44, B59, and B62) are included for predicting 9-mer CTL epitopes on neoantigens. The tool provides a combined score, and a cut-off value of 0.75 was used in this study. At this threshold, the sensitivity and specificity for CTL epitope prediction are 80 and 97%, respectively (29).

#### 2.2.1 Neoantigen antigenicity, toxicity and allergenicity prediction

The antigenicity of neoantigens is predicted by VaxiJen v2.0^3^ (30) with a threshold score of 0.5. To ensure safety, AllerTOP v2.0^4^ (31) ToxinPred (32)^5^ are used to predict the toxicity and allergenicity. For AllerTOP v2.0, the k-nearest neighbors (kNN) algorithm is used, which employs E-descriptors and auto- and cross-covariance (ACC) transformation of amino acid sequences to classify peptides as allergens or non-allergens. In ToxinPred, a Support Vector Machine (SVM) based method with default settings was applied. Immunogenecity is predicted with Immune Epitope Database (IEDB) server, which is a manually curated database of experimentally characterized immune epitopes. (33).^6^

### 2.3 Helper T lymphocyte (HTL) epitopes prediction

HTL epitopes are important for proper immune function because they release cytokines such as IFN-*γ*, IL-2, IL-4, and IL-10. Among them, IL-2 and IL-4 play key roles in activating CD8 + T cells. When HTL cells bind to the MHC II–peptide complex, they become activated and can either support CD8 + T cell–mediated immune responses (which kill tumor cells) or B cell–mediated antibody responses. Since CD8 + T cell–mediated immunity is central in cancer treatment, we focus our studies in identifying effective HTL epitopes. NetMHCpan v4.0 tool is used to predict the HTLs for the given vaccine sequence. The antigenicity, allergenicity and toxicity of the HTLs are again predicted by VaxiJen v2.0, AllerTOP v2.0 and ToxinPred respectively, though evaluating antigenicity is a prime step in vaccine design.

### 2.4 The physicochemical properties of the mRNA pan-RAS vaccine sequence

The Expasy ProtParam server^7^ is used to calculate several physicochemical properties of the multi-epitope vaccine, including amino acid composition, theoretical pI, molecular weight, instability index, half-life, aliphatic index, and the grand average of hydropathicity (GRAVY) (34).

### 2.5 Secondary structure prediction

The secondary structure of pan-RAS mRNA cancer vaccine is predicted by Prabi server.^8^ (35) Prabi server provides all the regions which produce (coiled, helical, and extended stranded) structures.

### 2.6 Tertiary structure prediction

The Alphafold3^9^ (36) server is used to predict the 3D structure of the pan-RAS mRNA cancer vaccine. Alphafold3 can generate highly accurate predictions of the structures of biomolecular complexes. To check the accuracy of the model, we performed validation using the ProSA-web server^10^ (37) and the SAVES v6.0 server.^11^ ProSA-web calculates a Z-score that reflects the overall quality of the protein model if the Z-score falls outside the normal range for native proteins; it suggests possible structural errors (37, 38). The PROCHECK tool within SAVES v6.0 further evaluates stereochemical quality by analysing residue geometry and the overall 3D structure of the protein (39, 40).

### 2.7 Prediction of B-cell epitopes

B lymphocytes are key players in the immune system because they produce antibodies, which help provide long-term immunity (41). B-cell epitopes are predicted to stimulate strong production of antigen-specific antibodies in the serum. Under natural conditions, antibody generation begins when immunogenic epitopes interact with B-cell receptors, triggering B-cell differentiation into plasma cells and memory cells. Plasma cells are primarily responsible for antibody secretion during the initial immune response, while memory cells contribute to rapid antibody production upon subsequent exposures. Therefore, in epitope-based vaccine design, a key goal is to identify and predict B-cell epitopes within the target protein. To identify B-cell epitopes, we predicted linear epitopes using the BCPREDS server.^12^ This tool uses a subsequence kernel-based SVM classifier with about 74.57% accuracy to predict linear B-cell epitopes (41–44). For discontinuous (conformational) epitopes, we used the ElliPro server.^13^ ElliPro applies residue clustering and Thornton’s method to predict these epitopes. Each predicted epitope is given a score called the PI (protrusion index) value, which shows how much the epitope stands out on the protein’s surface (45).

### 2.8 Solvent-accessible surface area (SASA) prediction

The solvent-accessible surface area (SASA) of the mRNA pan-RAS cancer vaccine is predicted using GETAREA, a tool from the FANTOM program (46).^14^ This tool measures different SASA parameters, such as the total surface area, solvation energy, and contributions from backbone and side chains, to check which residues are buried or exposed. Residues with high SASA values (>20) are more exposed to the solvent, which often suggests they may be more immunogenic.

### 2.9 Molecular docking

In pancreatic cancer, Toll Like Receptor (TLR7/8) exhibits a complex dual role. TLR7 is frequently expressed in the tumor stroma and on pancreatic cancer cells, where it contributes to chronic inflammation and therapy resistance. Thus, therapeutic targeting of TLR7/8 requires careful contextual consideration to maximize its anti-tumor benefits while minimizing pro-tumor risks. The TLR7/8 agonists such as R848 can enhance anti-tumor immune responses and reduce cachexia in murine models, particularly when combined with radiotherapy. On the other hand, they may also drive pro-proliferative and pro-inflammatory effects, potentially promoting tumor progression in immunocompetent hosts. Strategies may include combination with modalities like Stereotactic Body Radiotherapy (SBRT) or the concurrent inhibition of tumor-promoting macrophages and exosomal microRNAs (22, 47).

The docking of TLR7/8 receptors with the mRNA panras cancer vaccine is carried out using Alphafold3 sever and then HDOCK server (45)^15^ is used for the docking of (TLR7/8-pan-RAS mRNA cancer vaccine-Resiquimod) complex. The AlphaFold3 model,^16^ with its advanced diffusion-based architecture can accurately predict the joint structure of complexes involving proteins, amino acids, and nucleic acids. The mRNA vaccine sequence docked with the relevant TLRs using the AlphaFold3 server was evaluated based on their pTM and ipTM scores. Subsequently, the docked (TLRs–mRNA vaccine complex) and resiquimod subjected to additional docking using the HDOCK server, employed a hybrid approach combining template-based modeling with free docking. This strategy allows cases affected by misleading templates to be corrected through the free docking protocol. The top-ranked docking model (TLR7/8-pan-RAS mRNA cancer vaccine-resiquimod) was then selected as an input for molecular dynamics (MD) simulations.

The TLR7 (5GMH) Organism(s): (*Macaca mulatta*), and TLR8 (5AWB) is taken from the protein data bank^17^ and then after removing the water molecules, ligands and ions, the FASTA sequence is again modelled with Alphafold3 server. TLR7 is a single-stranded RNA (ssRNA) sensor in innate immunity and also responds to guanosine and chemical ligands, such as imidazoquinoline compounds. However, TLR7 activation mechanism by these ligands remains largely unknown. TLR8, an intracellular immune receptor found in endosomes that recognizes ssRNA from viruses and certain bacteria, as well as synthetic compounds activates the immune system by promoting the production of cytokines and activating immune cells like monocytes and myeloid dendritic cells. TLR8 receptors play a key role in innate and adaptive immunity against viral infections and influence immune responses in bacterial infections and autoimmunity. In this study, TLR7/8 were considered as innate immune sensors involved in endosomal recognition of mRNA-derived RNA fragments, rather than as classical binding receptors for intact vaccine constructs. Consequently, docking of large protein or peptide constructs to the ectodomains of TLR7/8 does not recapitulate their established ligand recognition or activation mechanisms. Therefore, the presented docking results should be interpreted as an exploratory structural interaction analysis that provides qualitative insights into potential spatial compatibility or contact regions, rather than as mechanistic evidence of TLR7/8 activation.

### 2.10 Molecular dynamics (MD) simulations

All-atom classical MD simulations were performed for the (TLR7–pan-RAS mRNA cancer vaccine–R848) and (TLR8–pan-RAS mRNA cancer vaccine–R848) complexes using the AMBER20 (48) software package. The AMBER ff14SB force field was applied to parameterize the TLR7 and TLR8 protein structures, while the OL3 RNA force field was employed for the pan-RAS mRNA cancer vaccine to accurately describe RNA conformational dynamics. The small-molecule agonist R848 was parameterized using the General AMBER Force Field (GAFF), with ligand atom types and partial atomic charges assigned via the Antechamber module (49) using the AM1-BCC charge model. Each system was solvated in an explicit TIP3P water model within a octahedral periodic box, maintaining a minimum distance of 10 Å between the protein and the box boundary. System neutrality was achieved by adding 12 Cl^−^ ions to the TLR7 complex and 15 Na^+^ ions to the TLR8 complex, corresponding to the net charge of each system. Protonation states of ionizable residues were assigned to reflect physiological pH (7.4), with histidine protonation states manually inspected and assigned as appropriate.

After setup and parameterization of both systems, energy minimization was carried out in two stages to remove unfavorable steric interactions. Initially, 5,000 steps of minimization were performed on solvent molecules using steepest descent method while restraining solute heavy atoms using a harmonic potential. This was followed by 5,000 steps of unrestrained minimization of the entire system using conjugate gradient algorithms. The minimized systems were then gradually heated from 10 K to 310 K under the NVT ensemble over 50 ps, applying weak positional restraints of 4 kcal mol^−1^ Å^−2^ to complexes. Subsequently, density equilibration was conducted under the NPT ensemble at 310 K and 1 atm for 1 ns using the Langevin thermostat (50) and Berendsen barostat with a collision frequency of 2 ps^−1^, followed by an additional 3 ns of unrestrained NPT equilibration to ensure proper stabilization. Production MD simulations were then performed for 500 ns under the NPT ensemble with a 2 fs time step. Long-range electrostatic interactions were treated using the Particle Mesh Ewald (PME) method with a real-space cutoff of 10 Å, while all covalent bonds involving hydrogen atoms were constrained using the SHAKE algorithm. Periodic boundary conditions were applied in all spatial directions throughout the simulations. Trajectory analyses, including assessments of structural stability, conformational dynamics, and intermolecular interactions, were performed using the CPPTRAJ module of AMBER20. All MD simulations were performed using the GPU code of PMEMD engine. MD production run has two replicas each system to ensure reproducibility and consistency. The open-source PyMOL software (51) was used for molecular visualization and preparation of publication-quality figures. ONIOM calculations were performed using Gaussian 16, Revision B.01 (52) with the AMBER force field. The optimization has been performed at B3LYP/6-311G*//AmberFF using Grimme Dispersion correction D3. The electronic embedding scheme was used while boundary was treated by employing hydrogen link atoms. Atomistic-level diagrams were generated using Chemcraft software (53).

#### 2.10.1 Normal mode analysis

Results from AMBER MD simulations of (TLR7/8-resiquimod-pan-RAS mRNA cancer vaccine) complexes are used to carried out the normal mode analysis using iMoDs server^18^ (54). MD simulations are used to estimate the stability and movement of the (TLR7/8-resiquimod-pan-RAS mRNA cancer vaccine) docked complexes. The iMoDs server evaluates protein stability and normal mode analysis (NMA) with interior coordinates. Various parameters are evaluated and plotted to illustrate the stability of protein. Such as: deformability, eigenvalue, covariance matrix, B factor and elastic network model.

### 2.11 Codon optimization

Codon optimization for mRNA vaccine makes use of these preferred codons to improve protein production, so fewer copies of mRNA are needed. We used the Java Codon Adaptation Tool^19^ (55) to optimize the codons in our vaccine sequence.

### 2.12 Immune simulation of mRNA cancer vaccine

To test how the vaccine might trigger an immune response, we used the C-ImmSim server^20^ (56), which has an agent-based algorithm incorporating a position-specific scoring matrix. The machine learning (ML) techniques is employed to estimate antigens and foreign particles affecting immune activity. Simulations are conducted using default parameters, except for the time steps, which were set at 1, 84, and 168 (with time step 1 representing the injection at time = 0, and each subsequent time step corresponding to 8 h). Accordingly, three injections are anticipated at 4-week intervals, consistent with the typical dosing schedule of most commercial vaccines. The Simpson’s diversity index (D) is calculated based on the resulting plot. This tool predicts how a mammalian immune system could react to a vaccine, including both antibody (humoral) and T-cell (cellular) responses (57–60). A three-dose vaccination schedule with 4 weeks between each dose is simulated. Most settings were kept at their default values: the number of adjuvants and antigen injections are set to 100 and 1,000, respectively (61). The simulation volume and steps were adjusted to 50 and 1,000, and the random seed was fixed at 12345 to ensure reproducibility. No lipopolysaccharides (LPS) were included in the setup.

### 2.13 Vaccines’ safety profile

The safety profile of vaccine with human proteins is performed with pBLAST^21^ (62) against human proteome (taxid: 9606) which is downloaded from the UniProt database. The similarity of vaccine with humans is estimated to avoid any autoimmune response (*Homo Sapiens*).

### 2.14 Toxicity prediction for resiquimod (R848)

It has been observed that the TLR7/8 agonist R848 triggers anti-tumor activity and reduces cachexia in mouse models of PDAC. *In vivo*, tumors from two out of three tested cell lines responded to R848, leading to smaller tumor size, enhanced immune complexity, greater CD8 + T-cell infiltration and activity, and reduced Treg frequency. Mice treated with R848 also exhibited improvements in both behavioral and molecular features of cachexia, resulting in nearly twice the survival time. Knockout studies demonstrated that stromal but not tumor cell TLR7 is essential for R848’s effects. Analysis of patient samples further showed that TLR7 is consistently expressed in stromal cells throughout all stages of pancreatic neoplasia, whereas epithelial expression is relatively rare. Together, these findings suggest that immune-stimulating strategies such as R848 may hold promise for treating PDAC and cancer-associated cachexia. In combination therapies it is vital to predict the toxicity of the drug used in combination therapies for the cancer patients.

So the SMILES notation of resiquimod^22^ is taken from the drug bank and cardiotoxicity of drug is predicted with *cardioToxCSM* web server (61)^23^ *cardioToxCSM*, which can predict six types of cardiac toxicity outcomes, including arrhythmia, cardiac failure, heart block, hERG toxicity, hypertension, and myocardial infarction, efficiently and accurately. Drug-induced liver injury (DILI) is a challenging endpoint in predictive toxicology because of the complex reactive metabolites that cause liver damage and the wide range of mechanisms involved in the development of the disease. The HepatoToxicity Portal^24^ (HTP) (63) is used to predict the liver toxicity of the drug. Embryotoxicity refers to the adverse and harmful effects of chemical, physical, or biological agents on an embryo’s development, leading to structural malformations, functional impairments, growth retardation, or even embryonic death. These effects can manifest in anatomical, physiological, or functional changes that are not always immediately apparent. Understanding embryotoxicity is crucial for identifying hazardous substances and implementing safety measures in various fields, including agriculture, pharmacology, and environmental protection. The embryoTox webserver^25^ (64) was trained and validated using *in vitro* bioactivity data of over 700 small molecules with teratogenicity effects. This simple and integrated platform provides a practical resource for optimising screening libraries to determine effective and safe molecules to use during pregnancy (65–70).

## 3 Results

### 3.1 The pan-RAS mRNA cancer vaccine

The FASTA sequence of SEQ ID NO 70 with following mutations G12D, G13D, L19F, A59T, G60D, Q61H, K117N and A146T is given in the Supplementary material.

### 3.2 Cytotoxic T lymphocyte (CTL) epitope prediction

Total 40 CTL epitopes are predicted, out of which only CVKIKKCII is toxin. One epitope VEDAFYTLY is immunogen while 39 epitopes are non-immunogen. 11 epitopes are non-allergenic whereas 29 are allergenic in nature. See Table 1.

**Table 1:** The predicted CTL epitope with their toxicity, allergenicity and immunogenicity for pan-RAS mRNA cancer vaccine.

S. N.	CTL epitope	MHC class I supertypes	SVM score	Toxicity	Allergenicity	Immunogenicity
1	MTEYKLVVV	A1	−0.81	Non-toxin	Non-allergen	Non-immunogen
2	IQNHFVDEY	A1, B62	−0.63	Non-toxin	Allergen	Non-immunogen
3	FVDEYDPTI	A1, A2	−0.58	Non-toxin	Allergen	Non-immunogen
4	KSFEDIHHY	A1, A3, B58, A26, B62	−1.01	Non-toxin	Allergen	Non-immunogen
5	QAQDLARSY	A1, B62	−1.25	Non-toxin	Allergen	Non-immunogen
6	VEDAFYTLY	A1, B44	−1.18	Non-toxin	Allergen	Immunogen
7	IQLIQNHFV	A2	−1.22	Non-toxin	Non-allergen	Non-immunogen
8	FVDEYDPTI	A1, A2	−0.58	Non-toxin	Allergen	Non-immunogen
9	VIDGETCLL	A2, B39	−0.72	Non-toxin	Allergen	Non-immunogen
10	CLLDILDTT	A2	−0.89	Non-toxin	Allergen	Non-immunogen
11	CVFAINNTK	A3	−1.17	Non-toxin	Allergen	Non-immunogen
12	KSFEDIHHY	A1, A3, B58, A26, B62	−1.01	Non-toxin	Allergen	Non-immunogen
13	TLYREIRQY	A3, A26, B62	−0.51	Non-toxin	Allergen	Non-immunogen
14	EIRQYFIKK	A3	−0.9	Non-toxin	Non-allergen	Non-immunogen
15	KSAFTIQLI	A24	−1.5	Non-toxin	Allergen	Non-immunogen
16	QYMRTGEGF	A24	−1.42	Non-toxin	Allergen	Non-immunogen
17	AFYTLYREI	A24	−1.35	Non-toxin	Non-allergen	Non-immunogen
18	LYREIRQYF	A24, B8	−0.4	Non-toxin	Allergen	Non-immunogen
19	FTIQLIQNH	A26	−1.19	Non-toxin	Non-allergen	Non-immunogen
20	VVIDGETCL	A26, B62	−0.62	Non-toxin	Allergen	Non-immunogen
21	FAINNTKSF	A26, B7, B8, B58, B62	−1.42	Non-toxin	Allergen	Non-immunogen
22	KSFEDIHHY	A1, A3, A26, B58, B62	−1.01	Non-toxin	Allergen	Non-immunogen
23	TLYREIRQY	A26	−0.39	Non-toxin	Allergen	Non-immunogen
24	FAINNTKSF	B7, B8, B58, A26, B62	−1.42	Non-toxin	Allergen	Non-immunogen
25	LARSYGIPF	B7, B8, B62	−1.11	Non-toxin	Allergen	Non-immunogen
26	RVEDAFYTL	B7, B39	−1.4	Non-toxin	Allergen	Non-immunogen
27	YSAMRDQYM	B8	−1.29	Non-toxin	Allergen	Non-immunogen
28	CVKIKKCII	B8	0.02	Toxin	Allergen	Non-immunogen
29	YREQIKRVK	B27	−0.87	Non-toxin	Non-allergen	Non-immunogen
30	TRQRVEDAF	B27	−0.79	Non-toxin	Allergen	Non-immunogen
31	VIDGETCLL	B39	−0.84	Non-toxin	Allergen	Non-immunogen
32	DSEDVPMVL	B39	−0.69	Non-toxin	Non-allergen	Non-immunogen
33	IEDSYRKQV	B44	−0.88	Non-toxin	Allergen	Non-immunogen
34	GETCLLDIL	B44	−1.07	Non-toxin	Allergen	Non-immunogen
35	SEDVPMVLV	B44	−0.96	Non-toxin	Non-allergen	Non-immunogen
36	REIRQYFIK	B44	−0.62	Non-toxin	Non-allergen	Non-immunogen
37	KSAFTIQL	B58 Supercharge	−1.47	Non-toxin	Allergen	Non-immunogen
38	TIQLIQNHF	B62	−1.19	Non-toxin	Non-allergen	Non-immunogen
39	YMRTGEGFL	B62	−1.39	Non-toxin	Non-allergen	Non-immunogen
40	KSFEDIHHY	B62	−0.97	Non-toxin	Allergen	Non-immunogen

### 3.3 Helper T lymphocyte (HTL) epitopes prediction

Total 133 HTL epitopes are predicted and all are non-toxic. 81 HTL epitopes are non-allergenic and 69 HTL epitopes are non-antigens. See Table 2.

**Table 2:** The predicted HTL epitopes, antigenicity, SVM Score, toxicity and allergenicity prediction for pan-RAS mRNA cancer vaccine.

Peptides	Antigenicity	SVM score	Toxicity	Allergenicity
ADDVGKSAFTIQLIQ	Probable non-antigen	−1.52	Non-toxin	Non-allergen
AFTIQLIQNHFVDEY	Probable non-antigen	−1.11	Non-toxin	Non-allergen
AFYTLYREIRQYFIK	Probable non-antigen	−0.95	Non-toxin	Non-allergen
AINNTKSFEDIHHYR	Probable antigen	−1.35	Non-toxin	Non-allergen
AMRDQYMRTGEGFLC	Probable non-antigen	−1.46	Non-toxin	Non-allergen
AQDLARSYGIPFIET	Probable non-antigen	−1.28	Non-toxin	Allergen
ARSYGIPFIETSTKT	Probable non-antigen	−1.35	Non-toxin	Allergen
CDLPSRTVDTKQAQD	Probable antigen	−0.91	Non-toxin	Non-allergen
CLLDILDTTDHEEYS	Probable antigen	−0.81	Non-toxin	Allergen
CVFAINNTKSFEDIH	Probable non-antigen	−1.25	Non-toxin	Non-allergen
DAFYTLYREIRQYFI	Probable antigen	−0.82	Non-toxin	Non-allergen
DDVGKSAFTIQLIQN	Probable non-antigen	−1.6	Non-toxin	Non-allergen
DEYDPTIEDSYRKQV	Probable antigen	−0.57	Non-toxin	Non-allergen
DHEEYSAMRDQYMRT	Probable antigen	−1.37	Non-toxin	Allergen
DILDTTDHEEYSAMR	Probable antigen	−1.13	Non-toxin	Non-allergen
DLARSYGIPFIETST	Probable non-antigen	−1.28	Non-toxin	Allergen
DLPSRTVDTKQAQDL	Probable antigen	−1.06	Non-toxin	Non-allergen
DPTIEDSYRKQVVID	Probable non-antigen	−0.73	Non-toxin	Allergen
DQYMRTGEGFLCVFA	Probable non-antigen	−1.04	Non-toxin	Non-allergen
DSEDVPMVLVGNNCD	Probable antigen	−0.49	Non-toxin	Allergen
DTKQAQDLARSYGIP	Probable non-antigen	−1.27	Non-toxin	Non-allergen
DTTDHEEYSAMRDQY	Probable antigen	−0.87	Non-toxin	Non-allergen
DVGKSAFTIQLIQNH	Probable non-antigen	−1.44	Non-toxin	Non-allergen
DVPMVLVGNNCDLPS	Probable antigen	−0.64	Non-toxin	Non-allergen
EDAFYTLYREIRQYF	Probable antigen	−0.79	Non-toxin	Non-allergen
EDVPMVLVGNNCDLP	Probable antigen	−0.52	Non-toxin	Allergen
EEKTPGCVKIKKCII	Probable non-antigen	0.12	Non-toxin	Allergen
EEYSAMRDQYMRTGE	Probable antigen	−1.39	Non-toxin	Allergen
EGFLCVFAINNTKSF	Probable non-antigen	−1.08	Non-toxin	Non-allergen
EIRQYFIKKISKEEK	Probable antigen	−1.11	Non-toxin	Non-allergen
EKTPGCVKIKKCIIM	Probable antigen	0.09	Non-toxin	Allergen
EYDPTIEDSYRKQVV	Probable non-antigen	−0.65	Non-toxin	Non-allergen
EYKLVVVGADDVGKS	Probable antigen	−0.74	Non-toxin	Allergen
EYSAMRDQYMRTGEG	Probable antigen	−1.51	Non-toxin	Allergen
FAINNTKSFEDIHHY	Probable antigen	−1.41	Non-toxin	Non-allergen
FIKKISKEEKTPGCV	Probable non-antigen	−0.71	Non-toxin	Allergen
FLCVFAINNTKSFED	Probable antigen	−1.06	Non-toxin	Non-allergen
FTIQLIQNHFVDEYD	Probable non-antigen	−0.99	Non-toxin	Non-allergen
FVDEYDPTIEDSYRK	Probable non-antigen	−0.6	Non-toxin	Non-allergen
FYTLYREIRQYFIKK	Probable antigen	−0.88	Non-toxin	Non-allergen
GADDVGKSAFTIQLI	Probable non-antigen	−1.46	Non-toxin	Allergen
GEGFLCVFAINNTKS	Probable non-antigen	−1.15	Non-toxin	Non-allergen
GFLCVFAINNTKSFE	Probable non-antigen	−1.13	Non-toxin	Non-allergen
GKSAFTIQLIQNHFV	Probable non-antigen	−1.5	Non-toxin	Allergen
GNNCDLPSRTVDTKQ	Probable non-antigen	−0.59	Non-toxin	Non-allergen
HEEYSAMRDQYMRTG	Probable antigen	−1.36	Non-toxin	Allergen
HFVDEYDPTIEDSYR	Probable antigen	−0.71	Non-toxin	Non-allergen
IEDSYRKQVVIDGET	Probable antigen	−0.92	Non-toxin	Non-allergen
IKKISKEEKTPGCVK	Probable antigen	−0.61	Non-toxin	Allergen
ILDTTDHEEYSAMRD	Probable antigen	−1.18	Non-toxin	Non-allergen
INNTKSFEDIHHYRE	Probable antigen	−1.07	Non-toxin	Allergen
IQLIQNHFVDEYDPT	Probable non-antigen	−0.93	Non-toxin	Non-allergen
IQNHFVDEYDPTIED	Probable non-antigen	−0.61	Non-toxin	Non-allergen
IRQYFIKKISKEEKT	Probable antigen	−1.03	Non-toxin	Non-allergen
ISKEEKTPGCVKIKK	Probable antigen	−0.61	Non-toxin	Non-allergen
KDSEDVPMVLVGNNC	Probable non-antigen	−0.77	Non-toxin	Allergen
KEEKTPGCVKIKKCI	Probable antigen	0.1	Non-toxin	Allergen
KISKEEKTPGCVKIK	Probable non-antigen	−0.74	Non-toxin	Non-allergen
KKISKEEKTPGCVKI	Probable antigen	−0.71	Non-toxin	Non-allergen
KLVVVGADDVGKSAF	Probable antigen	−1.19	Non-toxin	Allergen
KQAQDLARSYGIPFI	Probable antigen	−1.21	Non-toxin	Non-allergen
KRVKDSEDVPMVLVG	Probable non-antigen	−1.18	Non-toxin	Allergen
KSAFTIQLIQNHFVD	Probable antigen	−1.52	Non-toxin	Non-allergen
LARSYGIPFIETSTK	Probable non-antigen	−1.55	Non-toxin	Non-allergen
LCVFAINNTKSFEDI	Probable non-antigen	−1.15	Non-toxin	Non-allergen
LDILDTTDHEEYSAM	Probable antigen	−0.96	Non-toxin	Non-allergen
LDTTDHEEYSAMRDQ	Probable antigen	−1.06	Non-toxin	Non-allergen
LIQNHFVDEYDPTIE	Probable non-antigen	−0.64	Non-toxin	Non-allergen
LLDILDTTDHEEYSA	Probable antigen	−1.13	Non-toxin	Allergen
LPSRTVDTKQAQDLA	Probable non-antigen	−1.29	Non-toxin	Non-allergen
LVGNNCDLPSRTVDT	Probable antigen	−0.49	Non-toxin	Allergen
LVVVGADDVGKSAFT	Probable non-antigen	−1.24	Non-toxin	Non-allergen
LYREIRQYFIKKISK	Probable non-antigen	−0.82	Non-toxin	Non-allergen
MRDQYMRTGEGFLCV	Probable non-antigen	−1.34	Non-toxin	Non-allergen
MRTGEGFLCVFAINN	Probable non-antigen	−1.1	Non-toxin	Allergen
MTEYKLVVVGADDVG	Probable antigen	−0.94	Non-toxin	Allergen
MVLVGNNCDLPSRTV	Probable antigen	−0.72	Non-toxin	Allergen
NCDLPSRTVDTKQAQ	Probable antigen	−0.46	Non-toxin	Non-allergen
NHFVDEYDPTIEDSY	Probable antigen	−0.56	Non-toxin	Non-allergen
NNCDLPSRTVDTKQA	Probable antigen	−0.52	Non-toxin	Non-allergen
NNTKSFEDIHHYREQ	Probable non-antigen	−1.21	Non-toxin	Allergen
NTKSFEDIHHYREQI	Probable non-antigen	−1.22	Non-toxin	Non-allergen
PMVLVGNNCDLPSRT	Probable antigen	−0.71	Non-toxin	Non-allergen
PSRTVDTKQAQDLAR	Probable antigen	−1.25	Non-toxin	Non-allergen
PTIEDSYRKQVVIDG	Probable antigen	−0.79	Non-toxin	Allergen
QAQDLARSYGIPFIE	Probable non-antigen	−1.27	Non-toxin	Allergen
QDLARSYGIPFIETS	Probable non-antigen	−1.46	Non-toxin	Allergen
QLIQNHFVDEYDPTI	Probable non-antigen	−0.79	Non-toxin	Non-allergen
QNHFVDEYDPTIEDS	Probable antigen	−0.5	Non-toxin	Non-allergen
QYFIKKISKEEKTPG	Probable antigen	−1.14	Non-toxin	Non-allergen
QYMRTGEGFLCVFAI	Probable non-antigen	−1.17	Non-toxin	Allergen
RDQYMRTGEGFLCVF	Probable non-antigen	−1.02	Non-toxin	Allergen
REIRQYFIKKISKEE	Probable antigen	−0.67	Non-toxin	Non-allergen
RQYFIKKISKEEKTP	Probable antigen	−1.08	Non-toxin	Non-allergen
RSYGIPFIETSTKTR	Probable non-antigen	−1.11	Non-toxin	Non-allergen
RTGEGFLCVFAINNT	Probable non-antigen	−0.74	Non-toxin	Non-allergen
RTVDTKQAQDLARSY	Probable antigen	−1.22	Non-toxin	Non-allergen
RVEDAFYTLYREIRQ	Probable antigen	−0.95	Non-toxin	Allergen
RVKDSEDVPMVLVGN	Probable antigen	−1.22	Non-toxin	Allergen
SAFTIQLIQNHFVDE	Probable non-antigen	−1.37	Non-toxin	Non-allergen
SAMRDQYMRTGEGFL	Probable non-antigen	−1.6	Non-toxin	Non-allergen
SEDVPMVLVGNNCDL	Probable antigen	−0.41	Non-toxin	Allergen
SKEEKTPGCVKIKKC	Probable non-antigen	−0.19	Non-toxin	Allergen
SRTVDTKQAQDLARS	Probable antigen	−1.11	Non-toxin	Non-allergen
SYGIPFIETSTKTRQ	Probable antigen	−1.14	Non-toxin	Non-allergen
TDHEEYSAMRDQYMR	Probable antigen	−1.35	Non-toxin	Allergen
TEYKLVVVGADDVGK	Probable antigen	−0.68	Non-toxin	Allergen
TGEGFLCVFAINNTK	Probable non-antigen	−1.04	Non-toxin	Allergen
TIEDSYRKQVVIDGE	Probable antigen	−0.81	Non-toxin	Allergen
TIQLIQNHFVDEYDP	Probable non-antigen	−0.82	Non-toxin	Allergen
TKQAQDLARSYGIPF	Probable antigen	−1.47	Non-toxin	Non-allergen
TLYREIRQYFIKKIS	Probable non-antigen	−0.84	Non-toxin	Non-allergen
TTDHEEYSAMRDQYM	Probable antigen	−0.98	Non-toxin	Non-allergen
TVDTKQAQDLARSYG	Probable antigen	−1.15	Non-toxin	Non-allergen
VDEYDPTIEDSYRKQ	Probable antigen	−0.45	Non-toxin	Non-allergen
VDTKQAQDLARSYGI	Probable antigen	−1.28	Non-toxin	Non-allergen
VEDAFYTLYREIRQY	Probable antigen	−0.82	Non-toxin	Non-allergen
VFAINNTKSFEDIHH	Probable antigen	−1.42	Non-toxin	Non-allergen
VGADDVGKSAFTIQL	Probable non-antigen	−1.48	Non-toxin	Non-allergen
VGKSAFTIQLIQNHF	Probable non-antigen	−1.55	Non-toxin	Non-allergen
VGNNCDLPSRTVDTK	Probable antigen	−0.83	Non-toxin	Allergen
VKDSEDVPMVLVGNN	Probable antigen	−1.07	Non-toxin	Non-allergen
VLVGNNCDLPSRTVD	Probable antigen	−0.73	Non-toxin	Non-allergen
VPMVLVGNNCDLPSR	Probable antigen	−0.73	Non-toxin	Allergen
VVGADDVGKSAFTIQ	Probable non-antigen	−1.31	Non-toxin	Allergen
VVVGADDVGKSAFTI	Probable non-antigen	−1.17	Non-toxin	Non-allergen
YDPTIEDSYRKQVVI	Probable non-antigen	−0.7	Non-toxin	Non-allergen
YFIKKISKEEKTPGC	Probable antigen	−0.89	Non-toxin	Allergen
YKLVVVGADDVGKSA	Probable antigen	−0.91	Non-toxin	Allergen
YMRTGEGFLCVFAIN	Probable non-antigen	−1.18	Non-toxin	Allergen
YREIRQYFIKKISKE	Probable non-antigen	−0.88	Non-toxin	Allergen
YSAMRDQYMRTGEGF	Probable antigen	−1.69	Non-toxin	Allergen
YTLYREIRQYFIKKI	Probable non-antigen	−0.8	Non-toxin	Non-allergen

The predicted HTL epitope of pan-RAS mRNA cancer vaccine (WO 2022/081764 A1) (PCT/US 2021/054859) with the corresponding Alleles as predicted by NetMHCpan v4.0 tools is given in Supplementary Table 2.

### 3.4 Physicochemical properties of the mRNA pan-RAS vaccine sequence

The FASTA sequence with the above-mentioned mutations (mutations G12D, G13D, L19F, A59T, G60D, Q61H, K117N and A146T) is used to predict the biophysical and physicochemical properties of the pan-RAS mRNA cancer vaccine. Results showed that the total number of amino acids in pan-RAS mRNA cancer vaccine: 189, Molecular weight: 21,973.98 Da, and theoretical pI: 5.31. The molecular weight of pan-RAS mRNA cancer vaccine is lower than the standard MW (100 kDa). The formula predicted for the vaccine is C_970_H_1531_N_259_O_302_S_10_. Total number of negatively charged residues (Asp+Glu)-32; Total number of positively charged residues (Arg + Lys)-26 and the Total number of atoms is 3,072. The instability index (II) is computed to be 35.05, below the standard threshold of 40, indicating the stable nature of the protein. See Table 1. The Aliphatic index: 80.37 indicate the thermostable nature of the vaccine. The Grand average of hydropathicity (GRAVY) is (−0.496). The negative value GRAVY indicates the hydrophilic nature of the vaccine. The antigenicity of the vaccine, as predicted by the Vaxijen v2.0 server is 0.897175. As the value is > 0.4, pan-RAS mRNA cancer vaccine is probably an antigen. The solubility score of pan-RAS mRNA cancer vaccine is predicted by the SOLpro server, which is 0.733657. The solubility score above 0.5 (green color) indicates the soluble expression of the vaccine in *Escherichia coli*. AllerTOP v. 2.0 results indicated the non-allergenicity of the vaccine. (See Table 3).

**Table 3:** The physiochemical properties (Number of amino acids, MW, chemical formula, Theoretical pI, Total number of negatively charged residues (Asp+Glu), Total number of positively charged residues (Arg + Lys), Total number of atoms, Instability Index (II), Aliphatic index (AI), Grand Average of hydropathicity (GRAVY)) antigenicity, solubility and allergenecity of pan-RAS mRNA cancer vaccine.

Features	Assessment	Remarks
Number of amino acids	189	–
Molecular weight (MW)	21,973.98 Da	Low
Chemical formula	C_970_H_1531_N_259_O_302_S_10_	–
Theoretical pI	5.31	–
Total number of negatively charged residues (Asp + Glu)	32	–
Total number of positively charged residues (Arg + Lys)	26	–
Total number of atoms	3,072	–
Instability Index (II)	35.05	Protein as stable
Aliphatic index (AI)	80.37	Thermostable
Grand average of hydropathicity (GRAVY)	−0.496	Hydrophilic
Antigenicity	0.897175	Probable antigen
Solubility	0.733657 (probability)	Insoluble
Allergenecity	-	Probable non-allergen

Protparam server of Expasy online tools-physicochemical properties. Vaxijen v2.0 server-antigenicity, SOLpro server-solubility and AllerTOP v. 2.0 - allergenicity prediction.

### 3.5 Secondary structure prediction

**Figure 2:**
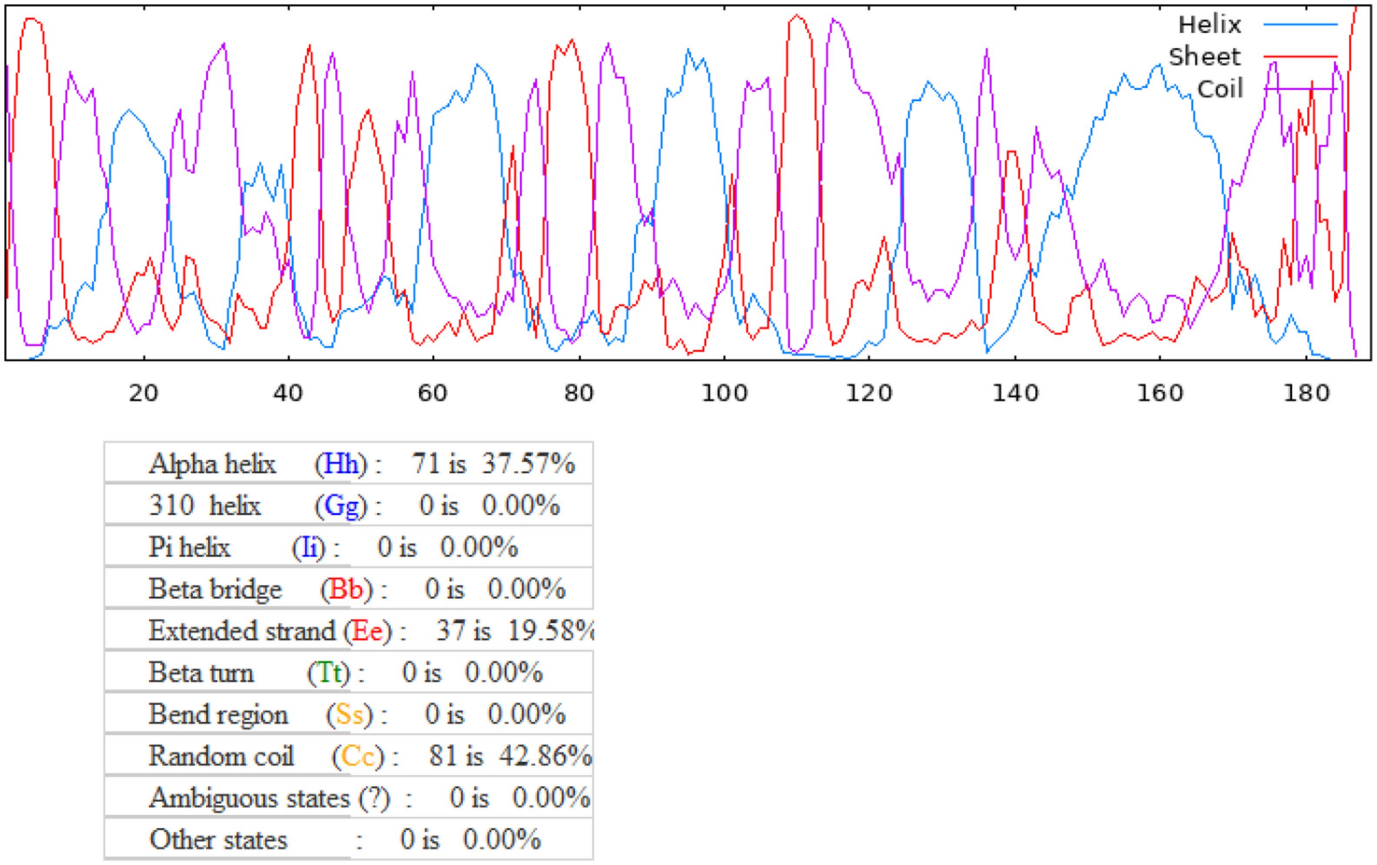
Secondary structure for pan-RAS mRNA cancer vaccine as predicted with the PRABI server (https://npsa-prabi.Ibcp.fr/cgi-bin/npsa_automat.pl.?page=/npsa/npsa_gor4.html).

The 2D structure of pan-RAS mRNA cancer vaccine is predicted by prabi server^26^ with all the coiled, helical, and extended stranded regions. Results indicate Alpha helix-71; 310 helix-0; Pi helix-0; Beta bridge-0, Extended strand-37; Beta turn-0; Beta region-0; Bend region-0; Random coil-81; Ambiguous state-0 and other states-0. Overall, the secondary structure of pan-RAS mRNA cancer vaccine has high globular conformability, stability and flexibility. See Figure 2.

### 3.6 Tertiary structure prediction

**Figure 3:**
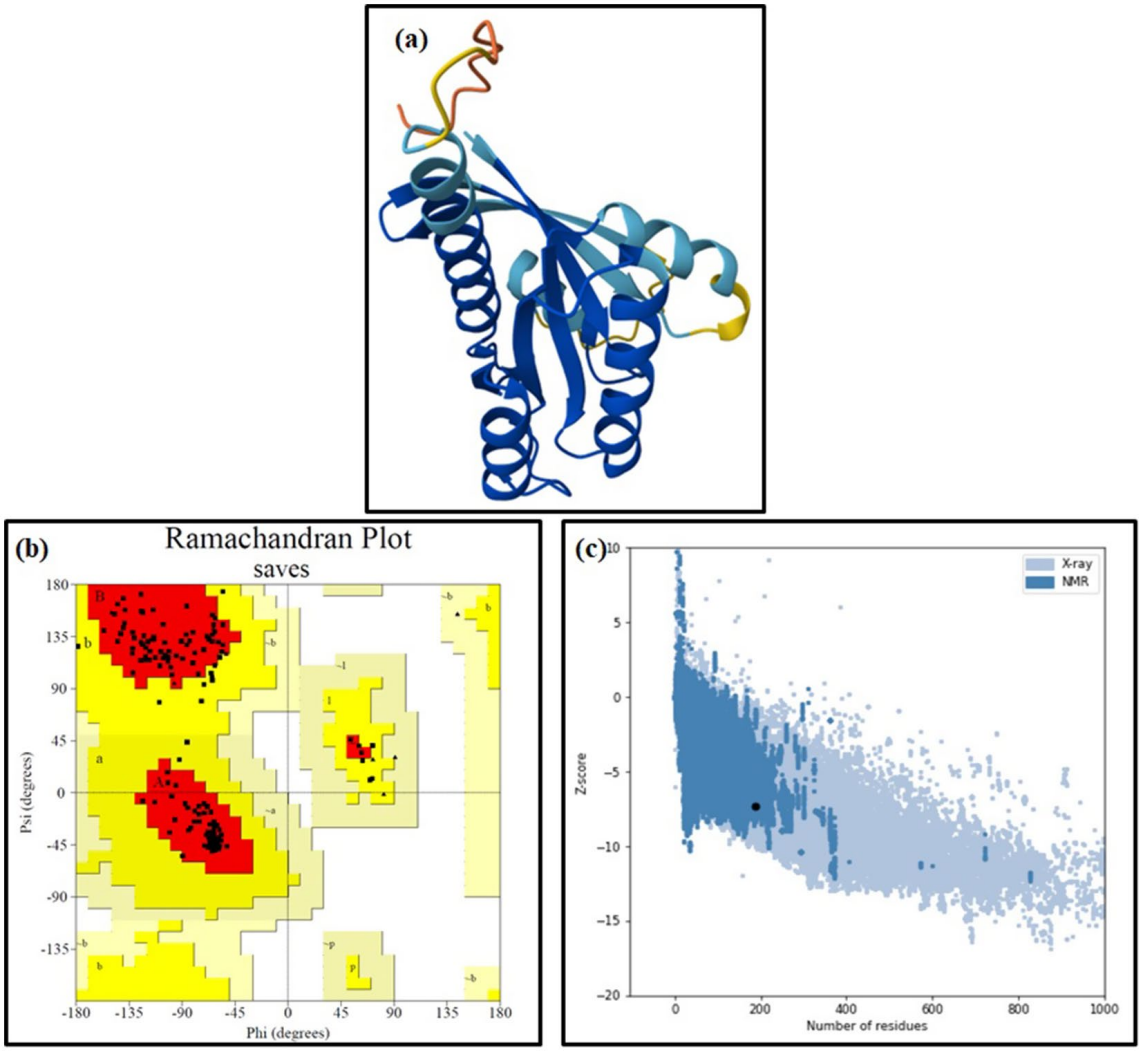
The **(a)** model structure (Alphafold3), **(b)** *Z*-score plot (ProsA webserver), and **(c)** Ramachandran plot (ProCheck server) for pan-RAS mRNA cancer vaccine.

The refined 3D structure of pan-RAS mRNA cancer vaccine as predicted by Alphafold3 server is given in Figure 3a. The pTM = 0.85 score showed stable structure of pan-RAS mRNA cancer vaccine. Ramachandran plots from the PROCHECK server are used for quality assessment of the 3D structure of the vaccine. Ramachandran plots analyse the conformational flexibility of proteins and the protein regions that causes structural disturbances. It can predict the structural abnormalities and conformations of protein loops. The Ramachandran plot predicts 91.4% core, 8. 6% allowed, 0.0% general, and 0.0% disallowed regions for the pan-RAS vaccine structure. All Ramachandrans: 89 labelled residues (out of 3,387) and Chi1-chi2 plots: 20 labelled residues (out of 1983) with better side-chain params. See Figure 3b. A Z-score of −7.32 indicates good quality of the pan-RAS mRNA cancer vaccine. The local quality shows the plotted graph of the residue scores. Negative values describe an absence of erroneous parts in the vaccine. See Figure 3c.

### 3.7 Prediction of B-cell epitopes

The Ellipro server is used to predict and analyse the immune response of conformational B-cell epitopes. The default parameters are – (minimum score- 0.5) and (maximum distance- 6 Å). The server predicted six new conformational B-cell epitopes with peptides IQNHFVDEYDPTIEDSYR, DHEEYSAM, FE, HHYREQIKRVKD, LPSRTVDTKQ and YFIKKISKEEKTPGCVKIKK with 18, 8, 2, 12, 10, 20 length. All the B-cell epitopes are non-toxin, non-immunogen with one peptide (HHYREQIKRVKD) as non-allergen. See Table 4.

**Table 4:** Continuous B Cell epitopes with peptides (start-end), length, SVM score, toxicity, Allegenicity and Immunogenicity for pan-RAS mRNA cancer vaccine as predicted with ElliPro server.

No.	Start	End	Peptide	Length	SVM Score	Toxicity	Allergenicity	Immunogenicity
1	24	41	IQNHFVDEYDPTIEDSYR	18	−0.73	Non-toxin	Allergen	Non-immunogen
2	60	67	DHEEYSAM	8	−0.57	Non-toxin	Allergen	Non-immunogen
3	90	91	FE	2	−0.8	Non-toxin	Allergen	–
4	94	105	HHYREQIKRVKD	12	−0.96	Non-toxin	Non-allergen	Non-immunogen
5	120	129	LPSRTVDTKQ	10	−1.02	Non-toxin	Allergen	Non-immunogen
6	166	185	YFIKKISKEEKTPGCVKIKK	20	−0.85	Non-toxin	Allergen	Non-immunogen

### 3.8 Solvent-accessible surface area (SASA) prediction

Solvent Accessible Surface Area (SASA) analysis using GETAREA showed that the number of surface atoms is 911, with 630 as buried atoms and number of atoms with ASP = 0. Total area/energy is 10,911.86 Å^2^ per kcal/mol. (Supplementary Table 1)

### 3.9 Molecular docking

**Figure 4:**
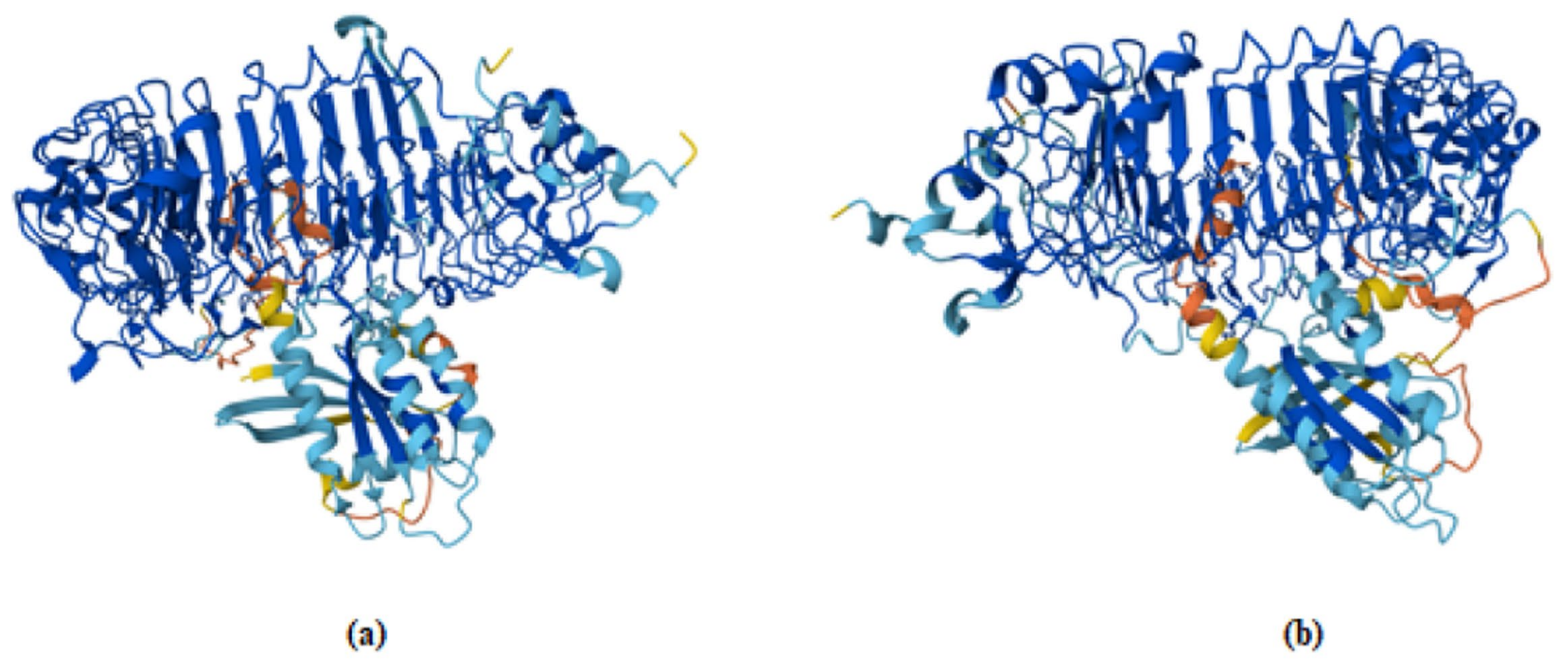
The docking structure of **(a)** (pan-RAS mRNA cancer vaccine-TLR7) and **(b)** (pan-RAS mRNA cancer vaccine -TLR8) complexes as predicted with Alphafold3 server.

Docking of pan-RAS mRNA cancer vaccine with TLR7/8 is carried out with Alphafold3 sever. The ipTM = 0.21pTM = 0.77 is for (pan-RAS mRNA cancer vaccine -TLR7) complex and ipTM = 0.21pTM = 0.76 is observed for (pan-RAS mRNA cancer vaccine -TLR8) complex. The predicted template modeling (pTM) score and the interface predicted template modeling (ipTM) score are both based on the template modeling (TM) score, which measures how accurate a protein structure is (71, 72). A pTM score above 0.5 suggests that the overall fold of the predicted complex is likely close to the real structure. ipTM focuses on how well the relative positions of different subunits in the complex are predicted with Scores above 0.8 mean the prediction is very reliable; Scores below 0.6 usually mean the prediction failed and Scores between 0.6 and 0.8 fall into a gray area where the prediction could go either way. Since TM scoring is very strict for small proteins or short chains, pTM scores can be very low (<0.05) when fewer than 20 residues are involved. See Figure 4.

**Figure 5:**
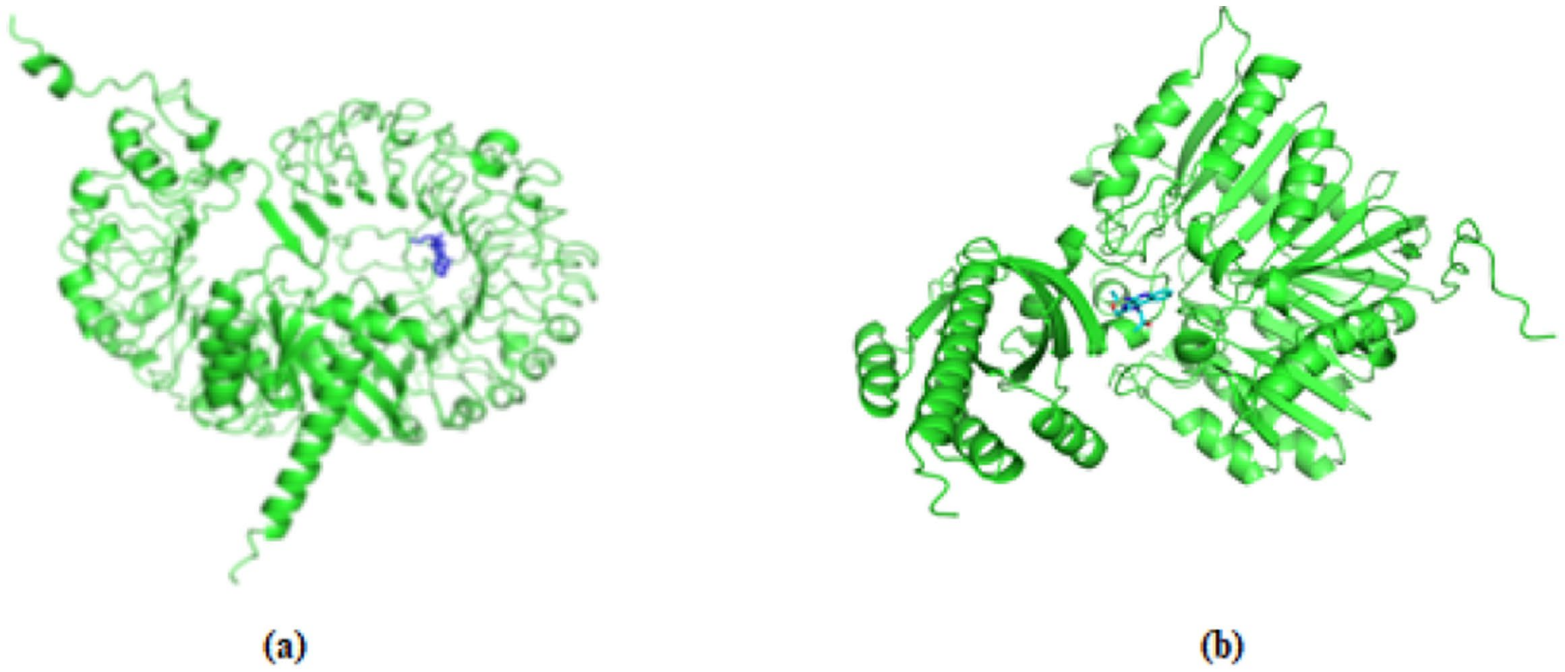
The **(a)** docked structure of **(a)** (TLR7-pan-RAS mRNA cancer vaccine-resiquimod) and **(b)** (TLR8-pan-RAS mRNA cancer vaccine-resiquimod) complex with HDOCK server. The vaccine is represented in blue color.

The docking structure of (TLR7/8–resiquimod–pan-RAS mRNA vaccine) as predicted by HDOCK server is given in Figure 5.

### 3.10 Molecular dynamics simulation analysis of TLR7/8–resiquimod–Pan-RAS mRNA vaccine complexes

**Figure 6:**
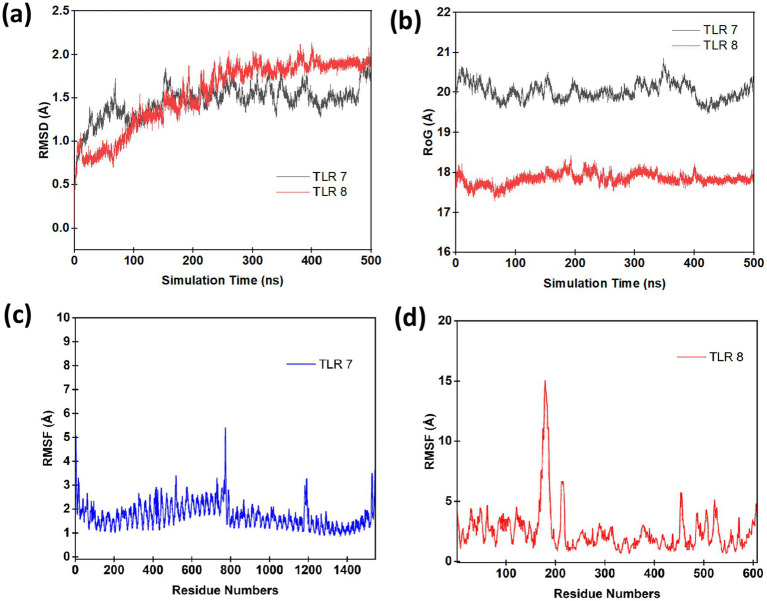
Molecular dynamics (MD) simulation of TLR7 and TLR8 complexes with resiquimod and the pan-RAS mRNA vaccine over 500 ns. **(a)** RMSD profiles showing early equilibration and stable conformations for TLR7 (black) and TLR8 (red). **(b)** RoG plots indicating overall structural compactness, with TLR8 (~17.9 Å) more compact than TLR7 (~20.1 Å). **(c,d)** RMSF profiles showing limited residue fluctuations (<2 Å) and minor loop flexibility near the ligand–binding regions, confirming stable ligand–receptor interactions.

To elucidate the conformational stability and dynamic behaviour of TLR7 and TLR8 in complex with Resiquimod and the pan-RAS mRNA cancer vaccine, 500 ns all-atom molecular dynamics (MD) simulations were performed under explicit solvent conditions. The trajectories were analysed for structural stability, compactness, and residue-level flexibility to evaluate receptor adaptability and ligand-induced conformational changes. The root mean square deviation (RMSD) revealed stabilization throughout the production phase. The TLR7 complex displayed an average RMSD of approximately 1.6 Å, while the TLR8 complex showed a slightly higher deviation of ~1.9 Å, suggesting enhanced conformational flexibility upon ligand binding. The radius of gyration (RoG) demonstrated consistent compactness, with TLR7 maintaining an average RoG of ~20.1 Å and TLR8 exhibiting ~17.9 Å, indicative of stable folding and minimal expansion of the tertiary structure. The slightly lower RoG value for TLR8 implies a more compact conformation, correlating with its tighter ligand accommodation and stronger noncovalent interactions, consistent with ONIOM-derived electronic polarization results. The root mean square fluctuation (RMSF) analysis quantified the flexibility of individual residues within the receptor structures. Most residues showed minimal fluctuation (<2 Å), confirming stable secondary structure maintenance. Localized peaks in the RMSF profile of TLR8 correspond to surface loops near the ligand binding region, which are typically associated with allosteric adjustments facilitating ligand recognition. Similarly, minor fluctuations observed in TLR7 loops reflect adaptive flexibility for ligand-induced signalling activation. See Figure 6.

**Figure 7:**
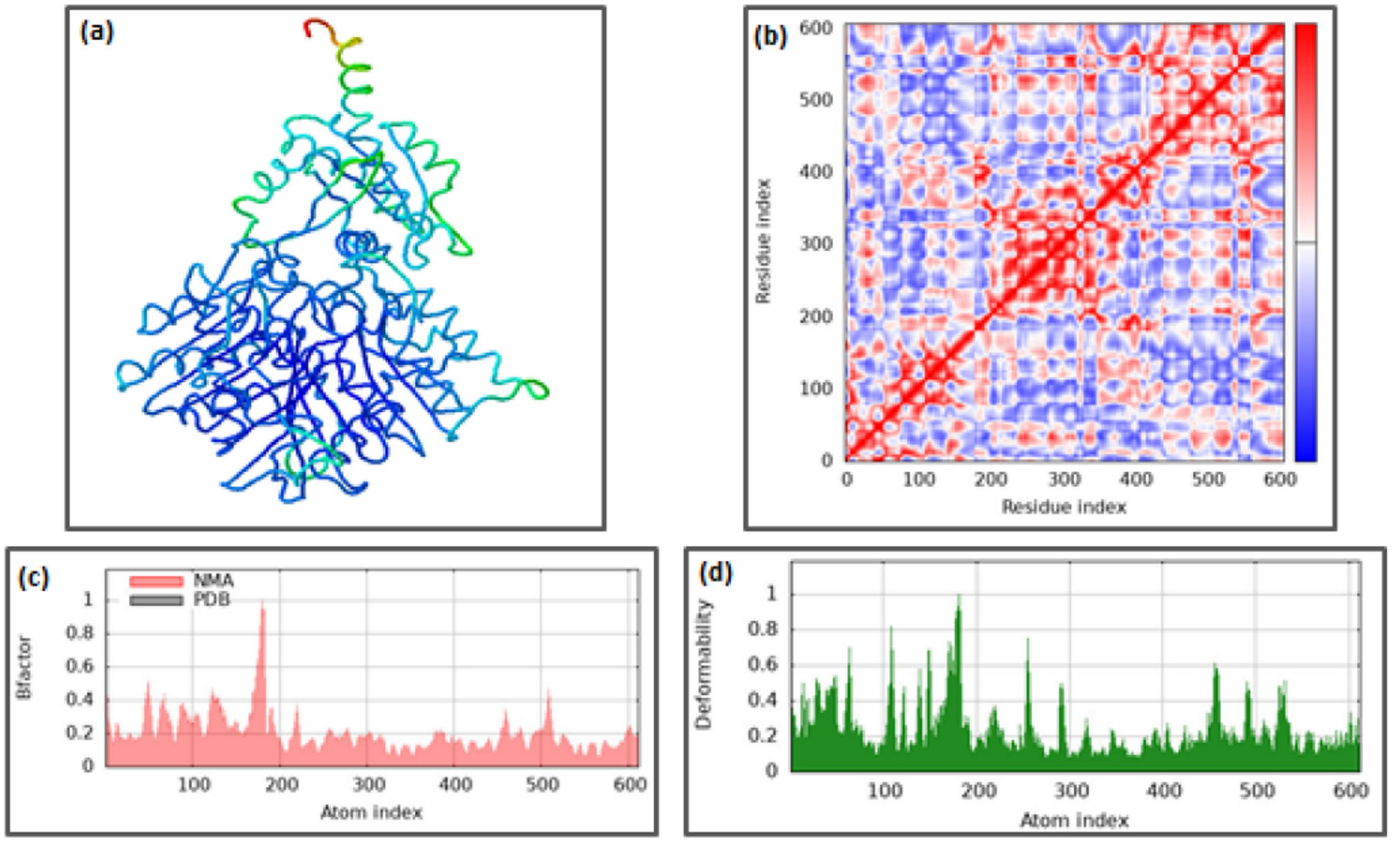
Normal mode analysis for (TLR7-pan-Ras mRNA cancer vaccine-resiquimod) complex: **(a)** Docked structure, **(b)** Covariance matrix, **(c)** B-factor graph, and **(d)** Deformability graph (iMods server).

**Figure 8:**
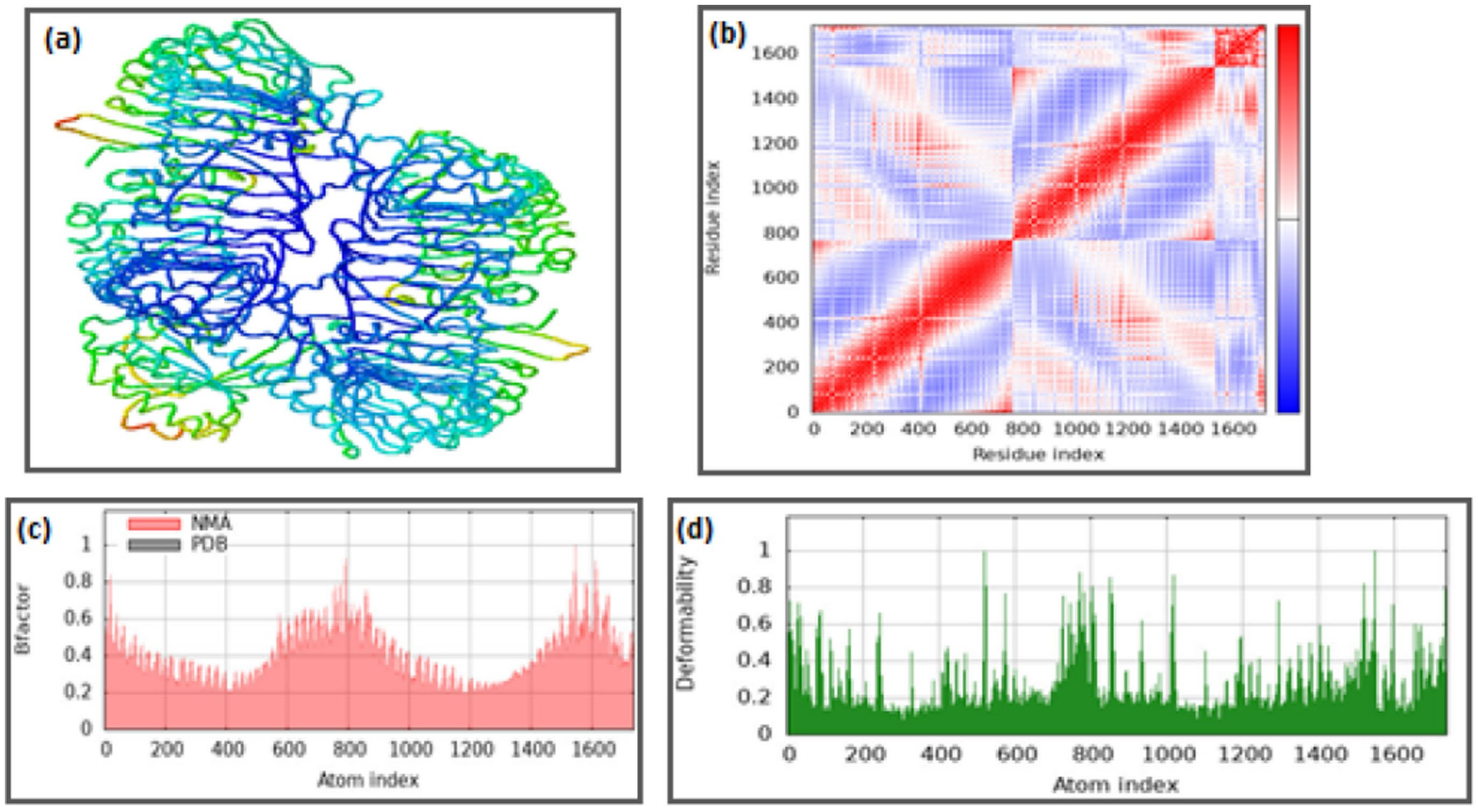
Normal mode analysis for (TLR7-pan-RAS mRNA cancer vaccine-resiquimod) complex: **(a)** Docked structure, **(b)** Covariance matrix, **(c)** B-factor graph, and **(d)** Deformability graph (iMods server).

To further characterize the conformational dynamics, normal mode analysis (NMA) was performed to investigate the collective motions within the complexes. The deformability showed coordinate deviations within 0–1 Å, indicating limited flexibility confined to loop regions without perturbing the overall structural core. B-factor mobility profiles predicted atomic displacements within 0–1 Å, consistent with the MD-derived RMSF trends, confirming the robustness of both TLR7 and TLR8 during ligand engagement. See Figures 7 and 8 respectively. Additionally, the elastic network models depicted dense inter-residue connections, particularly around the binding interface, signifying strong cooperativity and structural coupling between the ligand and receptor residues. Collectively, these MD and NMA results indicate that the (TLR7/8–resiquimod–pan-RAS mRNA vaccine) complexes attain a stable and energetically favorable conformation, with restricted residue flexibility and robust intramolecular connectivity. The observed differences in RMSD and RoG between TLR7 and TLR8 highlight receptor-specific adaptations, wherein TLR8 exhibits greater compactness and electronic polarization, suggesting a stronger and more stable binding interaction with resiquimod and the pan-RAS mRNA vaccine components.

**Figure 9:**
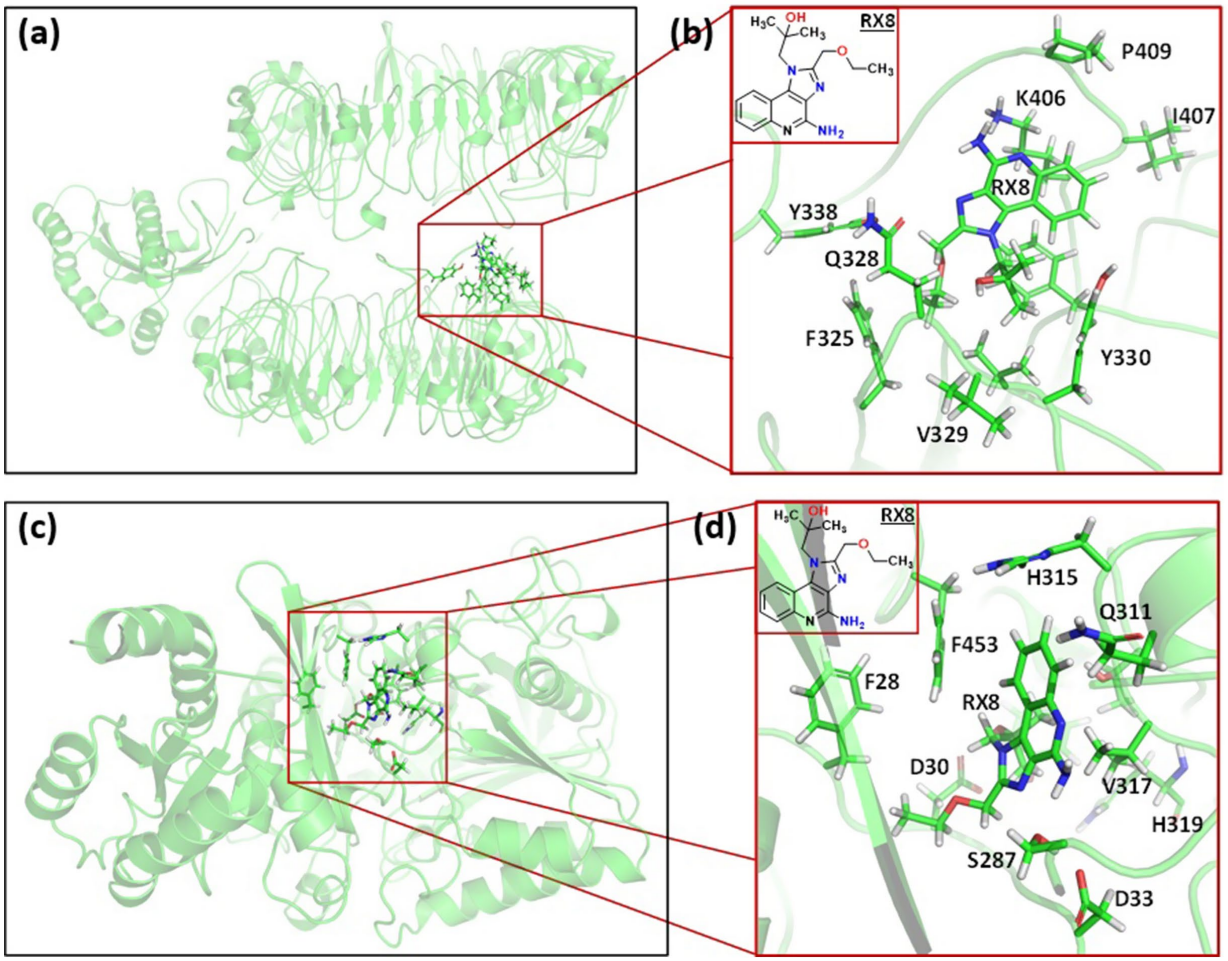
Binding interactions of **(a)** resiquimod (R848) within TLR7; **(b)** resiquimod (R848) within the TLR7 binding pocket; **(c)** resiquimod (R848) within TLR8; **(d)** resiquimod (R848) within the TLR8 binding pocket.

*Binding Site Analysis of TLR7 and TLR8 with Resiquimod:* The most populated (after Clustering of 500 ns simulations) snapshot were taken for structural visualization of the (TLR7–resiquimod) and (TLR8–resiquimod) complexes revealed distinct but complementary binding patterns within their respective active sites (Figure 9). In the TLR7–resiquimod complex, the ligand is deeply embedded within the hydrophobic cleft located between the leucine-rich repeat (LRR) domains. Key hydrogen bonding interactions were observed between the imidazoquinoline ring of resiquimod and polar residues such as K406, Y338, and Q328, which stabilize the ligand orientation. Additionally, hydrophobic contacts with residues V329, P409, and F325 contribute to the structural rigidity of the complex. The hydrogen bond network formed between the ligand’s hydroxyl and amine functional groups and the side chains of TLR7 residues enhances electrostatic complementarity and charge delocalization within the active site. The overall configuration reflects a stable and well-defined binding pocket conducive to receptor activation. The interaction geometry supports the observed electronic polarization and the smaller HOMO–LUMO gap (4.574 eV), confirming strong ligand–receptor coupling and structural integrity at the energy-minimized state.

In the TLR8–resiquimod complex, the ligand is situated within a broader yet deeper binding groove formed at the junction of the LRR domains. Here, the amine and carbonyl groups of Resiquimod engage in strong hydrogen bonds with F453, H315, and H319, while *π*–π stacking interactions with F453 and Y611 further stabilize the aromatic core of the ligand. Several nonpolar residues, including F28, and V317, form van der Waals contacts that enhance the overall hydrophobic stabilization of the complex. The compactness of the TLR8 binding cavity, as confirmed by the lower average RoG value (~17.9 Å), facilitates denser packing of residues and stronger intermolecular forces. This corresponds to a smaller electronic gap (4.023 eV), reflecting increased charge transfer and enhanced electronic communication between the ligand and the receptor. The optimized interactions observed in TLR8 suggest a higher binding affinity and stronger activation potential compared to TLR7, consistent with molecular dynamics findings and normal mode analysis showing greater structural rigidity and cooperative domain motions upon ligand binding.

**Figure 10:**
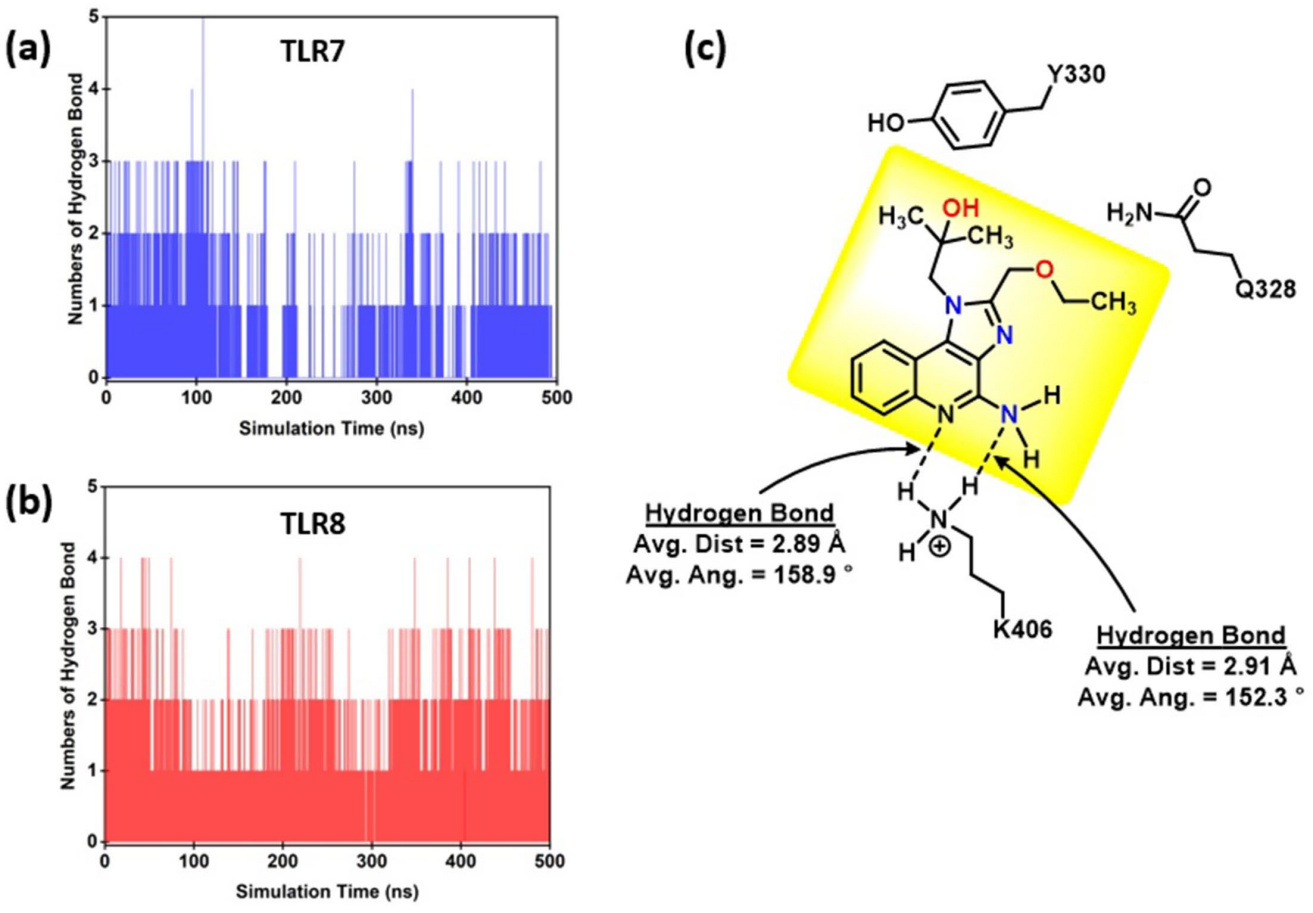
Hydrogen bond analysis of resiquimod (R848) interactions with TLR7 and TLR8 during 500 ns MD simulation. Bar plots show the number of hydrogen bonds formed over time for **(a)** TLR7 and **(b)** TLR8 complexes. **(c)** ChemDraw schematic illustrates key donor and acceptor atoms.

Hydrogen Bond Interaction Analysis: To investigate the nature and persistence of weak interactions between R848 and TLR7/8, hydrogen bond analyses were performed over the 500 ns MD trajectories. The hydrogen bonding frequency, average donor–acceptor distances, and angles were calculated to assess the strength and stability of these noncovalent interactions (Figure 10). In the TLR7–resiquimod complex, several stable hydrogen bonds were observed throughout the simulation, primarily involving K406, Q328, Y338, P409, and V329. The strongest and most persistent hydrogen bonds were formed between the nitrogen atoms of resiquimod (R848_1545@N1) and the terminal amine hydrogens of K406 (HZ1–HZ3), with a cumulative occupancy of approximately 0.06 and average bond distances around 2.89 Å, and bond angles near 158°, indicating robust electrostatic stabilization. Additional hydrogen bonds between R848_1545@N2 and Q328@NE2 (occupancy 0.011, distance 2.93 Å) and Y338@OH (occupancy 0.0079, distance 2.85 Å) contribute to maintaining the ligand’s orientation within the hydrophobic cavity. Weak but consistent interactions were also detected with P409 and V329, reflecting the dynamic nature of secondary contacts that enhance overall binding affinity. See Figure 10.

In the TLR8–resiquimod complex, hydrogen bonding was more widely distributed across the receptor interface, suggesting a greater degree of adaptive interaction. The dominant contacts included those between R848_609@O1 and Y196@OH (distance 2.81 Å, angle 162°), as well as interactions between R848_609@N1 and R41@NH1 (distance 2.90 Å, angle 158°) and Q311@NE2 (distance 2.90 Å, angle 161°). These hydrogen bonds remained stable for a significant portion of the simulation, facilitating optimal positioning of the ligand within the binding cleft. Additional weaker contacts were observed with S287, S39, D30, and R474, characterized by average donor–acceptor distances between 2.78–2.93 Å, reinforcing structural stability through transient hydrogen networks. The slightly lower HOMO–LUMO gap (4.023 eV) and compactness of TLR8 correlate with this extensive hydrogen bonding network, highlighting enhanced charge delocalization and electronic communication between ligand and receptor residues. The average distance (Å) and average bond angle (°) for the donor (resiquimod) and receptors (TLR7/8–pan-RAS mRNA vaccine) complexes are given in Supplementary Tables 3 and 4, respectively.

### 3.11 Electronic structure analysis using ONIOM methodology

**Figure 11:**
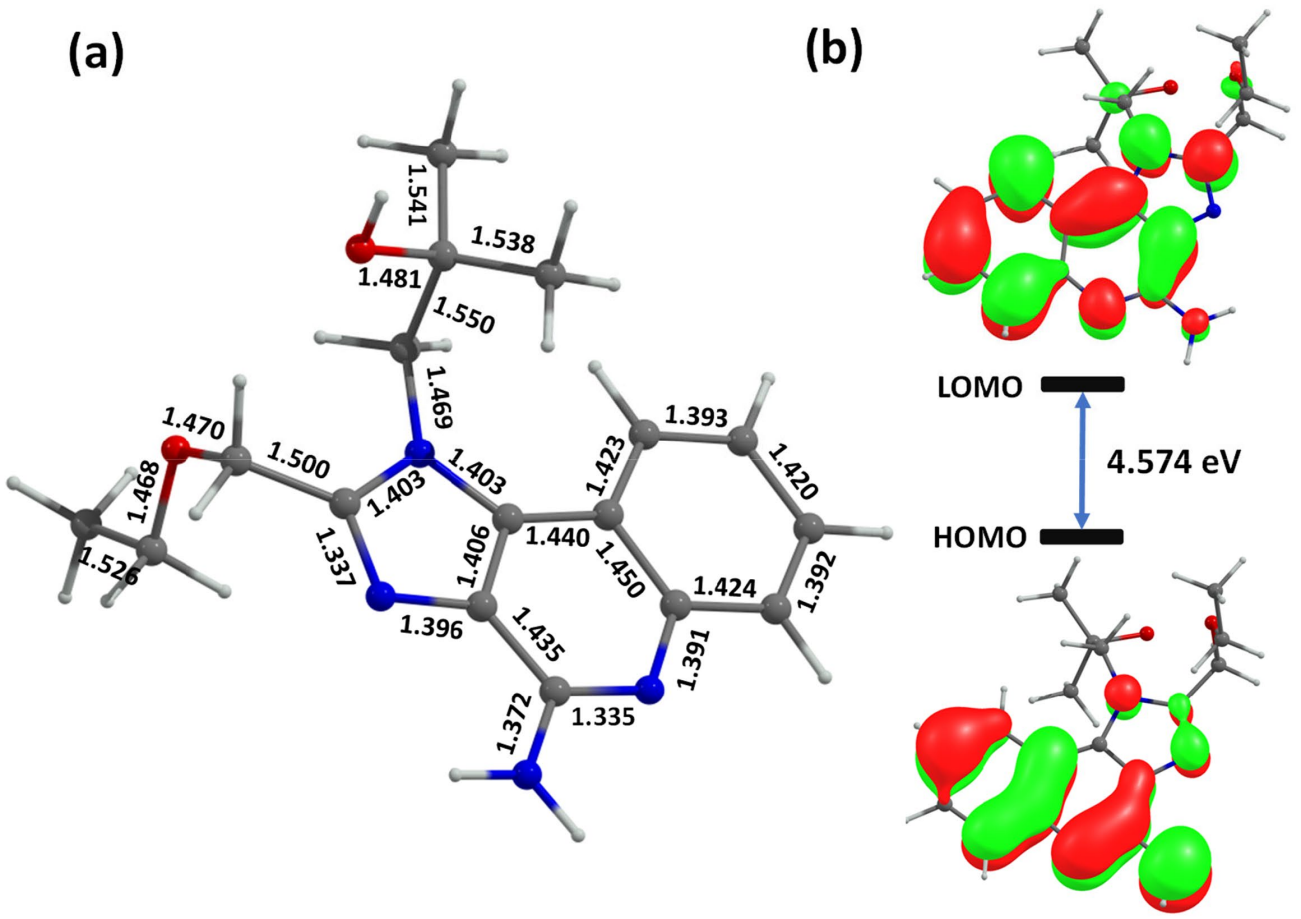
The **(a)** optimized QM region and **(b)** frontier molecular orbitals (HOMO and LUMO) of the drug–receptor complex obtained from ONIOM (B3LYP/LANL2DZ: Amber) calculations.

Following the molecular dynamics simulations, ONIOM-based quantum mechanical/molecular mechanical (QM/MM) calculations were performed to gain deeper insights into the electronic structure and noncovalent interactions governing drug–receptor and vaccine–protein binding. These calculations were executed using Gaussian16 Rev. B.01, employing the B3LYP functional (73) with the LANL2DZ (74) basis set, augmented by Grimme’s D3 dispersion (75) correction to account for long-range van der Waals interactions. The QM region comprised the bound ligand and key residues within the binding pocket, while the surrounding protein matrix was treated at the molecular mechanics level using the AMBER force field parameters extracted from the equilibrated MD trajectory. The primary objective of these hybrid ONIOM calculations (76) was to elucidate the electronic structure, charge distribution, and weak interaction networks (including hydrogen bonding, *π*–π stacking, and electrostatic stabilization) that stabilize the drug within the active site. The HOMO–LUMO energy gap was computed to assess electronic stability and reactivity of the ligand in both TLR7 and TLR8 complexes. See Figure 11.

The results revealed a HOMO–LUMO gap of 4.574 eV for the TLR7–R848 complex and 4.023 eV for the TLR8–R848 complex, indicating enhanced electronic delocalization and stronger polarization interactions in the TLR8 binding pocket. The smaller energy gap for TLR8 suggests greater electronic flexibility, which may contribute to more effective receptor activation and drug binding affinity. Frequency calculations confirmed the absence of imaginary modes, validating the optimized geometries as true energy minima. The optimized structures exhibited distinct conformational stabilization driven by intramolecular hydrogen bonds between the drug’s heteroatoms and key amino acid residues, alongside π–cation interactions involving aromatic side chains. The inclusion of dispersion corrections was essential to accurately capture stacking interactions within the hydrophobic regions of the binding site, particularly near the imidazole and purine moieties of the ligand. The frontier molecular orbitals (HOMO and LUMO) demonstrated that electron density in the TLR7-bound R848 was primarily localized on the aromatic framework, whereas in the TLR8-bound complex, it extended toward the polar functional groups interacting with charged residues, enhancing polarization and binding strength. These observations are consistent with the electrostatic field variations observed during the MD simulations, further supporting the notion that electronic polarization and charge redistribution play a pivotal role in stabilizing the drug–receptor interface. The optimized geometries, frontier orbital visualizations, and key bond parameters are presented in Figure 11, illustrating the spatial orientation of the drug within both TLR7 and TLR8 active sites. These findings provide quantum-level insights into the structural basis of ligand recognition and stabilization, complementing the molecular dynamics results and supporting the observed immunoinformatics predictions.

### 3.12 Codon optimization

The original GC content: 40.89% for the species: *Homo Sapiens*. The CAI-Value of the improved sequence: 0.95; GC-Content of the improved sequence: 61.72% for the species: *Homo Sapiens*. Similarly, the original GC content: 41.75% for the *mus-musculus*. The CAI-Value of the improved sequence: 0.72 and GC-Content of the improved sequence: 59.78% for *mus musculus.*

### 3.13 Immune simulation of mRNA cancer vaccine

**Figure 12:**
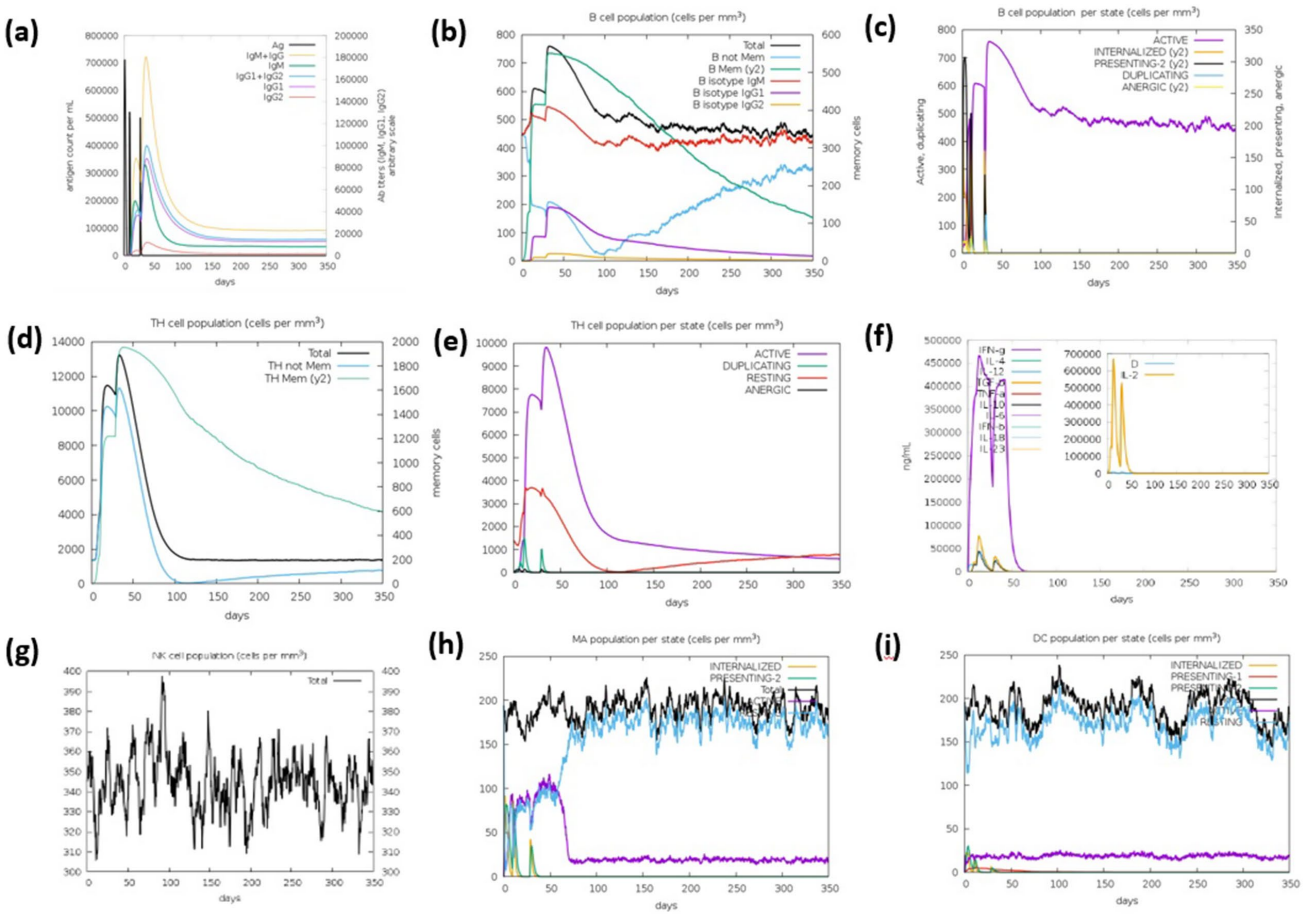
Immune response simulation elicited by three injections of the Comirnaty vaccine construct (WLcd1) generated by C-ImmSim server. **(a)** Titers of different antibodies. **(b)** Levels of B cell population. **(c)** Levels of plasma B cells. **(d)** Levels of helper T-cell population. **(e)** Levels of cytotoxic T cell population. **(f)** Concentration of cytokines. **(g)** Levels of NK cell population. **(h)** Levels of MA population. **(i)** Levels of DC population.

Immunoinformatics analysis using the CImmSim server predicted strong immune responses to the pan-Ras cancer vaccine, characterized by elevated levels of IgM, IgG1, and IgG3 antibodies, along with increased populations of B cells, helper T cells, and cytotoxic T cells. Immune response graphs also revealed heightened levels of cytokines, interleukins, and natural killer (NK) cells, indicating sustained adaptive immunity (Figure 12). Immune simulations were conducted to understand the dynamics of antibody production and cell-mediated immune responses following vaccination, mapping the initiation and progression of immune activity within the host. Simulation results from the C-ImmSim server indicated a robust induction of immune responses following administration of the vaccine. The study predicted that the primary immune response to the antigenic fragments increased progressively after each of the three vaccine doses, as evidenced by rising concentrations of multiple immunoglobulins. Secondary immune responses were also enhanced, demonstrating effective priming from the initial exposure. Elevated levels of IgM and IgG reflected a strong humoral response (Figure 12a). The B cell population exhibited an increase in memory B cells and IgM isotypes, accompanied by a reduction in antigen concentration (Figures 12b,c), indicating improved antigen clearance during subsequent exposures (Figures 12d,e).

Additionally, the vaccine elicited substantial activation of TH1 and TC cell populations, along with memory formation (Figure 12f). Increased numbers of dendritic cells and macrophages suggested efficient antigen presentation by these APCs (Figure 12g,h). The construct also stimulated production of multiple cytokines, including IFN-*γ*, IFN-*β*, IL-10, and IL-23, which are critical for initiating and sustaining immune responses (Figure 12i). These findings were consistent with the observed IFN-γ induction following vaccination. Furthermore, the low Simpson diversity index (D) indicated a broad and varied immune response, highlighting the vaccine’s capacity to generate diverse immunological activity (Figure 12i).

### 3.14 Vaccines’ safety profile

Blastp server showed alignment score with more than 100 domains, which suggest that the mRNA-encoded protein may have cross-reactivity potential, highlighting the need for careful epitope selection, immunogenicity testing, and safety evaluation before clinical application. If predicted epitopes are shared across multiple unrelated proteins, the immune system may not selectively target the intended tumor antigen, reducing therapeutic efficacy and increasing unintended reactivity. BLASTp hits against proteins from pathogens, toxins, or allergens may indicate the potential for allergic or toxic responses, depending on the nature of the aligned sequences (See Supplementary Table 5).

### 3.15 Toxicity prediction for resiquimod

**Figure 13:**
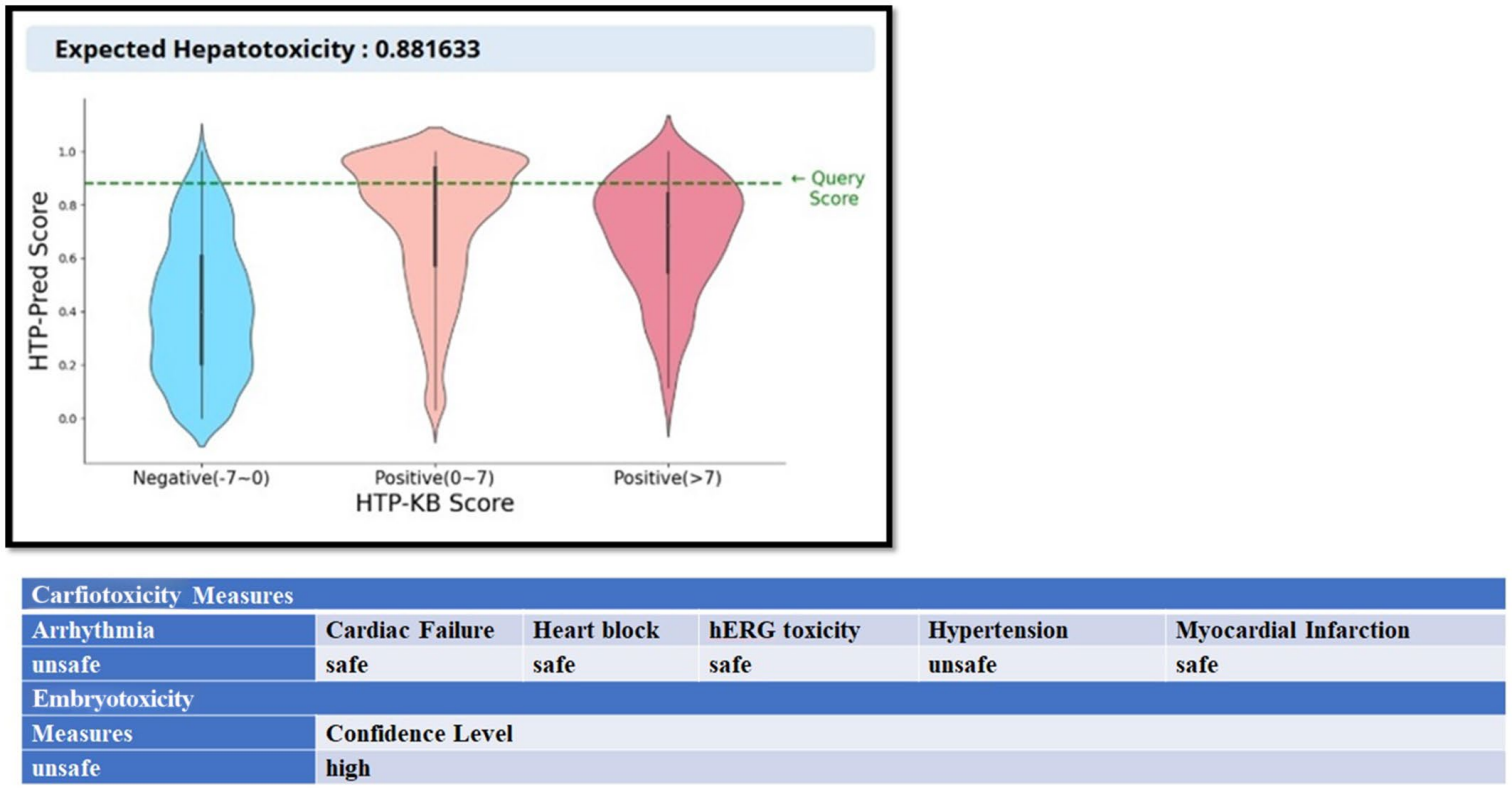
The liver toxicity (HepatoToxicity Portal), cardiotoxicity (CardioToxCSM web server), and embryotoxicity (EmbryoTox webserver) measures of resiquimod.

The expected Hepato-toxicity is 0.881633 and the cardiotoxicity predicts that it is safe for Cardiac failure, Heart Block, herG toxicity and Myocardial Infarction while it is unsafe for Arrhythmia and Hypertension. The embryotoxicity results predict its unsafe use during pregnancy. See Figure 13.

## 4 Discussion

The translational relevance of such immunoinformatics-guided vaccine design is supported by recent experimental studies. In a syngeneic hemispleen model of PDAC, KPC tumor cells implanted into the liver were targeted by an mRNA vaccine encoding five 25-mer peptides. Among the predicted H2Kb- and H2Db-binding epitopes, LYPFPLAL was validated as a functional CD8^+^ T cell epitope through IFN-*γ* ELISpot assays, confirming its capacity to elicit antigen-specific T cell responses. Such epitopes provide valuable surrogate markers for monitoring vaccine efficacy *in vivo*. However, PDAC progression is strongly shaped by its immunosuppressive TME, enriched with Tregs, M2 macrophages, neutrophils, and MDSCs, alongside fibrotic stroma that limits immune infiltration. The high prevalence of liver metastases in PDAC further exacerbates immune evasion by fostering tolerance and suppressing CD8^+^ T cell activity, underscoring the need for vaccines capable of restoring systemic antitumor immunity (77).

Clinically, multiple mRNA-based cancer vaccines are under evaluation for PDAC and other solid tumors. Trials such as NCT03468244, NCT03948763, and NCT03953235 have tested KRAS-targeting mRNA vaccines alone or in combination with checkpoint inhibitors (pembrolizumab, nivolumab, ipilimumab), with subsets of patients demonstrating KRAS mutation-specific CD8^+^ T cell responses. BioNTech’s personalized vaccine RO7198457 (BNT122; NCT04161755) combined with atezolizumab and FOLFIRINOX elicited neoantigen-specific T cell expansion in half of treated patients, while the AMPLIFY-7P Phase 2 trial (NCT05726864) is now investigating the ELI-002 7P vaccine targeting seven KRAS/NRAS peptides (78). Collectively, these data emphasize that pan-RAS mRNA vaccines, particularly when combined with immune modulators such as TLR7/8 agonists, hold strong promise for overcoming PDAC’s immunosuppressive barriers and improving clinical outcomes, though careful expert-led assessment remains essential for their safe and effective translation. Beyond mRNA-based approaches, multiple vaccine platforms have been explored in pancreatic cancer with varying degrees of success (79–85). Dendritic cell (DC)-based vaccines, such as CellgramDC-WT1 (CDW), which pulse DCs with WT1 peptides and zoledronate, demonstrated enhanced IL-12 and IFN-γ secretion and stronger CTL responses, though larger clinical studies are required to establish efficacy (86). Tumor cell lysate-pulsed DC vaccines, administered with gemcitabine, induced antitumor immunity and prolonged survival, but the reliance on sufficient autologous tumor tissue limits feasibility (87). To overcome this, early-phase studies with allogeneic tumor lysate-loaded DCs in resected PDAC showed safety, feasibility, and CTL activation. Whole tumor cell vaccines, including GVAX (88) and Algenpantucel-L (89), have generated only modest immune responses, likely due to MHC polymorphism and limited epitope diversity (90–92). Peptide-based vaccines offer more targeted antigen presentation. KRAS is considered an optimal candidate, with TG01/GM-CSF emerging as a leading synthetic long peptide (SLP) vaccine (93). In a Phase 1/2 trial, TG01 combined with gemcitabine achieved a median overall survival of 34.1 months in resected PDAC patients, accompanied by robust immune responses (94). Ongoing trials (NCT04117087, NCT05013216) are further evaluating KRAS-targeted SLP vaccines in combination with checkpoint inhibitors or in high-risk populations. Other antigenic targets, such as telomerase (GV1001) and p53 (p53MVA), have shown immunogenicity but limited efficacy, often requiring adjuvants or checkpoint blockade for durable responses (95). Alternative platforms, including microorganism-based vaccines like GI-4000 (*S. cerevisiae* expressing mutant RAS) and viral vectors (adenovirus, herpesvirus, lentivirus, myxoma virus), have demonstrated safety and immunogenicity in early studies but limited clinical benefit. Novel strategies such as iExosomes delivering siRNA/shRNA against KRAS (G12D) (NCT03608631), ENO1 DNA vaccination in preclinical KPC models, and the VEGFR-2–targeting DNA vaccine VXM01 (NCT01486329) highlight the breadth of innovation in PDAC immunotherapy. Taken together, these diverse approaches underscore both the promise and challenges of vaccine development for PDAC (96–117). While DC, peptide, whole-cell, and microorganism-based vaccines show variable efficacy, mRNA vaccines particularly those targeting shared driver mutations like KRAS and combined with immunomodulators such as TLR7/8 agonists offer a rational path forward to overcome PDAC’s immunosuppressive microenvironment. The integration of epitope-driven design, rigorous preclinical validation, and carefully structured clinical trials will be essential to translate these vaccines into effective precision therapies for PDAC (118).

Recent advances in immunotherapy highlight two complementary strategies for overcoming the immunologically “cold” tumor microenvironment of pancreatic ductal adenocarcinoma (PDAC). On one side, individualized neoantigen mRNA–lipoplex vaccines such as *autogene cevumeran* have shown encouraging results in clinical trials by inducing long-lived, tumor-specific CD8^+^ T cell clones and prolonging recurrence-free survival when combined with surgery, checkpoint blockade, and chemotherapy. These findings emphasize the potential of durable T cell immunity in controlling PDAC recurrence. Parallel to this, preclinical studies targeting innate immunity demonstrate that TLR7/8 activation can reshape the PDAC tumor microenvironment to support adaptive responses (119–121). Agonists such as resiquimod (R848), especially when combined with radiotherapy, enhance tumor-specific CD8^+^ T cell infiltration, reduce regulatory T cells, and promote systemic immune surveillance against metastases (122). Similarly, nanocarrier-based platforms combining KRAS-targeting mRNA with TLR7/8 agonists further amplify antigen presentation and cytotoxic T cell responses, suppressing tumor growth and improving survival in orthotopic PDAC models. Together, these approaches suggest that vaccines providing tumor-specific antigens and TLR7/8 agonists boosting innate and adaptive immune crosstalk may be synergistic strategies for PDAC therapy. Whereas the vaccine trial demonstrates the feasibility of long-term, mutation-directed T cell memory in patients, TLR7/8-based interventions reveal how innate activation can overcome immune suppression and broaden T cell responses. This integration underscores the promise of combining personalized vaccines with innate immune modulators to achieve durable disease control in PDAC (123–126).

mRNA-5671 is a lipid nanoparticle–formulated, tetravalent mRNA vaccine designed to target four of the most common KRAS driver mutations G12D, G13D, G12C, and G12V. Preclinical studies have demonstrated that immunization with this construct elicits strong CD8^+^ T cell responses against KRAS-mutant antigens. Currently, a Phase I clinical trial (NCT03948763) is evaluating its safety and efficacy in patients with advanced or metastatic non-small cell lung cancer, colorectal cancer, and pancreatic adenocarcinoma harboring KRAS mutations, both as a monotherapy and in combination with the PD-1 inhibitor pembrolizumab. This approach aligns with the broader concept of pan-RAS mRNA cancer vaccines, which aim to cover a spectrum of oncogenic RAS mutations driving multiple malignancies. By encoding several high-frequency KRAS variants, mRNA-5671 represents a prototype for pan-RAS vaccination strategies that could provide mutation-specific immune surveillance across different cancer types, including PDAC, where KRAS mutations dominate the mutational landscape (127). In the broader landscape of cancer vaccine development, several clinical trials are underway to evaluate personalized and shared neoantigen vaccines across multiple tumor types. For example, the EVAX-01-CAF09b vaccine (NCT03715985), developed using the PIONEER platform, is being tested in metastatic malignant melanoma, NSCLC, and bladder urothelial cancer, incorporating 5–15 peptides. Agenus Inc. also investigated ASV® AGEN2017 with QS-21 Stimulon® Adjuvant in solid tumors, though enrollment was limited to three patients (NCT03673020, NCT02992977). Similarly, NCT04509167 employs Montanide ISA-51 VG as an adjuvant, while NCT03480152, conducted in four patients with metastatic melanoma and colon cancer, reported enhanced T cell responses but no objective tumor regression. Checkpoint inhibitors are frequently integrated into these vaccine trials. For instance, pembrolizumab is being studied with neoantigen vaccines in multiple cancers (NCT03568058), with immune responses assessed at different time points relative to vaccination. Genocea Biosciences is conducting another trial (NCT03633110) combining vaccines with pembrolizumab and nivolumab, while Gritstone Bio has launched several studies (NCT03639714, NCT03794128) testing personalized vaccines GRT-C901 and GRT-R902 alongside nivolumab and ipilimumab in NSCLC, colorectal, gastroesophageal, and urothelial cancers. Interim findings from these studies suggest improved survival outcomes and robust T cell activation. In addition, trials in China (NCT03662815) showed favorable safety and immune responses in 80% of peptides tested, although another dendritic-cell–based pan-cancer vaccine trial (NCT03300843) was terminated due to poor accrual (128).

Other innovative approaches include the VB10. NEO vaccine combined with bempegaldesleukin (NKTR-214) (NCT03548467), which delivers 14 vaccinations per patient, with primary endpoints assessing safety and tolerability, and secondary endpoints measuring immunogenicity, objective response rate, progression-free survival, and overall survival. Collectively, these efforts mirror the strategies seen with mRNA-5671 and pan-RAS mRNA vaccines in PDAC, where targeting recurrent oncogenic mutations or patient-specific neoantigens often enhanced with TLR7/8 agonists or immune checkpoint blockade represents a promising paradigm. Together, these clinical trials emphasize that effective cancer vaccination likely requires multi-modal combinations: vaccines for antigen priming, checkpoint inhibitors to overcome immune suppression, and adjuvants or TLR agonists to amplify durable T cell responses (129).

## 5 Conclusion

The pancreatic ductal adenocarcinoma (PDAC) remains a challenging malignancy due to its high prevalence of KRAS mutations and an immunosuppressive tumor microenvironment. Immunoinformatics-guided pan-RAS and neoantigen mRNA vaccines have shown strong potential to elicit durable, mutation-specific CD8^+^ T cell responses, with preclinical and early clinical studies demonstrating prolonged immune memory and tumor control. TLR7/8 agonists, such as resiquimod (R848), can further enhance these responses by activating innate immunity, promoting antigen presentation, and remodeling the tumor microenvironment, particularly when combined with radiation or nanocarrier-based mRNA delivery. Comparisons with other vaccine platforms including dendritic cell, peptide, whole-cell, and microorganism-based approaches underscore the advantages of mRNA vaccines in targeting multiple mutations efficiently while maintaining safety and immunogenicity. Integration with checkpoint inhibitors and adjuvants further potentiates anti-tumor immunity, as shown in ongoing pan-cancer and PDAC trials. Collectively, these findings suggest that personalized or pan-RAS mRNA vaccines, when combined with innate immune modulators and immunotherapy, offer a promising precision medicine approach for PDAC and other KRAS-driven cancers, warranting continued clinical investigation to optimize efficacy and long-term patient outcomes.

## Data Availability

The original contributions presented in the study are included in the article/Supplementary material, further inquiries can be directed to the corresponding author.

## Footnotes

1
https://services.healthtech.dtu.dk/services/NetMHCpan-4.0/

2
https://services.healthtech.dtu.dk/services/NetCTL-1.2/

3
https://www.ddg-pharmfac.net/vaxijen/VaxiJen/

4
https://www.ddg-pharmfac.net/allertop_test/

5
http://crdd.osdd.net/raghava/toxinpred/

6
http://www.iedb.org/

7
https://web.expasy.org/protparam/

8
https://npsa-prabi.ibcp.fr/NPSA/npsa_sopma.html

9
https://alphafoldserver.com/

10
https://prosa.services.came.sbg.ac.at/prosa.php

11
https://saves.mbi.ucla.edu/

12
http://ailab-projects2.ist.psu.edu/bcpred/

13
http://tools.iedb.org/ellipro/

14
http://curie.utmb.edu/getarea.html

15
http://hdock.phys.hust.edu.cn

16
https://alphafoldserver.com/

17
https://www.rcsb.org

18
https://imods.iqf.csic.es/

19
https://www.jcat.de

20
https://kraken.iac.rm.cnr.it/

21
https://blast.ncbi.nlm.nih.gov/

22
https://go.drugbank.com/drugs/DB06530

23
https://biosig.lab.uq.edu.au/cardiotoxcsm

24
https://kobic.re.kr/htp/

25
https://biosig.lab.uq.edu.au/embryotox/

26
https://npsa-prabi.Ibcp.fr/cgi-bin/npsa_automat.pl.?page=/npsa/npsa_gor4.html
