# Supplementary material for: Immunoinformatic insights for design and clinical prospects of pan-RAS cancer vaccine
Source: Front Med (Lausanne). 2026 Mar 5;13:1727976. doi: 10.3389/fmed.2026.1727976 (PMC13000900; doi:10.3389/fmed.2026.1727976)
Supplement: Supplementary file 1 [file Data_Sheet_1.pdf]

# Supplementary Information

## Immunoinformatic Insights for Design and Clinical Prospects of Pan-RAS Cancer Vaccine

Ruby Srivastava* and Thakur Rochak Kumar Rana

Department of Chemistry, Indian Institute of Technology Bombay, Powai, Mumbai 400076, India; Email:amitruby1@gmail.com

**PAN-RAS mRNA VACCINE SEQUENCE:**

**Mutations (**KRAS variants G12D, G13D, L19F, A59T, G60D, Q61H, K117N, and A146T)

MTEYKLVVVGA**DD**VGKSA**F**TIQLIQNHFVDEYDPTIEDSYRKQVVIDGETCLLDILDT**TDH**EEYSAMR
DQYMRTGEGFLCVFAINNTKSFEDIHHYREQIKRVKDSEDVPMVLVGN**N**CDLPSRTVDTKQAQDLARS
YGIPFIETS**T**KTRQRVEDAFYTLYREIRQYFIKKISKEEKTPGCVKIKKCIIM

(The mutations are highlighted (red color) in the mRNA vaccine sequence.

**TLR7- 5GMH (PDB)**

- Classification: IMMUNE SYSTEM
- Organism(s): Macaca mulatta
- Expression System: Drosophila

**TLR8- 5AWB (PDB)**

- Classification: IMMUNE SYSTEM
- Organism(s): Homo sapiens
- Expression System: Drosophila

Source: Protein Data Bank (https://www.rcsb.org/). The models are refined by Alphafold3 server for docking studies.

**Resiquimod:** SMILES notation:
CCOCC1=NC2=C(N)N=C3C=CC=CC3=C2N1CC(C)(C)O.

Source: https://go.drugbank.com/drugs/DB06530 (Drug bank)

Probe radius: 1.400

| Residue | | Total | Apolar | Backbone | Sidechain | Ratio(%) | In/Out |
|---|---|---|---|---|---|---|---|
| MET | 1 | 121.00 | 72.88 | 57.64 | 63.36 | 40.0 | |
| THR | 2 | 53.86 | 33.27 | 8.99 | 44.87 | 42.3 | |
| GLU | 3 | 62.19 | 23.67 | 11.70 | 50.49 | 35.8 | |
| TYR | 4 | 13.09 | 3.80 | 0.00 | 13.09 | 6.8 | i |
| LYS | 5 | 78.42 | 42.50 | 0.01 | 78.41 | 47.7 | |
| LEU | 6 | 0.35 | 0.00 | 0.35 | 0.00 | 0.0 | i |
| VAL | 7 | 2.24 | 2.24 | 0.00 | 2.24 | 1.8 | i |
| VAL | 8 | 0.00 | 0.00 | 0.00 | 0.00 | 0.0 | i |
| VAL | 9 | 2.12 | 2.12 | 0.62 | 1.51 | 1.2 | i |
| GLY | 10 | 1.44 | 0.00 | 1.44 | 0.00 | 1.7 | i |
| ALA | 11 | 13.41 | 13.41 | 0.00 | 13.41 | 20.7 | |
| ASP | 12 | 51.87 | 25.44 | 7.07 | 44.80 | 39.6 | |
| ASP | 13 | 107.89 | 41.08 | 26.57 | 81.32 | 72.0 | o |
| VAL | 14 | 0.65 | 0.48 | 0.21 | 0.45 | 0.4 | i |
| GLY | 15 | 22.22 | 21.66 | 22.22 | 0.00 | 25.5 | |
| LYS | 16 | 14.78 | 9.44 | 1.61 | 13.17 | 8.0 | i |
| SER | 17 | 32.54 | 22.00 | 0.56 | 31.98 | 41.3 | |
| ALA | 18 | 13.87 | 13.75 | 0.56 | 13.31 | 20.5 | |
| PHE | 19 | 0.00 | 0.00 | 0.00 | 0.00 | 0.0 | i |
| THR | 20 | 0.00 | 0.00 | 0.00 | 0.00 | 0.0 | i |
| ILE | 21 | 25.02 | 25.02 | 0.00 | 25.02 | 17.0 | i |
| GLN | 22 | 5.63 | 0.00 | 0.00 | 5.63 | 3.9 | i |
| LEU | 23 | 0.14 | 0.14 | 0.00 | 0.14 | 0.1 | i |
| ILE | 24 | 41.64 | 25.02 | 16.62 | 25.02 | 17.0 | i |
| GLN | 25 | 95.40 | 29.42 | 16.90 | 78.50 | 54.6 | o |
| ASN | 26 | 80.51 | 16.79 | 19.07 | 61.44 | 53.8 | o |
| HIS | 27 | 105.02 | 104.62 | 4.72 | 100.30 | 64.9 | o |
| PHE | 28 | 84.68 | 62.31 | 22.36 | 62.31 | 34.6 | |
| VAL | 29 | 53.26 | 47.43 | 8.38 | 44.88 | 36.7 | |
| ASP | 30 | 120.48 | 30.01 | 33.41 | 87.07 | 77.1 | o |
| GLU | 31 | 141.29 | 52.94 | 11.02 | 130.27 | 92.3 | o |
| TYR | 32 | 144.64 | 111.52 | 29.52 | 115.12 | 59.6 | o |
| ASP | 33 | 107.29 | 36.00 | 6.63 | 100.66 | 89.1 | o |
| PRO | 34 | 86.94 | 75.02 | 13.98 | 72.96 | 69.4 | o |
| THR | 35 | 26.73 | 13.86 | 14.58 | 12.15 | 11.4 | i |
| ILE | 36 | 90.80 | 86.01 | 4.80 | 86.01 | 58.4 | o |
| GLU | 37 | 73.00 | 12.84 | 21.48 | 51.51 | 36.5 | |
| ASP | 38 | 56.27 | 6.90 | 4.25 | 52.02 | 46.0 | |

| | | | | | | | |
|---|---|---|---|---|---|---|---|
| SER | 39 | 79.16 | 24.03 | 23.88 | 55.28 | 71.4 | o |
| TYR | 40 | 46.14 | 35.56 | 6.18 | 39.96 | 20.7 | |
| ARG | 41 | 147.39 | 54.56 | 24.11 | 123.28 | 63.1 | o |
| LYS | 42 | 47.50 | 43.60 | 4.85 | 42.65 | 25.9 | |
| GLN | 43 | 105.24 | 27.29 | 17.69 | 87.54 | 60.9 | o |
| VAL | 44 | 11.82 | 11.82 | 7.92 | 3.90 | 3.2 | i |
| VAL | 45 | 97.86 | 79.71 | 18.15 | 79.71 | 65.2 | o |
| ILE | 46 | 4.22 | 4.22 | 3.52 | 0.70 | 0.5 | i |
| ASP | 47 | 92.61 | 43.42 | 43.45 | 49.16 | 43.5 | |
| GLY | 48 | 71.02 | 39.41 | 71.02 | 0.00 | 81.4 | o |
| GLU | 49 | 78.27 | 40.78 | 5.16 | 73.11 | 51.8 | o |
| THR | 50 | 61.78 | 29.09 | 12.93 | 48.85 | 46.0 | |
| CYS | 51 | 1.74 | 1.74 | 0.00 | 1.74 | 1.7 | i |
| LEU | 52 | 31.17 | 31.17 | 0.00 | 31.17 | 21.3 | |
| LEU | 53 | 0.00 | 0.00 | 0.00 | 0.00 | 0.0 | i |
| ASP | 54 | 23.11 | 1.54 | 1.76 | 21.34 | 18.9 | i |
| ILE | 55 | 0.36 | 0.36 | 0.36 | 0.00 | 0.0 | i |
| LEU | 56 | 25.93 | 25.92 | 0.02 | 25.90 | 17.7 | i |
| ASP | 57 | 0.42 | 0.05 | 0.00 | 0.42 | 0.4 | i |
| THR | 58 | 4.61 | 0.84 | 1.99 | 2.62 | 2.5 | i |
| THR | 59 | 13.48 | 7.62 | 6.94 | 6.54 | 6.2 | i |
| ASP | 60 | 32.93 | 2.76 | 6.98 | 25.95 | 23.0 | |
| HIS | 61 | 83.08 | 64.58 | 6.26 | 76.82 | 49.7 | |
| GLU | 62 | 150.09 | 59.37 | 8.68 | 141.41 | 100.0 | o |
| GLU | 63 | 155.82 | 55.99 | 36.86 | 118.95 | 84.2 | o |
| TYR | 64 | 63.21 | 40.94 | 2.05 | 61.16 | 31.7 | |
| SER | 65 | 66.39 | 39.79 | 1.90 | 64.49 | 83.3 | o |
| ALA | 66 | 86.39 | 80.53 | 15.22 | 71.17 | 100.0 | o |
| MET | 67 | 75.95 | 75.41 | 2.06 | 73.89 | 46.7 | |
| ARG | 68 | 19.12 | 9.19 | 0.00 | 19.12 | 9.8 | i |
| ASP | 69 | 47.83 | 11.12 | 0.00 | 47.83 | 42.3 | |
| GLN | 70 | 116.62 | 32.57 | 2.12 | 114.50 | 79.7 | o |
| TYR | 71 | 16.30 | 12.87 | 1.60 | 14.69 | 7.6 | i |
| MET | 72 | 0.20 | 0.20 | 0.00 | 0.20 | 0.1 | i |
| ARG | 73 | 145.28 | 60.76 | 17.65 | 127.63 | 65.3 | o |
| THR | 74 | 74.38 | 59.40 | 13.27 | 61.12 | 57.5 | o |
| GLY | 75 | 0.79 | 0.75 | 0.79 | 0.00 | 0.9 | i |
| GLU | 76 | 47.32 | 20.40 | 8.35 | 38.97 | 27.6 | |
| GLY | 77 | 0.00 | 0.00 | 0.00 | 0.00 | 0.0 | i |
| PHE | 78 | 0.00 | 0.00 | 0.00 | 0.00 | 0.0 | i |
| LEU | 79 | 0.00 | 0.00 | 0.00 | 0.00 | 0.0 | i |
| CYS | 80 | 0.59 | 0.02 | 0.00 | 0.59 | 0.6 | i |
| VAL | 81 | 0.00 | 0.00 | 0.00 | 0.00 | 0.0 | i |
| PHE | 82 | 0.44 | 0.44 | 0.00 | 0.44 | 0.2 | i |
| ALA | 83 | 3.48 | 3.48 | 0.00 | 3.48 | 5.4 | i |
| ILE | 84 | 1.67 | 1.67 | 0.00 | 1.67 | 1.1 | i |
| ASN | 85 | 47.76 | 1.76 | 15.50 | 32.25 | 28.2 | |
| ASN | 86 | 53.90 | 17.89 | 6.37 | 47.53 | 41.6 | |
| THR | 87 | 58.82 | 51.10 | 1.85 | 56.98 | 53.6 | o |

| | | | | | | | |
|---|---|---|---|---|---|---|---|
| LYS | 88 | 144.43 | 99.86 | 1.43 | 143.00 | 86.9 | o |
| SER | 89 | 0.37 | 0.37 | 0.32 | 0.05 | 0.1 | i |
| PHE | 90 | 12.35 | 11.41 | 0.94 | 11.41 | 6.3 | i |
| GLU | 91 | 109.75 | 46.27 | 15.78 | 93.97 | 66.6 | o |
| ASP | 92 | 34.35 | 6.36 | 2.06 | 32.28 | 28.6 | |
| ILE | 93 | 0.60 | 0.60 | 0.00 | 0.60 | 0.4 | i |
| HIS | 94 | 98.75 | 82.68 | 0.03 | 98.71 | 63.9 | o |
| HIS | 95 | 97.90 | 77.17 | 3.61 | 94.29 | 61.0 | o |
| TYR | 96 | 7.16 | 6.04 | 0.00 | 7.16 | 3.7 | i |
| ARG | 97 | 34.26 | 1.61 | 0.00 | 34.26 | 17.5 | i |
| GLU | 98 | 64.39 | 34.13 | 1.97 | 62.43 | 44.2 | |
| GLN | 99 | 29.86 | 10.08 | 1.22 | 28.64 | 19.9 | i |
| ILE | 100 | 0.00 | 0.00 | 0.00 | 0.00 | 0.0 | i |
| LYS | 101 | 22.64 | 7.36 | 2.08 | 20.56 | 12.5 | i |
| ARG | 102 | 165.99 | 95.41 | 22.32 | 143.67 | 73.5 | o |
| VAL | 103 | 13.36 | 6.63 | 10.53 | 2.83 | 2.3 | i |
| LYS | 104 | 53.88 | 26.25 | 20.51 | 33.36 | 20.3 | |
| ASP | 105 | 137.37 | 35.44 | 25.91 | 111.45 | 98.6 | o |
| SER | 106 | 36.71 | 26.80 | 12.77 | 23.94 | 30.9 | |
| GLU | 107 | 139.97 | 75.13 | 17.86 | 122.11 | 86.5 | o |
| ASP | 108 | 79.25 | 36.41 | 5.30 | 73.95 | 65.4 | o |
| VAL | 109 | 13.04 | 4.24 | 9.02 | 4.02 | 3.3 | i |
| PRO | 110 | 10.39 | 10.39 | 7.47 | 2.92 | 2.8 | i |
| MET | 111 | 4.76 | 4.18 | 0.58 | 4.18 | 2.6 | i |
| VAL | 112 | 0.00 | 0.00 | 0.00 | 0.00 | 0.0 | i |
| LEU | 113 | 0.00 | 0.00 | 0.00 | 0.00 | 0.0 | i |
| VAL | 114 | 0.00 | 0.00 | 0.00 | 0.00 | 0.0 | i |
| GLY | 115 | 0.00 | 0.00 | 0.00 | 0.00 | 0.0 | i |
| ASN | 116 | 0.00 | 0.00 | 0.00 | 0.00 | 0.0 | i |
| ASN | 117 | 26.27 | 1.49 | 0.09 | 26.18 | 22.9 | |
| CYS | 118 | 28.86 | 3.98 | 21.14 | 7.72 | 7.5 | i |
| ASP | 119 | 50.31 | 23.87 | 34.20 | 16.11 | 14.3 | i |
| LEU | 120 | 66.02 | 60.14 | 5.91 | 60.10 | 41.1 | |
| PRO | 121 | 137.38 | 115.04 | 36.84 | 100.54 | 95.6 | o |
| SER | 122 | 67.73 | 60.63 | 14.08 | 53.65 | 69.3 | o |
| ARG | 123 | 84.58 | 42.85 | 24.62 | 59.97 | 30.7 | |
| THR | 124 | 86.83 | 64.20 | 31.87 | 54.96 | 51.7 | o |
| VAL | 125 | 0.67 | 0.65 | 0.14 | 0.53 | 0.4 | i |
| ASP | 126 | 78.00 | 33.86 | 2.12 | 75.88 | 67.2 | o |
| THR | 127 | 47.89 | 40.24 | 0.27 | 47.62 | 44.8 | |
| LYS | 128 | 140.51 | 96.86 | 0.39 | 140.12 | 85.2 | o |
| GLN | 129 | 83.58 | 37.89 | 6.98 | 76.60 | 53.3 | o |
| ALA | 130 | 0.72 | 0.72 | 0.00 | 0.72 | 1.1 | i |
| GLN | 131 | 76.50 | 16.67 | 2.98 | 73.52 | 51.2 | o |
| ASP | 132 | 58.45 | 30.02 | 4.34 | 54.11 | 47.9 | |
| LEU | 133 | 39.84 | 39.81 | 4.56 | 35.28 | 24.1 | |
| ALA | 134 | 3.06 | 3.06 | 0.00 | 3.06 | 4.7 | i |
| ARG | 135 | 170.07 | 92.25 | 18.02 | 152.06 | 77.8 | o |
| SER | 136 | 86.44 | 56.48 | 42.31 | 44.13 | 57.0 | o |

| | | | | | | | |
|---|---|---|---|---|---|---|---|
| TYR | 137 | 37.70 | 18.51 | 19.52 | 18.18 | 9.4 | i |
| GLY | 138 | 68.69 | 40.88 | 68.69 | 0.00 | 78.8 | o |
| ILE | 139 | 25.62 | 17.68 | 10.28 | 15.34 | 10.4 | i |
| PRO | 140 | 40.59 | 40.59 | 13.15 | 27.44 | 26.1 | |
| PHE | 141 | 34.43 | 12.55 | 21.89 | 12.55 | 7.0 | i |
| ILE | 142 | 9.14 | 9.14 | 0.99 | 8.15 | 5.5 | i |
| GLU | 143 | 29.89 | 12.60 | 0.57 | 29.31 | 20.8 | |
| THR | 144 | 0.00 | 0.00 | 0.00 | 0.00 | 0.0 | i |
| SER | 145 | 0.01 | 0.01 | 0.01 | 0.00 | 0.0 | i |
| THR | 146 | 3.62 | 0.00 | 0.14 | 3.48 | 3.3 | i |
| LYS | 147 | 123.57 | 68.66 | 14.91 | 108.67 | 66.1 | o |
| THR | 148 | 83.63 | 63.18 | 29.95 | 53.68 | 50.5 | o |
| ARG | 149 | 77.69 | 45.55 | 9.25 | 68.44 | 35.0 | |
| GLN | 150 | 86.47 | 31.21 | 10.53 | 75.94 | 52.8 | o |
| ARG | 151 | 105.22 | 51.43 | 10.31 | 94.91 | 48.5 | |
| VAL | 152 | 0.05 | 0.00 | 0.05 | 0.00 | 0.0 | i |
| GLU | 153 | 57.22 | 11.41 | 0.37 | 56.84 | 40.3 | |
| ASP | 154 | 61.42 | 10.66 | 2.13 | 59.29 | 52.5 | o |
| ALA | 155 | 0.00 | 0.00 | 0.00 | 0.00 | 0.0 | i |
| PHE | 156 | 0.00 | 0.00 | 0.00 | 0.00 | 0.0 | i |
| TYR | 157 | 47.78 | 32.10 | 0.00 | 47.78 | 24.7 | |
| THR | 158 | 23.97 | 15.34 | 0.00 | 23.97 | 22.6 | |
| LEU | 159 | 0.00 | 0.00 | 0.00 | 0.00 | 0.0 | i |
| TYR | 160 | 0.50 | 0.05 | 0.00 | 0.50 | 0.3 | i |
| ARG | 161 | 94.00 | 53.98 | 0.89 | 93.11 | 47.6 | |
| GLU | 162 | 50.13 | 0.25 | 1.63 | 48.50 | 34.3 | |
| ILE | 163 | 0.04 | 0.04 | 0.00 | 0.04 | 0.0 | i |
| ARG | 164 | 63.05 | 17.05 | 1.89 | 61.16 | 31.3 | |
| GLN | 165 | 84.16 | 24.55 | 0.00 | 84.16 | 58.6 | o |
| TYR | 166 | 48.49 | 28.91 | 2.27 | 46.22 | 23.9 | |
| PHE | 167 | 41.24 | 41.24 | 0.00 | 41.24 | 22.9 | |
| ILE | 168 | 96.61 | 96.07 | 1.64 | 94.98 | 64.5 | o |
| LYS | 169 | 111.21 | 70.26 | 1.52 | 109.69 | 66.7 | o |
| LYS | 170 | 93.75 | 55.90 | 2.08 | 91.67 | 55.7 | o |
| ILE | 171 | 77.96 | 77.96 | 0.03 | 77.92 | 52.9 | o |
| SER | 172 | 43.31 | 32.57 | 1.67 | 41.65 | 53.8 | o |
| LYS | 173 | 135.58 | 97.10 | 3.49 | 132.09 | 80.3 | o |
| GLU | 174 | 91.41 | 37.22 | 8.55 | 82.85 | 58.7 | o |
| GLU | 175 | 135.34 | 43.47 | 37.72 | 97.63 | 69.1 | o |
| LYS | 176 | 166.37 | 106.24 | 26.35 | 140.02 | 85.1 | o |
| THR | 177 | 81.82 | 58.59 | 9.09 | 72.74 | 68.5 | o |
| PRO | 178 | 142.14 | 121.76 | 34.80 | 107.35 | 100.0 | o |
| GLY | 179 | 77.77 | 50.65 | 77.77 | 0.00 | 89.2 | o |
| CYS | 180 | 92.52 | 15.33 | 22.98 | 69.53 | 68.0 | o |
| VAL | 181 | 139.20 | 131.29 | 16.26 | 122.94 | 100.0 | o |
| LYS | 182 | 167.00 | 106.05 | 27.01 | 139.99 | 85.1 | o |
| ILE | 183 | 164.83 | 143.59 | 25.90 | 138.93 | 94.3 | o |
| LYS | 184 | 166.40 | 102.20 | 28.21 | 138.19 | 84.0 | o |
| LYS | 185 | 182.09 | 124.08 | 16.92 | 165.16 | 100.0 | o |

| | | | | | | |
|---|---|---|---|---|---|---|
| CYS 186 | 119.91 | 40.06 | 31.53 | 88.38 | 86.4 | o |
| ILE 187 | 75.54 | 65.40 | 15.64 | 59.90 | 40.7 | |
| ILE 188 | 161.65 | 143.91 | 27.93 | 133.72 | 90.8 | o |
| MET 189 | 223.05 | 165.51 | 52.14 | 170.91 | 100.0 | o |

---------------------------------------------------

POLAR area/energy = 4584.23
APOLAR area/energy = 6327.64
UNKNOW area/energy = 0.00
---------------------------------------------------
Total area/energy = 10911.86
---------------------------------------------------
Number of surface atoms = 911
Number of buried atoms = 630
Number of atoms with ASP=0 = 0

**Supplementary Table 1**: The Solvent Accessible Surface Area (SASA) analysis of pan-ras mRNA cancer vaccine (WO 2022/081764 A1 (PCT/US 2021/054859) using GETAREA tools of FANTOM program.

| Peptide | Allele |
|---|---|
| ADDVGKSAFTIQLIQ | HLA-DPA1*01:03/DPB1*02:01, HLA-DPA1*01:03/DPB1*04:01, HLA-DPA1*02:01/DPB1*01:01, HLA-DPA1*02:01/DPB1*05:01, HLA-DPA1*02:01/DPB1*14:01, HLA-DPA1*03:01/DPB1*04:02, HLA-DQA1*01:01/DQB1*05:01, HLA-DQA1*01:02/DQB1*06:02, HLA-DQA1*03:01/DQB1*03:02, HLA-DQA1*04:01/DQB1*04:02, HLA-DQA1*05:01/DQB1*02:01, HLA-DQA1*05:01/DQB1*03:01, HLA-DRB1*01:01, HLA-DRB1*03:01, HLA-DRB1*04:01, HLA-DRB1*04:05, HLA-DRB1*07:01, HLA-DRB1*08:02, HLA-DRB1*09:01, HLA-DRB1*11:01, HLA-DRB1*12:01, HLA-DRB1*13:02, HLA-DRB1*15:01, HLA-DRB3*01:01, HLA-DRB3*02:02, HLA-DRB4*01:01, HLA-DRB5*01:01 |
| AFTIQLIQNHFVDEY | HLA-DPA1*01:03/DPB1*02:01, HLA-DPA1*01:03/DPB1*04:01, HLA-DPA1*02:01/DPB1*01:01, HLA-DPA1*02:01/DPB1*05:01, HLA-DPA1*02:01/DPB1*14:01, HLA-DPA1*03:01/DPB1*04:02, HLA-DQA1*01:01/DQB1*05:01, HLA-DQA1*01:02/DQB1*06:02, HLA-DQA1*03:01/DQB1*03:02, HLA-DQA1*04:01/DQB1*04:02, HLA-DQA1*05:01/DQB1*02:01, HLA-DQA1*05:01/DQB1*03:01, HLA-DRB1*01:01, HLA-DRB1*03:01, HLA-DRB1*04:01, HLA-DRB1*04:05, HLA-DRB1*07:01, HLA-DRB1*08:02, HLA-DRB1*09:01, HLA-DRB1*11:01, HLA-DRB1*12:01, HLA-DRB1*13:02, HLA-DRB1*15:01, HLA-DRB3*01:01, HLA-DRB3*02:02, HLA-DRB4*01:01, HLA-DRB5*01:01 |
| AFYTLYREIRQYFIK | HLA-DPA1*01:03/DPB1*02:01, HLA-DPA1*01:03/DPB1*04:01, HLA-DPA1*02:01/DPB1*01:01, HLA-DPA1*02:01/DPB1*05:01, HLA-DPA1*02:01/DPB1*14:01, HLA-DPA1*03:01/DPB1*04:02, HLA-DQA1*01:01/DQB1*05:01, HLA-DQA1*01:02/DQB1*06:02, HLA-DQA1*03:01/DQB1*03:02, HLA-DQA1*04:01/DQB1*04:02, HLA-DQA1*05:01/DQB1*02:01, HLA-DQA1*05:01/DQB1*03:01, HLA-DRB1*01:01, |

| | |
|---|---|
| | HLA-DRB1*03:01, HLA-DRB1*04:01, HLA-DRB1*04:05, HLA-DRB1*07:01, HLA-DRB1*08:02, HLA-DRB1*09:01, HLA-DRB1*11:01, HLA-DRB1*12:01, HLA-DRB1*13:02, HLA-DRB1*15:01, HLA-DRB3*01:01, HLA-DRB3*02:02, HLA-DRB4*01:01, HLA-DRB5*01:01 |
| AINNTKSFEDIHHYR | HLA-DPA1*01:03/DPB1*02:01, HLA-DPA1*01:03/DPB1*04:01, HLA-DPA1*02:01/DPB1*01:01, HLA-DPA1*02:01/DPB1*05:01, HLA-DPA1*02:01/DPB1*14:01, HLA-DPA1*03:01/DPB1*04:02, HLA-DQA1*01:01/DQB1*05:01, HLA-DQA1*01:02/DQB1*06:02, HLA-DQA1*03:01/DQB1*03:02, HLA-DQA1*04:01/DQB1*04:02, HLA-DQA1*05:01/DQB1*02:01, HLA-DQA1*05:01/DQB1*03:01, HLA-DRB1*01:01, HLA-DRB1*03:01, HLA-DRB1*04:01, HLA-DRB1*04:05, HLA-DRB1*07:01, HLA-DRB1*08:02, HLA-DRB1*09:01, HLA-DRB1*11:01, HLA-DRB1*12:01, HLA-DRB1*13:02, HLA-DRB1*15:01, HLA-DRB3*01:01, HLA-DRB3*02:02, HLA-DRB4*01:01, HLA-DRB5*01:01 |
| AMRDQYMRTGEGFLC | HLA-DPA1*01:03/DPB1*02:01, HLA-DPA1*01:03/DPB1*04:01, HLA-DPA1*02:01/DPB1*01:01, HLA-DPA1*02:01/DPB1*05:01, HLA-DPA1*02:01/DPB1*14:01, HLA-DPA1*03:01/DPB1*04:02, HLA-DQA1*01:01/DQB1*05:01, HLA-DQA1*01:02/DQB1*06:02, HLA-DQA1*03:01/DQB1*03:02, HLA-DQA1*04:01/DQB1*04:02, HLA-DQA1*05:01/DQB1*02:01, HLA-DQA1*05:01/DQB1*03:01, HLA-DRB1*01:01, HLA-DRB1*03:01, HLA-DRB1*04:01, HLA-DRB1*04:05, HLA-DRB1*07:01, HLA-DRB1*08:02, HLA-DRB1*09:01, HLA-DRB1*11:01, HLA-DRB1*12:01, HLA-DRB1*13:02, HLA-DRB1*15:01, HLA-DRB3*01:01, HLA-DRB3*02:02, HLA-DRB4*01:01, HLA-DRB5*01:01 |
| AQDLARSYGIPFIET | HLA-DPA1*01:03/DPB1*02:01, HLA-DPA1*01:03/DPB1*04:01, HLA-DPA1*02:01/DPB1*01:01, HLA-DPA1*02:01/DPB1*05:01, HLA-DPA1*02:01/DPB1*14:01, HLA-DPA1*03:01/DPB1*04:02, HLA-DQA1*01:01/DQB1*05:01, HLA-DQA1*01:02/DQB1*06:02, HLA-DQA1*03:01/DQB1*03:02, HLA-DQA1*04:01/DQB1*04:02, HLA-DQA1*05:01/DQB1*02:01, HLA-DQA1*05:01/DQB1*03:01, HLA-DRB1*01:01, HLA-DRB1*03:01, HLA-DRB1*04:01, HLA-DRB1*04:05, HLA-DRB1*07:01, HLA-DRB1*08:02, HLA-DRB1*09:01, HLA-DRB1*11:01, HLA-DRB1*12:01, HLA-DRB1*13:02, HLA-DRB1*15:01, HLA-DRB3*01:01, HLA-DRB3*02:02, HLA-DRB4*01:01, HLA-DRB5*01:01 |
| ARSYGIPFIETSTKT | HLA-DPA1*01:03/DPB1*02:01, HLA-DPA1*01:03/DPB1*04:01, HLA-DPA1*02:01/DPB1*01:01, HLA-DPA1*02:01/DPB1*05:01, HLA-DPA1*02:01/DPB1*14:01, HLA-DPA1*03:01/DPB1*04:02, HLA-DQA1*01:01/DQB1*05:01, HLA-DQA1*01:02/DQB1*06:02, HLA-DQA1*03:01/DQB1*03:02, HLA-DQA1*04:01/DQB1*04:02, HLA-DQA1*05:01/DQB1*02:01, HLA-DQA1*05:01/DQB1*03:01, HLA-DRB1*01:01, HLA-DRB1*03:01, HLA-DRB1*04:01, HLA-DRB1*04:05, HLA-DRB1*07:01, HLA-DRB1*08:02, HLA-DRB1*09:01, HLA-DRB1*11:01, HLA-DRB1*12:01, HLA-DRB1*13:02, HLA-DRB1*15:01, HLA-DRB3*01:01, HLA-DRB3*02:02, HLA-DRB4*01:01, HLA-DRB5*01:01 |
| CDLPSRTVDTKQAQD | HLA-DPA1*01:03/DPB1*02:01, HLA-DPA1*01:03/DPB1*04:01, HLA-DPA1*02:01/DPB1*01:01, HLA-DPA1*02:01/DPB1*05:01, HLA-DPA1*02:01/DPB1*14:01, HLA-DPA1*03:01/DPB1*04:02, HLA-DQA1*01:01/DQB1*05:01, HLA-DQA1*01:02/DQB1*06:02, HLA-DQA1*03:01/DQB1*03:02, HLA-DQA1*04:01/DQB1*04:02, HLA-DQA1*05:01/DQB1*02:01, HLA-DQA1*05:01/DQB1*03:01, HLA-DRB1*01:01, HLA-DRB1*03:01, HLA-DRB1*04:01, HLA-DRB1*04:05, HLA-DRB1*07:01, HLA-DRB1*08:02, HLA-DRB1*09:01, HLA-DRB1*11:01, HLA-DRB1*12:01, HLA-DRB1*13:02, HLA-DRB1*15:01, HLA-DRB3*01:01, HLA-DRB3*02:02, HLA-DRB4*01:01, HLA-DRB5*01:01 |
| CLLDILDTTDHEEYS | HLA-DPA1*01:03/DPB1*02:01, HLA-DPA1*01:03/DPB1*04:01, HLA-DPA1*02:01/DPB1*01:01, HLA-DPA1*02:01/DPB1*05:01, HLA-DPA1*02:01/DPB1*14:01, HLA-DPA1*03:01/DPB1*04:02, HLA-DQA1*01:01/DQB1*05:01, HLA-DQA1*01:02/DQB1*06:02, HLA-DQA1*03:01/DQB1*03:02, HLA-DQA1*04:01/DQB1*04:02, HLA-DQA1*05:01/DQB1*02:01, HLA-DQA1*05:01/DQB1*03:01, HLA-DRB1*01:01, |

| | |
|---|---|
| | HLA-DRB1*03:01, HLA-DRB1*04:01, HLA-DRB1*04:05, HLA-DRB1*07:01, HLA-DRB1*08:02, HLA-DRB1*09:01, HLA-DRB1*11:01, HLA-DRB1*12:01, HLA-DRB1*13:02, HLA-DRB1*15:01, HLA-DRB3*01:01, HLA-DRB3*02:02, HLA-DRB4*01:01, HLA-DRB5*01:01 |
| CVFAINNTKSFEDIH | HLA-DPA1*01:03/DPB1*02:01, HLA-DPA1*01:03/DPB1*04:01, HLA-DPA1*02:01/DPB1*01:01, HLA-DPA1*02:01/DPB1*05:01, HLA-DPA1*02:01/DPB1*14:01, HLA-DPA1*03:01/DPB1*04:02, HLA-DQA1*01:01/DQB1*05:01, HLA-DQA1*01:02/DQB1*06:02, HLA-DQA1*03:01/DQB1*03:02, HLA-DQA1*04:01/DQB1*04:02, HLA-DQA1*05:01/DQB1*02:01, HLA-DQA1*05:01/DQB1*03:01, HLA-DRB1*01:01, HLA-DRB1*03:01, HLA-DRB1*04:01, HLA-DRB1*04:05, HLA-DRB1*07:01, HLA-DRB1*08:02, HLA-DRB1*09:01, HLA-DRB1*11:01, HLA-DRB1*12:01, HLA-DRB1*13:02, HLA-DRB1*15:01, HLA-DRB3*01:01, HLA-DRB3*02:02, HLA-DRB4*01:01, HLA-DRB5*01:01 |
| DAFYTLYREIRQYFI | HLA-DPA1*01:03/DPB1*02:01, HLA-DPA1*01:03/DPB1*04:01, HLA-DPA1*02:01/DPB1*01:01, HLA-DPA1*02:01/DPB1*05:01, HLA-DPA1*02:01/DPB1*14:01, HLA-DPA1*03:01/DPB1*04:02, HLA-DQA1*01:01/DQB1*05:01, HLA-DQA1*01:02/DQB1*06:02, HLA-DQA1*03:01/DQB1*03:02, HLA-DQA1*04:01/DQB1*04:02, HLA-DQA1*05:01/DQB1*02:01, HLA-DQA1*05:01/DQB1*03:01, HLA-DRB1*01:01, HLA-DRB1*03:01, HLA-DRB1*04:01, HLA-DRB1*04:05, HLA-DRB1*07:01, HLA-DRB1*08:02, HLA-DRB1*09:01, HLA-DRB1*11:01, HLA-DRB1*12:01, HLA-DRB1*13:02, HLA-DRB1*15:01, HLA-DRB3*01:01, HLA-DRB3*02:02, HLA-DRB4*01:01, HLA-DRB5*01:01 |
| DDVGKSAFTIQLIQN | HLA-DPA1*01:03/DPB1*02:01, HLA-DPA1*01:03/DPB1*04:01, HLA-DPA1*02:01/DPB1*01:01, HLA-DPA1*02:01/DPB1*05:01, HLA-DPA1*02:01/DPB1*14:01, HLA-DPA1*03:01/DPB1*04:02, HLA-DQA1*01:01/DQB1*05:01, HLA-DQA1*01:02/DQB1*06:02, HLA-DQA1*03:01/DQB1*03:02, HLA-DQA1*04:01/DQB1*04:02, HLA-DQA1*05:01/DQB1*02:01, HLA-DQA1*05:01/DQB1*03:01, HLA-DRB1*01:01, HLA-DRB1*03:01, HLA-DRB1*04:01, HLA-DRB1*04:05, HLA-DRB1*07:01, HLA-DRB1*08:02, HLA-DRB1*09:01, HLA-DRB1*11:01, HLA-DRB1*12:01, HLA-DRB1*13:02, HLA-DRB1*15:01, HLA-DRB3*01:01, HLA-DRB3*02:02, HLA-DRB4*01:01, HLA-DRB5*01:01 |
| DEYDPTIEDSYRKQV | HLA-DPA1*01:03/DPB1*02:01, HLA-DPA1*01:03/DPB1*04:01, HLA-DPA1*02:01/DPB1*01:01, HLA-DPA1*02:01/DPB1*05:01, HLA-DPA1*02:01/DPB1*14:01, HLA-DPA1*03:01/DPB1*04:02, HLA-DQA1*01:01/DQB1*05:01, HLA-DQA1*01:02/DQB1*06:02, HLA-DQA1*03:01/DQB1*03:02, HLA-DQA1*04:01/DQB1*04:02, HLA-DQA1*05:01/DQB1*02:01, HLA-DQA1*05:01/DQB1*03:01, HLA-DRB1*01:01, HLA-DRB1*03:01, HLA-DRB1*04:01, HLA-DRB1*04:05, HLA-DRB1*07:01, HLA-DRB1*08:02, HLA-DRB1*09:01, HLA-DRB1*11:01, HLA-DRB1*12:01, HLA-DRB1*13:02, HLA-DRB1*15:01, HLA-DRB3*01:01, HLA-DRB3*02:02, HLA-DRB4*01:01, HLA-DRB5*01:01 |
| DHEEYSAMRDQYMRT | HLA-DPA1*01:03/DPB1*02:01, HLA-DPA1*01:03/DPB1*04:01, HLA-DPA1*02:01/DPB1*01:01, HLA-DPA1*02:01/DPB1*05:01, HLA-DPA1*02:01/DPB1*14:01, HLA-DPA1*03:01/DPB1*04:02, HLA-DQA1*01:01/DQB1*05:01, HLA-DQA1*01:02/DQB1*06:02, HLA-DQA1*03:01/DQB1*03:02, HLA-DQA1*04:01/DQB1*04:02, HLA-DQA1*05:01/DQB1*02:01, HLA-DQA1*05:01/DQB1*03:01, HLA-DRB1*01:01, HLA-DRB1*03:01, HLA-DRB1*04:01, HLA-DRB1*04:05, HLA-DRB1*07:01, HLA-DRB1*08:02, HLA-DRB1*09:01, HLA-DRB1*11:01, HLA-DRB1*12:01, HLA-DRB1*13:02, HLA-DRB1*15:01, HLA-DRB3*01:01, HLA-DRB3*02:02, HLA-DRB4*01:01, HLA-DRB5*01:01 |
| DILDTTDHEEYSAMR | HLA-DPA1*01:03/DPB1*02:01, HLA-DPA1*01:03/DPB1*04:01, HLA-DPA1*02:01/DPB1*01:01, HLA-DPA1*02:01/DPB1*05:01, HLA-DPA1*02:01/DPB1*14:01, HLA-DPA1*03:01/DPB1*04:02, HLA-DQA1*01:01/DQB1*05:01, HLA-DQA1*01:02/DQB1*06:02, HLA-DQA1*03:01/DQB1*03:02, HLA-DQA1*04:01/DQB1*04:02, HLA-DQA1*05:01/DQB1*02:01, HLA-DQA1*05:01/DQB1*03:01, HLA-DRB1*01:01, |

| | |
|---|---|
| | HLA-DRB1*03:01, HLA-DRB1*04:01, HLA-DRB1*04:05, HLA-DRB1*07:01, HLA-DRB1*08:02, HLA-DRB1*09:01, HLA-DRB1*11:01, HLA-DRB1*12:01, HLA-DRB1*13:02, HLA-DRB1*15:01, HLA-DRB3*01:01, HLA-DRB3*02:02, HLA-DRB4*01:01, HLA-DRB5*01:01 |
| DLARSYGIPFIETST | HLA-DPA1*01:03/DPB1*02:01, HLA-DPA1*01:03/DPB1*04:01, HLA-DPA1*02:01/DPB1*01:01, HLA-DPA1*02:01/DPB1*05:01, HLA-DPA1*02:01/DPB1*14:01, HLA-DPA1*03:01/DPB1*04:02, HLA-DQA1*01:01/DQB1*05:01, HLA-DQA1*01:02/DQB1*06:02, HLA-DQA1*03:01/DQB1*03:02, HLA-DQA1*04:01/DQB1*04:02, HLA-DQA1*05:01/DQB1*02:01, HLA-DQA1*05:01/DQB1*03:01, HLA-DRB1*01:01, HLA-DRB1*03:01, HLA-DRB1*04:01, HLA-DRB1*04:05, HLA-DRB1*07:01, HLA-DRB1*08:02, HLA-DRB1*09:01, HLA-DRB1*11:01, HLA-DRB1*12:01, HLA-DRB1*13:02, HLA-DRB1*15:01, HLA-DRB3*01:01, HLA-DRB3*02:02, HLA-DRB4*01:01, HLA-DRB5*01:01 |
| DLPSRTVDTKQAQDL | HLA-DPA1*01:03/DPB1*02:01, HLA-DPA1*01:03/DPB1*04:01, HLA-DPA1*02:01/DPB1*01:01, HLA-DPA1*02:01/DPB1*05:01, HLA-DPA1*02:01/DPB1*14:01, HLA-DPA1*03:01/DPB1*04:02, HLA-DQA1*01:01/DQB1*05:01, HLA-DQA1*01:02/DQB1*06:02, HLA-DQA1*03:01/DQB1*03:02, HLA-DQA1*04:01/DQB1*04:02, HLA-DQA1*05:01/DQB1*02:01, HLA-DQA1*05:01/DQB1*03:01, HLA-DRB1*01:01, HLA-DRB1*03:01, HLA-DRB1*04:01, HLA-DRB1*04:05, HLA-DRB1*07:01, HLA-DRB1*08:02, HLA-DRB1*09:01, HLA-DRB1*11:01, HLA-DRB1*12:01, HLA-DRB1*13:02, HLA-DRB1*15:01, HLA-DRB3*01:01, HLA-DRB3*02:02, HLA-DRB4*01:01, HLA-DRB5*01:01 |
| DPTIEDSYRKQVVID | HLA-DPA1*01:03/DPB1*02:01, HLA-DPA1*01:03/DPB1*04:01, HLA-DPA1*02:01/DPB1*01:01, HLA-DPA1*02:01/DPB1*05:01, HLA-DPA1*02:01/DPB1*14:01, HLA-DPA1*03:01/DPB1*04:02, HLA-DQA1*01:01/DQB1*05:01, HLA-DQA1*01:02/DQB1*06:02, HLA-DQA1*03:01/DQB1*03:02, HLA-DQA1*04:01/DQB1*04:02, HLA-DQA1*05:01/DQB1*02:01, HLA-DQA1*05:01/DQB1*03:01, HLA-DRB1*01:01, HLA-DRB1*03:01, HLA-DRB1*04:01, HLA-DRB1*04:05, HLA-DRB1*07:01, HLA-DRB1*08:02, HLA-DRB1*09:01, HLA-DRB1*11:01, HLA-DRB1*12:01, HLA-DRB1*13:02, HLA-DRB1*15:01, HLA-DRB3*01:01, HLA-DRB3*02:02, HLA-DRB4*01:01, HLA-DRB5*01:01 |
| DQYMRTGEGFLCVFA | HLA-DPA1*01:03/DPB1*02:01, HLA-DPA1*01:03/DPB1*04:01, HLA-DPA1*02:01/DPB1*01:01, HLA-DPA1*02:01/DPB1*05:01, HLA-DPA1*02:01/DPB1*14:01, HLA-DPA1*03:01/DPB1*04:02, HLA-DQA1*01:01/DQB1*05:01, HLA-DQA1*01:02/DQB1*06:02, HLA-DQA1*03:01/DQB1*03:02, HLA-DQA1*04:01/DQB1*04:02, HLA-DQA1*05:01/DQB1*02:01, HLA-DQA1*05:01/DQB1*03:01, HLA-DRB1*01:01, HLA-DRB1*03:01, HLA-DRB1*04:01, HLA-DRB1*04:05, HLA-DRB1*07:01, HLA-DRB1*08:02, HLA-DRB1*09:01, HLA-DRB1*11:01, HLA-DRB1*12:01, HLA-DRB1*13:02, HLA-DRB1*15:01, HLA-DRB3*01:01, HLA-DRB3*02:02, HLA-DRB4*01:01, HLA-DRB5*01:01 |
| DSEDVPMVLVGNNCD | HLA-DPA1*01:03/DPB1*02:01, HLA-DPA1*01:03/DPB1*04:01, HLA-DPA1*02:01/DPB1*01:01, HLA-DPA1*02:01/DPB1*05:01, HLA-DPA1*02:01/DPB1*14:01, HLA-DPA1*03:01/DPB1*04:02, HLA-DQA1*01:01/DQB1*05:01, HLA-DQA1*01:02/DQB1*06:02, HLA-DQA1*03:01/DQB1*03:02, HLA-DQA1*04:01/DQB1*04:02, HLA-DQA1*05:01/DQB1*02:01, HLA-DQA1*05:01/DQB1*03:01, HLA-DRB1*01:01, HLA-DRB1*03:01, HLA-DRB1*04:01, HLA-DRB1*04:05, HLA-DRB1*07:01, HLA-DRB1*08:02, HLA-DRB1*09:01, HLA-DRB1*11:01, HLA-DRB1*12:01, HLA-DRB1*13:02, HLA-DRB1*15:01, HLA-DRB3*01:01, HLA-DRB3*02:02, HLA-DRB4*01:01, HLA-DRB5*01:01 |
| DTKQAQDLARSYGIP | HLA-DPA1*01:03/DPB1*02:01, HLA-DPA1*01:03/DPB1*04:01, HLA-DPA1*02:01/DPB1*01:01, HLA-DPA1*02:01/DPB1*05:01, HLA-DPA1*02:01/DPB1*14:01, HLA-DPA1*03:01/DPB1*04:02, HLA-DQA1*01:01/DQB1*05:01, HLA-DQA1*01:02/DQB1*06:02, HLA-DQA1*03:01/DQB1*03:02, HLA-DQA1*04:01/DQB1*04:02, HLA-DQA1*05:01/DQB1*02:01, HLA-DQA1*05:01/DQB1*03:01, HLA-DRB1*01:01, |

| | |
|---|---|
| | HLA-DRB1*03:01, HLA-DRB1*04:01, HLA-DRB1*04:05, HLA-DRB1*07:01, HLA-DRB1*08:02, HLA-DRB1*09:01, HLA-DRB1*11:01, HLA-DRB1*12:01, HLA-DRB1*13:02, HLA-DRB1*15:01, HLA-DRB3*01:01, HLA-DRB3*02:02, HLA-DRB4*01:01, HLA-DRB5*01:01 |
| DTTDHEEYSAMRDQY | HLA-DPA1*01:03/DPB1*02:01, HLA-DPA1*01:03/DPB1*04:01, HLA-DPA1*02:01/DPB1*01:01, HLA-DPA1*02:01/DPB1*05:01, HLA-DPA1*02:01/DPB1*14:01, HLA-DPA1*03:01/DPB1*04:02, HLA-DQA1*01:01/DQB1*05:01, HLA-DQA1*01:02/DQB1*06:02, HLA-DQA1*03:01/DQB1*03:02, HLA-DQA1*04:01/DQB1*04:02, HLA-DQA1*05:01/DQB1*02:01, HLA-DQA1*05:01/DQB1*03:01, HLA-DRB1*01:01, HLA-DRB1*03:01, HLA-DRB1*04:01, HLA-DRB1*04:05, HLA-DRB1*07:01, HLA-DRB1*08:02, HLA-DRB1*09:01, HLA-DRB1*11:01, HLA-DRB1*12:01, HLA-DRB1*13:02, HLA-DRB1*15:01, HLA-DRB3*01:01, HLA-DRB3*02:02, HLA-DRB4*01:01, HLA-DRB5*01:01 |
| DVGKSAFTIQLIQNH | HLA-DPA1*01:03/DPB1*02:01, HLA-DPA1*01:03/DPB1*04:01, HLA-DPA1*02:01/DPB1*01:01, HLA-DPA1*02:01/DPB1*05:01, HLA-DPA1*02:01/DPB1*14:01, HLA-DPA1*03:01/DPB1*04:02, HLA-DQA1*01:01/DQB1*05:01, HLA-DQA1*01:02/DQB1*06:02, HLA-DQA1*03:01/DQB1*03:02, HLA-DQA1*04:01/DQB1*04:02, HLA-DQA1*05:01/DQB1*02:01, HLA-DQA1*05:01/DQB1*03:01, HLA-DRB1*01:01, HLA-DRB1*03:01, HLA-DRB1*04:01, HLA-DRB1*04:05, HLA-DRB1*07:01, HLA-DRB1*08:02, HLA-DRB1*09:01, HLA-DRB1*11:01, HLA-DRB1*12:01, HLA-DRB1*13:02, HLA-DRB1*15:01, HLA-DRB3*01:01, HLA-DRB3*02:02, HLA-DRB4*01:01, HLA-DRB5*01:01 |
| DVPMVLVGNNCDLPS | HLA-DPA1*01:03/DPB1*02:01, HLA-DPA1*01:03/DPB1*04:01, HLA-DPA1*02:01/DPB1*01:01, HLA-DPA1*02:01/DPB1*05:01, HLA-DPA1*02:01/DPB1*14:01, HLA-DPA1*03:01/DPB1*04:02, HLA-DQA1*01:01/DQB1*05:01, HLA-DQA1*01:02/DQB1*06:02, HLA-DQA1*03:01/DQB1*03:02, HLA-DQA1*04:01/DQB1*04:02, HLA-DQA1*05:01/DQB1*02:01, HLA-DQA1*05:01/DQB1*03:01, HLA-DRB1*01:01, HLA-DRB1*03:01, HLA-DRB1*04:01, HLA-DRB1*04:05, HLA-DRB1*07:01, HLA-DRB1*08:02, HLA-DRB1*09:01, HLA-DRB1*11:01, HLA-DRB1*12:01, HLA-DRB1*13:02, HLA-DRB1*15:01, HLA-DRB3*01:01, HLA-DRB3*02:02, HLA-DRB4*01:01, HLA-DRB5*01:01 |
| EDAFYTLYREIRQYF | HLA-DPA1*01:03/DPB1*02:01, HLA-DPA1*01:03/DPB1*04:01, HLA-DPA1*02:01/DPB1*01:01, HLA-DPA1*02:01/DPB1*05:01, HLA-DPA1*02:01/DPB1*14:01, HLA-DPA1*03:01/DPB1*04:02, HLA-DQA1*01:01/DQB1*05:01, HLA-DQA1*01:02/DQB1*06:02, HLA-DQA1*03:01/DQB1*03:02, HLA-DQA1*04:01/DQB1*04:02, HLA-DQA1*05:01/DQB1*02:01, HLA-DQA1*05:01/DQB1*03:01, HLA-DRB1*01:01, HLA-DRB1*03:01, HLA-DRB1*04:01, HLA-DRB1*04:05, HLA-DRB1*07:01, HLA-DRB1*08:02, HLA-DRB1*09:01, HLA-DRB1*11:01, HLA-DRB1*12:01, HLA-DRB1*13:02, HLA-DRB1*15:01, HLA-DRB3*01:01, HLA-DRB3*02:02, HLA-DRB4*01:01, HLA-DRB5*01:01 |
| EDVPMVLVGNNCDLP | HLA-DPA1*01:03/DPB1*02:01, HLA-DPA1*01:03/DPB1*04:01, HLA-DPA1*02:01/DPB1*01:01, HLA-DPA1*02:01/DPB1*05:01, HLA-DPA1*02:01/DPB1*14:01, HLA-DPA1*03:01/DPB1*04:02, HLA-DQA1*01:01/DQB1*05:01, HLA-DQA1*01:02/DQB1*06:02, HLA-DQA1*03:01/DQB1*03:02, HLA-DQA1*04:01/DQB1*04:02, HLA-DQA1*05:01/DQB1*02:01, HLA-DQA1*05:01/DQB1*03:01, HLA-DRB1*01:01, HLA-DRB1*03:01, HLA-DRB1*04:01, HLA-DRB1*04:05, HLA-DRB1*07:01, HLA-DRB1*08:02, HLA-DRB1*09:01, HLA-DRB1*11:01, HLA-DRB1*12:01, HLA-DRB1*13:02, HLA-DRB1*15:01, HLA-DRB3*01:01, HLA-DRB3*02:02, HLA-DRB4*01:01, HLA-DRB5*01:01 |
| EEKTPGCVKIKKCII | HLA-DPA1*01:03/DPB1*02:01, HLA-DPA1*01:03/DPB1*04:01, HLA-DPA1*02:01/DPB1*01:01, HLA-DPA1*02:01/DPB1*05:01, HLA-DPA1*02:01/DPB1*14:01, HLA-DPA1*03:01/DPB1*04:02, HLA-DQA1*01:01/DQB1*05:01, HLA-DQA1*01:02/DQB1*06:02, HLA-DQA1*03:01/DQB1*03:02, HLA-DQA1*04:01/DQB1*04:02, HLA-DQA1*05:01/DQB1*02:01, HLA-DQA1*05:01/DQB1*03:01, HLA-DRB1*01:01, |

| | |
|---|---|
| | HLA-DRB1*03:01, HLA-DRB1*04:01, HLA-DRB1*04:05, HLA-DRB1*07:01, HLA-DRB1*08:02, HLA-DRB1*09:01, HLA-DRB1*11:01, HLA-DRB1*12:01, HLA-DRB1*13:02, HLA-DRB1*15:01, HLA-DRB3*01:01, HLA-DRB3*02:02, HLA-DRB4*01:01, HLA-DRB5*01:01 |
| EEYSAMRDQYMRTGE | HLA-DPA1*01:03/DPB1*02:01, HLA-DPA1*01:03/DPB1*04:01, HLA-DPA1*02:01/DPB1*01:01, HLA-DPA1*02:01/DPB1*05:01, HLA-DPA1*02:01/DPB1*14:01, HLA-DPA1*03:01/DPB1*04:02, HLA-DQA1*01:01/DQB1*05:01, HLA-DQA1*01:02/DQB1*06:02, HLA-DQA1*03:01/DQB1*03:02, HLA-DQA1*04:01/DQB1*04:02, HLA-DQA1*05:01/DQB1*02:01, HLA-DQA1*05:01/DQB1*03:01, HLA-DRB1*01:01, HLA-DRB1*03:01, HLA-DRB1*04:01, HLA-DRB1*04:05, HLA-DRB1*07:01, HLA-DRB1*08:02, HLA-DRB1*09:01, HLA-DRB1*11:01, HLA-DRB1*12:01, HLA-DRB1*13:02, HLA-DRB1*15:01, HLA-DRB3*01:01, HLA-DRB3*02:02, HLA-DRB4*01:01, HLA-DRB5*01:01 |
| EGFLCVFAINNTKSF | HLA-DPA1*01:03/DPB1*02:01, HLA-DPA1*01:03/DPB1*04:01, HLA-DPA1*02:01/DPB1*01:01, HLA-DPA1*02:01/DPB1*05:01, HLA-DPA1*02:01/DPB1*14:01, HLA-DPA1*03:01/DPB1*04:02, HLA-DQA1*01:01/DQB1*05:01, HLA-DQA1*01:02/DQB1*06:02, HLA-DQA1*03:01/DQB1*03:02, HLA-DQA1*04:01/DQB1*04:02, HLA-DQA1*05:01/DQB1*02:01, HLA-DQA1*05:01/DQB1*03:01, HLA-DRB1*01:01, HLA-DRB1*03:01, HLA-DRB1*04:01, HLA-DRB1*04:05, HLA-DRB1*07:01, HLA-DRB1*08:02, HLA-DRB1*09:01, HLA-DRB1*11:01, HLA-DRB1*12:01, HLA-DRB1*13:02, HLA-DRB1*15:01, HLA-DRB3*01:01, HLA-DRB3*02:02, HLA-DRB4*01:01, HLA-DRB5*01:01 |
| EIRQYFIKKISKEEK | HLA-DPA1*01:03/DPB1*02:01, HLA-DPA1*01:03/DPB1*04:01, HLA-DPA1*02:01/DPB1*01:01, HLA-DPA1*02:01/DPB1*05:01, HLA-DPA1*02:01/DPB1*14:01, HLA-DPA1*03:01/DPB1*04:02, HLA-DQA1*01:01/DQB1*05:01, HLA-DQA1*01:02/DQB1*06:02, HLA-DQA1*03:01/DQB1*03:02, HLA-DQA1*04:01/DQB1*04:02, HLA-DQA1*05:01/DQB1*02:01, HLA-DQA1*05:01/DQB1*03:01, HLA-DRB1*01:01, HLA-DRB1*03:01, HLA-DRB1*04:01, HLA-DRB1*04:05, HLA-DRB1*07:01, HLA-DRB1*08:02, HLA-DRB1*09:01, HLA-DRB1*11:01, HLA-DRB1*12:01, HLA-DRB1*13:02, HLA-DRB1*15:01, HLA-DRB3*01:01, HLA-DRB3*02:02, HLA-DRB4*01:01, HLA-DRB5*01:01 |
| EKTPGCVKIKKCIIM | HLA-DPA1*01:03/DPB1*02:01, HLA-DPA1*01:03/DPB1*04:01, HLA-DPA1*02:01/DPB1*01:01, HLA-DPA1*02:01/DPB1*05:01, HLA-DPA1*02:01/DPB1*14:01, HLA-DPA1*03:01/DPB1*04:02, HLA-DQA1*01:01/DQB1*05:01, HLA-DQA1*01:02/DQB1*06:02, HLA-DQA1*03:01/DQB1*03:02, HLA-DQA1*04:01/DQB1*04:02, HLA-DQA1*05:01/DQB1*02:01, HLA-DQA1*05:01/DQB1*03:01, HLA-DRB1*01:01, HLA-DRB1*03:01, HLA-DRB1*04:01, HLA-DRB1*04:05, HLA-DRB1*07:01, HLA-DRB1*08:02, HLA-DRB1*09:01, HLA-DRB1*11:01, HLA-DRB1*12:01, HLA-DRB1*13:02, HLA-DRB1*15:01, HLA-DRB3*01:01, HLA-DRB3*02:02, HLA-DRB4*01:01, HLA-DRB5*01:01 |
| EYDPTIEDSYRKQVV | HLA-DPA1*01:03/DPB1*02:01, HLA-DPA1*01:03/DPB1*04:01, HLA-DPA1*02:01/DPB1*01:01, HLA-DPA1*02:01/DPB1*05:01, HLA-DPA1*02:01/DPB1*14:01, HLA-DPA1*03:01/DPB1*04:02, HLA-DQA1*01:01/DQB1*05:01, HLA-DQA1*01:02/DQB1*06:02, HLA-DQA1*03:01/DQB1*03:02, HLA-DQA1*04:01/DQB1*04:02, HLA-DQA1*05:01/DQB1*02:01, HLA-DQA1*05:01/DQB1*03:01, HLA-DRB1*01:01, HLA-DRB1*03:01, HLA-DRB1*04:01, HLA-DRB1*04:05, HLA-DRB1*07:01, HLA-DRB1*08:02, HLA-DRB1*09:01, HLA-DRB1*11:01, HLA-DRB1*12:01, HLA-DRB1*13:02, HLA-DRB1*15:01, HLA-DRB3*01:01, HLA-DRB3*02:02, HLA-DRB4*01:01, HLA-DRB5*01:01 |
| EYKLVVVGADDVGKS | HLA-DPA1*01:03/DPB1*02:01, HLA-DPA1*01:03/DPB1*04:01, HLA-DPA1*02:01/DPB1*01:01, HLA-DPA1*02:01/DPB1*05:01, HLA-DPA1*02:01/DPB1*14:01, HLA-DPA1*03:01/DPB1*04:02, HLA-DQA1*01:01/DQB1*05:01, HLA-DQA1*01:02/DQB1*06:02, HLA-DQA1*03:01/DQB1*03:02, HLA-DQA1*04:01/DQB1*04:02, HLA-DQA1*05:01/DQB1*02:01, HLA-DQA1*05:01/DQB1*03:01, HLA-DRB1*01:01, |

| | |
|---|---|
| | HLA-DRB1*03:01, HLA-DRB1*04:01, HLA-DRB1*04:05, HLA-DRB1*07:01, HLA-DRB1*08:02, HLA-DRB1*09:01, HLA-DRB1*11:01, HLA-DRB1*12:01, HLA-DRB1*13:02, HLA-DRB1*15:01, HLA-DRB3*01:01, HLA-DRB3*02:02, HLA-DRB4*01:01, HLA-DRB5*01:01 |
| EYSAMRDQYMRTGEG | HLA-DPA1*01:03/DPB1*02:01, HLA-DPA1*01:03/DPB1*04:01, HLA-DPA1*02:01/DPB1*01:01, HLA-DPA1*02:01/DPB1*05:01, HLA-DPA1*02:01/DPB1*14:01, HLA-DPA1*03:01/DPB1*04:02, HLA-DQA1*01:01/DQB1*05:01, HLA-DQA1*01:02/DQB1*06:02, HLA-DQA1*03:01/DQB1*03:02, HLA-DQA1*04:01/DQB1*04:02, HLA-DQA1*05:01/DQB1*02:01, HLA-DQA1*05:01/DQB1*03:01, HLA-DRB1*01:01, HLA-DRB1*03:01, HLA-DRB1*04:01, HLA-DRB1*04:05, HLA-DRB1*07:01, HLA-DRB1*08:02, HLA-DRB1*09:01, HLA-DRB1*11:01, HLA-DRB1*12:01, HLA-DRB1*13:02, HLA-DRB1*15:01, HLA-DRB3*01:01, HLA-DRB3*02:02, HLA-DRB4*01:01, HLA-DRB5*01:01 |
| FAINNTKSFEDIHHY | HLA-DPA1*01:03/DPB1*02:01, HLA-DPA1*01:03/DPB1*04:01, HLA-DPA1*02:01/DPB1*01:01, HLA-DPA1*02:01/DPB1*05:01, HLA-DPA1*02:01/DPB1*14:01, HLA-DPA1*03:01/DPB1*04:02, HLA-DQA1*01:01/DQB1*05:01, HLA-DQA1*01:02/DQB1*06:02, HLA-DQA1*03:01/DQB1*03:02, HLA-DQA1*04:01/DQB1*04:02, HLA-DQA1*05:01/DQB1*02:01, HLA-DQA1*05:01/DQB1*03:01, HLA-DRB1*01:01, HLA-DRB1*03:01, HLA-DRB1*04:01, HLA-DRB1*04:05, HLA-DRB1*07:01, HLA-DRB1*08:02, HLA-DRB1*09:01, HLA-DRB1*11:01, HLA-DRB1*12:01, HLA-DRB1*13:02, HLA-DRB1*15:01, HLA-DRB3*01:01, HLA-DRB3*02:02, HLA-DRB4*01:01, HLA-DRB5*01:01 |
| FIKKISKEEKTPGCV | HLA-DPA1*01:03/DPB1*02:01, HLA-DPA1*01:03/DPB1*04:01, HLA-DPA1*02:01/DPB1*01:01, HLA-DPA1*02:01/DPB1*05:01, HLA-DPA1*02:01/DPB1*14:01, HLA-DPA1*03:01/DPB1*04:02, HLA-DQA1*01:01/DQB1*05:01, HLA-DQA1*01:02/DQB1*06:02, HLA-DQA1*03:01/DQB1*03:02, HLA-DQA1*04:01/DQB1*04:02, HLA-DQA1*05:01/DQB1*02:01, HLA-DQA1*05:01/DQB1*03:01, HLA-DRB1*01:01, HLA-DRB1*03:01, HLA-DRB1*04:01, HLA-DRB1*04:05, HLA-DRB1*07:01, HLA-DRB1*08:02, HLA-DRB1*09:01, HLA-DRB1*11:01, HLA-DRB1*12:01, HLA-DRB1*13:02, HLA-DRB1*15:01, HLA-DRB3*01:01, HLA-DRB3*02:02, HLA-DRB4*01:01, HLA-DRB5*01:01 |
| FLCVFAINNTKSFED | HLA-DPA1*01:03/DPB1*02:01, HLA-DPA1*01:03/DPB1*04:01, HLA-DPA1*02:01/DPB1*01:01, HLA-DPA1*02:01/DPB1*05:01, HLA-DPA1*02:01/DPB1*14:01, HLA-DPA1*03:01/DPB1*04:02, HLA-DQA1*01:01/DQB1*05:01, HLA-DQA1*01:02/DQB1*06:02, HLA-DQA1*03:01/DQB1*03:02, HLA-DQA1*04:01/DQB1*04:02, HLA-DQA1*05:01/DQB1*02:01, HLA-DQA1*05:01/DQB1*03:01, HLA-DRB1*01:01, HLA-DRB1*03:01, HLA-DRB1*04:01, HLA-DRB1*04:05, HLA-DRB1*07:01, HLA-DRB1*08:02, HLA-DRB1*09:01, HLA-DRB1*11:01, HLA-DRB1*12:01, HLA-DRB1*13:02, HLA-DRB1*15:01, HLA-DRB3*01:01, HLA-DRB3*02:02, HLA-DRB4*01:01, HLA-DRB5*01:01 |
| FTIQLIQNHFVDEYD | HLA-DPA1*01:03/DPB1*02:01, HLA-DPA1*01:03/DPB1*04:01, HLA-DPA1*02:01/DPB1*01:01, HLA-DPA1*02:01/DPB1*05:01, HLA-DPA1*02:01/DPB1*14:01, HLA-DPA1*03:01/DPB1*04:02, HLA-DQA1*01:01/DQB1*05:01, HLA-DQA1*01:02/DQB1*06:02, HLA-DQA1*03:01/DQB1*03:02, HLA-DQA1*04:01/DQB1*04:02, HLA-DQA1*05:01/DQB1*02:01, HLA-DQA1*05:01/DQB1*03:01, HLA-DRB1*01:01, HLA-DRB1*03:01, HLA-DRB1*04:01, HLA-DRB1*04:05, HLA-DRB1*07:01, HLA-DRB1*08:02, HLA-DRB1*09:01, HLA-DRB1*11:01, HLA-DRB1*12:01, HLA-DRB1*13:02, HLA-DRB1*15:01, HLA-DRB3*01:01, HLA-DRB3*02:02, HLA-DRB4*01:01, HLA-DRB5*01:01 |
| FVDEYDPTIEDSYRK | HLA-DPA1*01:03/DPB1*02:01, HLA-DPA1*01:03/DPB1*04:01, HLA-DPA1*02:01/DPB1*01:01, HLA-DPA1*02:01/DPB1*05:01, HLA-DPA1*02:01/DPB1*14:01, HLA-DPA1*03:01/DPB1*04:02, HLA-DQA1*01:01/DQB1*05:01, HLA-DQA1*01:02/DQB1*06:02, HLA-DQA1*03:01/DQB1*03:02, HLA-DQA1*04:01/DQB1*04:02, HLA-DQA1*05:01/DQB1*02:01, HLA-DQA1*05:01/DQB1*03:01, HLA-DRB1*01:01, |

| | |
|---|---|
| | HLA-DRB1*03:01, HLA-DRB1*04:01, HLA-DRB1*04:05, HLA-DRB1*07:01, HLA-DRB1*08:02, HLA-DRB1*09:01, HLA-DRB1*11:01, HLA-DRB1*12:01, HLA-DRB1*13:02, HLA-DRB1*15:01, HLA-DRB3*01:01, HLA-DRB3*02:02, HLA-DRB4*01:01, HLA-DRB5*01:01 |
| FYTLYREIRQYFIKK | HLA-DPA1*01:03/DPB1*02:01, HLA-DPA1*01:03/DPB1*04:01, HLA-DPA1*02:01/DPB1*01:01, HLA-DPA1*02:01/DPB1*05:01, HLA-DPA1*02:01/DPB1*14:01, HLA-DPA1*03:01/DPB1*04:02, HLA-DQA1*01:01/DQB1*05:01, HLA-DQA1*01:02/DQB1*06:02, HLA-DQA1*03:01/DQB1*03:02, HLA-DQA1*04:01/DQB1*04:02, HLA-DQA1*05:01/DQB1*02:01, HLA-DQA1*05:01/DQB1*03:01, HLA-DRB1*01:01, HLA-DRB1*03:01, HLA-DRB1*04:01, HLA-DRB1*04:05, HLA-DRB1*07:01, HLA-DRB1*08:02, HLA-DRB1*09:01, HLA-DRB1*11:01, HLA-DRB1*12:01, HLA-DRB1*13:02, HLA-DRB1*15:01, HLA-DRB3*01:01, HLA-DRB3*02:02, HLA-DRB4*01:01, HLA-DRB5*01:01 |
| GADDVGKSAFTIQLI | HLA-DPA1*01:03/DPB1*02:01, HLA-DPA1*01:03/DPB1*04:01, HLA-DPA1*02:01/DPB1*01:01, HLA-DPA1*02:01/DPB1*05:01, HLA-DPA1*02:01/DPB1*14:01, HLA-DPA1*03:01/DPB1*04:02, HLA-DQA1*01:01/DQB1*05:01, HLA-DQA1*01:02/DQB1*06:02, HLA-DQA1*03:01/DQB1*03:02, HLA-DQA1*04:01/DQB1*04:02, HLA-DQA1*05:01/DQB1*02:01, HLA-DQA1*05:01/DQB1*03:01, HLA-DRB1*01:01, HLA-DRB1*03:01, HLA-DRB1*04:01, HLA-DRB1*04:05, HLA-DRB1*07:01, HLA-DRB1*08:02, HLA-DRB1*09:01, HLA-DRB1*11:01, HLA-DRB1*12:01, HLA-DRB1*13:02, HLA-DRB1*15:01, HLA-DRB3*01:01, HLA-DRB3*02:02, HLA-DRB4*01:01, HLA-DRB5*01:01 |
| GEGFLCVFAINNTKS | HLA-DPA1*01:03/DPB1*02:01, HLA-DPA1*01:03/DPB1*04:01, HLA-DPA1*02:01/DPB1*01:01, HLA-DPA1*02:01/DPB1*05:01, HLA-DPA1*02:01/DPB1*14:01, HLA-DPA1*03:01/DPB1*04:02, HLA-DQA1*01:01/DQB1*05:01, HLA-DQA1*01:02/DQB1*06:02, HLA-DQA1*03:01/DQB1*03:02, HLA-DQA1*04:01/DQB1*04:02, HLA-DQA1*05:01/DQB1*02:01, HLA-DQA1*05:01/DQB1*03:01, HLA-DRB1*01:01, HLA-DRB1*03:01, HLA-DRB1*04:01, HLA-DRB1*04:05, HLA-DRB1*07:01, HLA-DRB1*08:02, HLA-DRB1*09:01, HLA-DRB1*11:01, HLA-DRB1*12:01, HLA-DRB1*13:02, HLA-DRB1*15:01, HLA-DRB3*01:01, HLA-DRB3*02:02, HLA-DRB4*01:01, HLA-DRB5*01:01 |
| GFLCVFAINNTKSFE | HLA-DPA1*01:03/DPB1*02:01, HLA-DPA1*01:03/DPB1*04:01, HLA-DPA1*02:01/DPB1*01:01, HLA-DPA1*02:01/DPB1*05:01, HLA-DPA1*02:01/DPB1*14:01, HLA-DPA1*03:01/DPB1*04:02, HLA-DQA1*01:01/DQB1*05:01, HLA-DQA1*01:02/DQB1*06:02, HLA-DQA1*03:01/DQB1*03:02, HLA-DQA1*04:01/DQB1*04:02, HLA-DQA1*05:01/DQB1*02:01, HLA-DQA1*05:01/DQB1*03:01, HLA-DRB1*01:01, HLA-DRB1*03:01, HLA-DRB1*04:01, HLA-DRB1*04:05, HLA-DRB1*07:01, HLA-DRB1*08:02, HLA-DRB1*09:01, HLA-DRB1*11:01, HLA-DRB1*12:01, HLA-DRB1*13:02, HLA-DRB1*15:01, HLA-DRB3*01:01, HLA-DRB3*02:02, HLA-DRB4*01:01, HLA-DRB5*01:01 |
| GKSAFTIQLIQNHFV | HLA-DPA1*01:03/DPB1*02:01, HLA-DPA1*01:03/DPB1*04:01, HLA-DPA1*02:01/DPB1*01:01, HLA-DPA1*02:01/DPB1*05:01, HLA-DPA1*02:01/DPB1*14:01, HLA-DPA1*03:01/DPB1*04:02, HLA-DQA1*01:01/DQB1*05:01, HLA-DQA1*01:02/DQB1*06:02, HLA-DQA1*03:01/DQB1*03:02, HLA-DQA1*04:01/DQB1*04:02, HLA-DQA1*05:01/DQB1*02:01, HLA-DQA1*05:01/DQB1*03:01, HLA-DRB1*01:01, HLA-DRB1*03:01, HLA-DRB1*04:01, HLA-DRB1*04:05, HLA-DRB1*07:01, HLA-DRB1*08:02, HLA-DRB1*09:01, HLA-DRB1*11:01, HLA-DRB1*12:01, HLA-DRB1*13:02, HLA-DRB1*15:01, HLA-DRB3*01:01, HLA-DRB3*02:02, HLA-DRB4*01:01, HLA-DRB5*01:01 |
| GNNCDLPSRTVDTKQ | HLA-DPA1*01:03/DPB1*02:01, HLA-DPA1*01:03/DPB1*04:01, HLA-DPA1*02:01/DPB1*01:01, HLA-DPA1*02:01/DPB1*05:01, HLA-DPA1*02:01/DPB1*14:01, HLA-DPA1*03:01/DPB1*04:02, HLA-DQA1*01:01/DQB1*05:01, HLA-DQA1*01:02/DQB1*06:02, HLA-DQA1*03:01/DQB1*03:02, HLA-DQA1*04:01/DQB1*04:02, HLA-DQA1*05:01/DQB1*02:01, HLA-DQA1*05:01/DQB1*03:01, HLA-DRB1*01:01, |

| | |
|---|---|
| | HLA-DRB1*03:01, HLA-DRB1*04:01, HLA-DRB1*04:05, HLA-DRB1*07:01, HLA-DRB1*08:02, HLA-DRB1*09:01, HLA-DRB1*11:01, HLA-DRB1*12:01, HLA-DRB1*13:02, HLA-DRB1*15:01, HLA-DRB3*01:01, HLA-DRB3*02:02, HLA-DRB4*01:01, HLA-DRB5*01:01 |
| HEEYSAMRDQYMRTG | HLA-DPA1*01:03/DPB1*02:01, HLA-DPA1*01:03/DPB1*04:01, HLA-DPA1*02:01/DPB1*01:01, HLA-DPA1*02:01/DPB1*05:01, HLA-DPA1*02:01/DPB1*14:01, HLA-DPA1*03:01/DPB1*04:02, HLA-DQA1*01:01/DQB1*05:01, HLA-DQA1*01:02/DQB1*06:02, HLA-DQA1*03:01/DQB1*03:02, HLA-DQA1*04:01/DQB1*04:02, HLA-DQA1*05:01/DQB1*02:01, HLA-DQA1*05:01/DQB1*03:01, HLA-DRB1*01:01, HLA-DRB1*03:01, HLA-DRB1*04:01, HLA-DRB1*04:05, HLA-DRB1*07:01, HLA-DRB1*08:02, HLA-DRB1*09:01, HLA-DRB1*11:01, HLA-DRB1*12:01, HLA-DRB1*13:02, HLA-DRB1*15:01, HLA-DRB3*01:01, HLA-DRB3*02:02, HLA-DRB4*01:01, HLA-DRB5*01:01 |
| HFVDEYDPTIEDSYR | HLA-DPA1*01:03/DPB1*02:01, HLA-DPA1*01:03/DPB1*04:01, HLA-DPA1*02:01/DPB1*01:01, HLA-DPA1*02:01/DPB1*05:01, HLA-DPA1*02:01/DPB1*14:01, HLA-DPA1*03:01/DPB1*04:02, HLA-DQA1*01:01/DQB1*05:01, HLA-DQA1*01:02/DQB1*06:02, HLA-DQA1*03:01/DQB1*03:02, HLA-DQA1*04:01/DQB1*04:02, HLA-DQA1*05:01/DQB1*02:01, HLA-DQA1*05:01/DQB1*03:01, HLA-DRB1*01:01, HLA-DRB1*03:01, HLA-DRB1*04:01, HLA-DRB1*04:05, HLA-DRB1*07:01, HLA-DRB1*08:02, HLA-DRB1*09:01, HLA-DRB1*11:01, HLA-DRB1*12:01, HLA-DRB1*13:02, HLA-DRB1*15:01, HLA-DRB3*01:01, HLA-DRB3*02:02, HLA-DRB4*01:01, HLA-DRB5*01:01 |
| IEDSYRKQVVIDGET | HLA-DPA1*01:03/DPB1*02:01, HLA-DPA1*01:03/DPB1*04:01, HLA-DPA1*02:01/DPB1*01:01, HLA-DPA1*02:01/DPB1*05:01, HLA-DPA1*02:01/DPB1*14:01, HLA-DPA1*03:01/DPB1*04:02, HLA-DQA1*01:01/DQB1*05:01, HLA-DQA1*01:02/DQB1*06:02, HLA-DQA1*03:01/DQB1*03:02, HLA-DQA1*04:01/DQB1*04:02, HLA-DQA1*05:01/DQB1*02:01, HLA-DQA1*05:01/DQB1*03:01, HLA-DRB1*01:01, HLA-DRB1*03:01, HLA-DRB1*04:01, HLA-DRB1*04:05, HLA-DRB1*07:01, HLA-DRB1*08:02, HLA-DRB1*09:01, HLA-DRB1*11:01, HLA-DRB1*12:01, HLA-DRB1*13:02, HLA-DRB1*15:01, HLA-DRB3*01:01, HLA-DRB3*02:02, HLA-DRB4*01:01, HLA-DRB5*01:01 |
| IKKISKEEKTPGCVK | HLA-DPA1*01:03/DPB1*02:01, HLA-DPA1*01:03/DPB1*04:01, HLA-DPA1*02:01/DPB1*01:01, HLA-DPA1*02:01/DPB1*05:01, HLA-DPA1*02:01/DPB1*14:01, HLA-DPA1*03:01/DPB1*04:02, HLA-DQA1*01:01/DQB1*05:01, HLA-DQA1*01:02/DQB1*06:02, HLA-DQA1*03:01/DQB1*03:02, HLA-DQA1*04:01/DQB1*04:02, HLA-DQA1*05:01/DQB1*02:01, HLA-DQA1*05:01/DQB1*03:01, HLA-DRB1*01:01, HLA-DRB1*03:01, HLA-DRB1*04:01, HLA-DRB1*04:05, HLA-DRB1*07:01, HLA-DRB1*08:02, HLA-DRB1*09:01, HLA-DRB1*11:01, HLA-DRB1*12:01, HLA-DRB1*13:02, HLA-DRB1*15:01, HLA-DRB3*01:01, HLA-DRB3*02:02, HLA-DRB4*01:01, HLA-DRB5*01:01 |
| ILDTTDHEEYSAMRD | HLA-DPA1*01:03/DPB1*02:01, HLA-DPA1*01:03/DPB1*04:01, HLA-DPA1*02:01/DPB1*01:01, HLA-DPA1*02:01/DPB1*05:01, HLA-DPA1*02:01/DPB1*14:01, HLA-DPA1*03:01/DPB1*04:02, HLA-DQA1*01:01/DQB1*05:01, HLA-DQA1*01:02/DQB1*06:02, HLA-DQA1*03:01/DQB1*03:02, HLA-DQA1*04:01/DQB1*04:02, HLA-DQA1*05:01/DQB1*02:01, HLA-DQA1*05:01/DQB1*03:01, HLA-DRB1*01:01, HLA-DRB1*03:01, HLA-DRB1*04:01, HLA-DRB1*04:05, HLA-DRB1*07:01, HLA-DRB1*08:02, HLA-DRB1*09:01, HLA-DRB1*11:01, HLA-DRB1*12:01, HLA-DRB1*13:02, HLA-DRB1*15:01, HLA-DRB3*01:01, HLA-DRB3*02:02, HLA-DRB4*01:01, HLA-DRB5*01:01 |
| INNTKSFEDIHHYRE | HLA-DPA1*01:03/DPB1*02:01, HLA-DPA1*01:03/DPB1*04:01, HLA-DPA1*02:01/DPB1*01:01, HLA-DPA1*02:01/DPB1*05:01, HLA-DPA1*02:01/DPB1*14:01, HLA-DPA1*03:01/DPB1*04:02, HLA-DQA1*01:01/DQB1*05:01, HLA-DQA1*01:02/DQB1*06:02, HLA-DQA1*03:01/DQB1*03:02, HLA-DQA1*04:01/DQB1*04:02, HLA-DQA1*05:01/DQB1*02:01, HLA-DQA1*05:01/DQB1*03:01, HLA-DRB1*01:01, |

| | |
|---|---|
| | HLA-DRB1*03:01, HLA-DRB1*04:01, HLA-DRB1*04:05, HLA-DRB1*07:01, HLA-DRB1*08:02, HLA-DRB1*09:01, HLA-DRB1*11:01, HLA-DRB1*12:01, HLA-DRB1*13:02, HLA-DRB1*15:01, HLA-DRB3*01:01, HLA-DRB3*02:02, HLA-DRB4*01:01, HLA-DRB5*01:01 |
| IQLIQNHFVDEYDPT | HLA-DPA1*01:03/DPB1*02:01, HLA-DPA1*01:03/DPB1*04:01, HLA-DPA1*02:01/DPB1*01:01, HLA-DPA1*02:01/DPB1*05:01, HLA-DPA1*02:01/DPB1*14:01, HLA-DPA1*03:01/DPB1*04:02, HLA-DQA1*01:01/DQB1*05:01, HLA-DQA1*01:02/DQB1*06:02, HLA-DQA1*03:01/DQB1*03:02, HLA-DQA1*04:01/DQB1*04:02, HLA-DQA1*05:01/DQB1*02:01, HLA-DQA1*05:01/DQB1*03:01, HLA-DRB1*01:01, HLA-DRB1*03:01, HLA-DRB1*04:01, HLA-DRB1*04:05, HLA-DRB1*07:01, HLA-DRB1*08:02, HLA-DRB1*09:01, HLA-DRB1*11:01, HLA-DRB1*12:01, HLA-DRB1*13:02, HLA-DRB1*15:01, HLA-DRB3*01:01, HLA-DRB3*02:02, HLA-DRB4*01:01, HLA-DRB5*01:01 |
| IQNHFVDEYDPTIED | HLA-DPA1*01:03/DPB1*02:01, HLA-DPA1*01:03/DPB1*04:01, HLA-DPA1*02:01/DPB1*01:01, HLA-DPA1*02:01/DPB1*05:01, HLA-DPA1*02:01/DPB1*14:01, HLA-DPA1*03:01/DPB1*04:02, HLA-DQA1*01:01/DQB1*05:01, HLA-DQA1*01:02/DQB1*06:02, HLA-DQA1*03:01/DQB1*03:02, HLA-DQA1*04:01/DQB1*04:02, HLA-DQA1*05:01/DQB1*02:01, HLA-DQA1*05:01/DQB1*03:01, HLA-DRB1*01:01, HLA-DRB1*03:01, HLA-DRB1*04:01, HLA-DRB1*04:05, HLA-DRB1*07:01, HLA-DRB1*08:02, HLA-DRB1*09:01, HLA-DRB1*11:01, HLA-DRB1*12:01, HLA-DRB1*13:02, HLA-DRB1*15:01, HLA-DRB3*01:01, HLA-DRB3*02:02, HLA-DRB4*01:01, HLA-DRB5*01:01 |
| IRQYFIKKISKEEKT | HLA-DPA1*01:03/DPB1*02:01, HLA-DPA1*01:03/DPB1*04:01, HLA-DPA1*02:01/DPB1*01:01, HLA-DPA1*02:01/DPB1*05:01, HLA-DPA1*02:01/DPB1*14:01, HLA-DPA1*03:01/DPB1*04:02, HLA-DQA1*01:01/DQB1*05:01, HLA-DQA1*01:02/DQB1*06:02, HLA-DQA1*03:01/DQB1*03:02, HLA-DQA1*04:01/DQB1*04:02, HLA-DQA1*05:01/DQB1*02:01, HLA-DQA1*05:01/DQB1*03:01, HLA-DRB1*01:01, HLA-DRB1*03:01, HLA-DRB1*04:01, HLA-DRB1*04:05, HLA-DRB1*07:01, HLA-DRB1*08:02, HLA-DRB1*09:01, HLA-DRB1*11:01, HLA-DRB1*12:01, HLA-DRB1*13:02, HLA-DRB1*15:01, HLA-DRB3*01:01, HLA-DRB3*02:02, HLA-DRB4*01:01, HLA-DRB5*01:01 |
| ISKEEKTPGCVKIKK | HLA-DPA1*01:03/DPB1*02:01, HLA-DPA1*01:03/DPB1*04:01, HLA-DPA1*02:01/DPB1*01:01, HLA-DPA1*02:01/DPB1*05:01, HLA-DPA1*02:01/DPB1*14:01, HLA-DPA1*03:01/DPB1*04:02, HLA-DQA1*01:01/DQB1*05:01, HLA-DQA1*01:02/DQB1*06:02, HLA-DQA1*03:01/DQB1*03:02, HLA-DQA1*04:01/DQB1*04:02, HLA-DQA1*05:01/DQB1*02:01, HLA-DQA1*05:01/DQB1*03:01, HLA-DRB1*01:01, HLA-DRB1*03:01, HLA-DRB1*04:01, HLA-DRB1*04:05, HLA-DRB1*07:01, HLA-DRB1*08:02, HLA-DRB1*09:01, HLA-DRB1*11:01, HLA-DRB1*12:01, HLA-DRB1*13:02, HLA-DRB1*15:01, HLA-DRB3*01:01, HLA-DRB3*02:02, HLA-DRB4*01:01, HLA-DRB5*01:01 |
| KDSEDVPMVLVGNNC | HLA-DPA1*01:03/DPB1*02:01, HLA-DPA1*01:03/DPB1*04:01, HLA-DPA1*02:01/DPB1*01:01, HLA-DPA1*02:01/DPB1*05:01, HLA-DPA1*02:01/DPB1*14:01, HLA-DPA1*03:01/DPB1*04:02, HLA-DQA1*01:01/DQB1*05:01, HLA-DQA1*01:02/DQB1*06:02, HLA-DQA1*03:01/DQB1*03:02, HLA-DQA1*04:01/DQB1*04:02, HLA-DQA1*05:01/DQB1*02:01, HLA-DQA1*05:01/DQB1*03:01, HLA-DRB1*01:01, HLA-DRB1*03:01, HLA-DRB1*04:01, HLA-DRB1*04:05, HLA-DRB1*07:01, HLA-DRB1*08:02, HLA-DRB1*09:01, HLA-DRB1*11:01, HLA-DRB1*12:01, HLA-DRB1*13:02, HLA-DRB1*15:01, HLA-DRB3*01:01, HLA-DRB3*02:02, HLA-DRB4*01:01, HLA-DRB5*01:01 |
| KEEKTPGCVKIKKCI | HLA-DPA1*01:03/DPB1*02:01, HLA-DPA1*01:03/DPB1*04:01, HLA-DPA1*02:01/DPB1*01:01, HLA-DPA1*02:01/DPB1*05:01, HLA-DPA1*02:01/DPB1*14:01, HLA-DPA1*03:01/DPB1*04:02, HLA-DQA1*01:01/DQB1*05:01, HLA-DQA1*01:02/DQB1*06:02, HLA-DQA1*03:01/DQB1*03:02, HLA-DQA1*04:01/DQB1*04:02, HLA-DQA1*05:01/DQB1*02:01, HLA-DQA1*05:01/DQB1*03:01, HLA-DRB1*01:01, |

| | |
|---|---|
| | HLA-DRB1*03:01, HLA-DRB1*04:01, HLA-DRB1*04:05, HLA-DRB1*07:01, HLA-DRB1*08:02, HLA-DRB1*09:01, HLA-DRB1*11:01, HLA-DRB1*12:01, HLA-DRB1*13:02, HLA-DRB1*15:01, HLA-DRB3*01:01, HLA-DRB3*02:02, HLA-DRB4*01:01, HLA-DRB5*01:01 |
| KISKEEKTPGCVKIK | HLA-DPA1*01:03/DPB1*02:01, HLA-DPA1*01:03/DPB1*04:01, HLA-DPA1*02:01/DPB1*01:01, HLA-DPA1*02:01/DPB1*05:01, HLA-DPA1*02:01/DPB1*14:01, HLA-DPA1*03:01/DPB1*04:02, HLA-DQA1*01:01/DQB1*05:01, HLA-DQA1*01:02/DQB1*06:02, HLA-DQA1*03:01/DQB1*03:02, HLA-DQA1*04:01/DQB1*04:02, HLA-DQA1*05:01/DQB1*02:01, HLA-DQA1*05:01/DQB1*03:01, HLA-DRB1*01:01, HLA-DRB1*03:01, HLA-DRB1*04:01, HLA-DRB1*04:05, HLA-DRB1*07:01, HLA-DRB1*08:02, HLA-DRB1*09:01, HLA-DRB1*11:01, HLA-DRB1*12:01, HLA-DRB1*13:02, HLA-DRB1*15:01, HLA-DRB3*01:01, HLA-DRB3*02:02, HLA-DRB4*01:01, HLA-DRB5*01:01 |
| KKISKEEKTPGCVKI | HLA-DPA1*01:03/DPB1*02:01, HLA-DPA1*01:03/DPB1*04:01, HLA-DPA1*02:01/DPB1*01:01, HLA-DPA1*02:01/DPB1*05:01, HLA-DPA1*02:01/DPB1*14:01, HLA-DPA1*03:01/DPB1*04:02, HLA-DQA1*01:01/DQB1*05:01, HLA-DQA1*01:02/DQB1*06:02, HLA-DQA1*03:01/DQB1*03:02, HLA-DQA1*04:01/DQB1*04:02, HLA-DQA1*05:01/DQB1*02:01, HLA-DQA1*05:01/DQB1*03:01, HLA-DRB1*01:01, HLA-DRB1*03:01, HLA-DRB1*04:01, HLA-DRB1*04:05, HLA-DRB1*07:01, HLA-DRB1*08:02, HLA-DRB1*09:01, HLA-DRB1*11:01, HLA-DRB1*12:01, HLA-DRB1*13:02, HLA-DRB1*15:01, HLA-DRB3*01:01, HLA-DRB3*02:02, HLA-DRB4*01:01, HLA-DRB5*01:01 |
| KLVVVGADDVGKSAF | HLA-DPA1*01:03/DPB1*02:01, HLA-DPA1*01:03/DPB1*04:01, HLA-DPA1*02:01/DPB1*01:01, HLA-DPA1*02:01/DPB1*05:01, HLA-DPA1*02:01/DPB1*14:01, HLA-DPA1*03:01/DPB1*04:02, HLA-DQA1*01:01/DQB1*05:01, HLA-DQA1*01:02/DQB1*06:02, HLA-DQA1*03:01/DQB1*03:02, HLA-DQA1*04:01/DQB1*04:02, HLA-DQA1*05:01/DQB1*02:01, HLA-DQA1*05:01/DQB1*03:01, HLA-DRB1*01:01, HLA-DRB1*03:01, HLA-DRB1*04:01, HLA-DRB1*04:05, HLA-DRB1*07:01, HLA-DRB1*08:02, HLA-DRB1*09:01, HLA-DRB1*11:01, HLA-DRB1*12:01, HLA-DRB1*13:02, HLA-DRB1*15:01, HLA-DRB3*01:01, HLA-DRB3*02:02, HLA-DRB4*01:01, HLA-DRB5*01:01 |
| KQAQDLARSYGIPFI | HLA-DPA1*01:03/DPB1*02:01, HLA-DPA1*01:03/DPB1*04:01, HLA-DPA1*02:01/DPB1*01:01, HLA-DPA1*02:01/DPB1*05:01, HLA-DPA1*02:01/DPB1*14:01, HLA-DPA1*03:01/DPB1*04:02, HLA-DQA1*01:01/DQB1*05:01, HLA-DQA1*01:02/DQB1*06:02, HLA-DQA1*03:01/DQB1*03:02, HLA-DQA1*04:01/DQB1*04:02, HLA-DQA1*05:01/DQB1*02:01, HLA-DQA1*05:01/DQB1*03:01, HLA-DRB1*01:01, HLA-DRB1*03:01, HLA-DRB1*04:01, HLA-DRB1*04:05, HLA-DRB1*07:01, HLA-DRB1*08:02, HLA-DRB1*09:01, HLA-DRB1*11:01, HLA-DRB1*12:01, HLA-DRB1*13:02, HLA-DRB1*15:01, HLA-DRB3*01:01, HLA-DRB3*02:02, HLA-DRB4*01:01, HLA-DRB5*01:01 |
| KRVKDSEDVPMVLVG | HLA-DPA1*01:03/DPB1*02:01, HLA-DPA1*01:03/DPB1*04:01, HLA-DPA1*02:01/DPB1*01:01, HLA-DPA1*02:01/DPB1*05:01, HLA-DPA1*02:01/DPB1*14:01, HLA-DPA1*03:01/DPB1*04:02, HLA-DQA1*01:01/DQB1*05:01, HLA-DQA1*01:02/DQB1*06:02, HLA-DQA1*03:01/DQB1*03:02, HLA-DQA1*04:01/DQB1*04:02, HLA-DQA1*05:01/DQB1*02:01, HLA-DQA1*05:01/DQB1*03:01, HLA-DRB1*01:01, HLA-DRB1*03:01, HLA-DRB1*04:01, HLA-DRB1*04:05, HLA-DRB1*07:01, HLA-DRB1*08:02, HLA-DRB1*09:01, HLA-DRB1*11:01, HLA-DRB1*12:01, HLA-DRB1*13:02, HLA-DRB1*15:01, HLA-DRB3*01:01, HLA-DRB3*02:02, HLA-DRB4*01:01, HLA-DRB5*01:01 |
| KSAFTIQLIQNHFVD | HLA-DPA1*01:03/DPB1*02:01, HLA-DPA1*01:03/DPB1*04:01, HLA-DPA1*02:01/DPB1*01:01, HLA-DPA1*02:01/DPB1*05:01, HLA-DPA1*02:01/DPB1*14:01, HLA-DPA1*03:01/DPB1*04:02, HLA-DQA1*01:01/DQB1*05:01, HLA-DQA1*01:02/DQB1*06:02, HLA-DQA1*03:01/DQB1*03:02, HLA-DQA1*04:01/DQB1*04:02, HLA-DQA1*05:01/DQB1*02:01, HLA-DQA1*05:01/DQB1*03:01, HLA-DRB1*01:01, |

| | |
|---|---|
| | HLA-DRB1*03:01, HLA-DRB1*04:01, HLA-DRB1*04:05, HLA-DRB1*07:01, HLA-DRB1*08:02, HLA-DRB1*09:01, HLA-DRB1*11:01, HLA-DRB1*12:01, HLA-DRB1*13:02, HLA-DRB1*15:01, HLA-DRB3*01:01, HLA-DRB3*02:02, HLA-DRB4*01:01, HLA-DRB5*01:01 |
| LARSYGIPFIETSTK | HLA-DPA1*01:03/DPB1*02:01, HLA-DPA1*01:03/DPB1*04:01, HLA-DPA1*02:01/DPB1*01:01, HLA-DPA1*02:01/DPB1*05:01, HLA-DPA1*02:01/DPB1*14:01, HLA-DPA1*03:01/DPB1*04:02, HLA-DQA1*01:01/DQB1*05:01, HLA-DQA1*01:02/DQB1*06:02, HLA-DQA1*03:01/DQB1*03:02, HLA-DQA1*04:01/DQB1*04:02, HLA-DQA1*05:01/DQB1*02:01, HLA-DQA1*05:01/DQB1*03:01, HLA-DRB1*01:01, HLA-DRB1*03:01, HLA-DRB1*04:01, HLA-DRB1*04:05, HLA-DRB1*07:01, HLA-DRB1*08:02, HLA-DRB1*09:01, HLA-DRB1*11:01, HLA-DRB1*12:01, HLA-DRB1*13:02, HLA-DRB1*15:01, HLA-DRB3*01:01, HLA-DRB3*02:02, HLA-DRB4*01:01, HLA-DRB5*01:01 |
| LCVFAINNTKSFEDI | HLA-DPA1*01:03/DPB1*02:01, HLA-DPA1*01:03/DPB1*04:01, HLA-DPA1*02:01/DPB1*01:01, HLA-DPA1*02:01/DPB1*05:01, HLA-DPA1*02:01/DPB1*14:01, HLA-DPA1*03:01/DPB1*04:02, HLA-DQA1*01:01/DQB1*05:01, HLA-DQA1*01:02/DQB1*06:02, HLA-DQA1*03:01/DQB1*03:02, HLA-DQA1*04:01/DQB1*04:02, HLA-DQA1*05:01/DQB1*02:01, HLA-DQA1*05:01/DQB1*03:01, HLA-DRB1*01:01, HLA-DRB1*03:01, HLA-DRB1*04:01, HLA-DRB1*04:05, HLA-DRB1*07:01, HLA-DRB1*08:02, HLA-DRB1*09:01, HLA-DRB1*11:01, HLA-DRB1*12:01, HLA-DRB1*13:02, HLA-DRB1*15:01, HLA-DRB3*01:01, HLA-DRB3*02:02, HLA-DRB4*01:01, HLA-DRB5*01:01 |
| LDILDTTDHEEYSAM | HLA-DPA1*01:03/DPB1*02:01, HLA-DPA1*01:03/DPB1*04:01, HLA-DPA1*02:01/DPB1*01:01, HLA-DPA1*02:01/DPB1*05:01, HLA-DPA1*02:01/DPB1*14:01, HLA-DPA1*03:01/DPB1*04:02, HLA-DQA1*01:01/DQB1*05:01, HLA-DQA1*01:02/DQB1*06:02, HLA-DQA1*03:01/DQB1*03:02, HLA-DQA1*04:01/DQB1*04:02, HLA-DQA1*05:01/DQB1*02:01, HLA-DQA1*05:01/DQB1*03:01, HLA-DRB1*01:01, HLA-DRB1*03:01, HLA-DRB1*04:01, HLA-DRB1*04:05, HLA-DRB1*07:01, HLA-DRB1*08:02, HLA-DRB1*09:01, HLA-DRB1*11:01, HLA-DRB1*12:01, HLA-DRB1*13:02, HLA-DRB1*15:01, HLA-DRB3*01:01, HLA-DRB3*02:02, HLA-DRB4*01:01, HLA-DRB5*01:01 |
| LDTTDHEEYSAMRDQ | HLA-DPA1*01:03/DPB1*02:01, HLA-DPA1*01:03/DPB1*04:01, HLA-DPA1*02:01/DPB1*01:01, HLA-DPA1*02:01/DPB1*05:01, HLA-DPA1*02:01/DPB1*14:01, HLA-DPA1*03:01/DPB1*04:02, HLA-DQA1*01:01/DQB1*05:01, HLA-DQA1*01:02/DQB1*06:02, HLA-DQA1*03:01/DQB1*03:02, HLA-DQA1*04:01/DQB1*04:02, HLA-DQA1*05:01/DQB1*02:01, HLA-DQA1*05:01/DQB1*03:01, HLA-DRB1*01:01, HLA-DRB1*03:01, HLA-DRB1*04:01, HLA-DRB1*04:05, HLA-DRB1*07:01, HLA-DRB1*08:02, HLA-DRB1*09:01, HLA-DRB1*11:01, HLA-DRB1*12:01, HLA-DRB1*13:02, HLA-DRB1*15:01, HLA-DRB3*01:01, HLA-DRB3*02:02, HLA-DRB4*01:01, HLA-DRB5*01:01 |
| LIQNHFVDEYDPTIE | HLA-DPA1*01:03/DPB1*02:01, HLA-DPA1*01:03/DPB1*04:01, HLA-DPA1*02:01/DPB1*01:01, HLA-DPA1*02:01/DPB1*05:01, HLA-DPA1*02:01/DPB1*14:01, HLA-DPA1*03:01/DPB1*04:02, HLA-DQA1*01:01/DQB1*05:01, HLA-DQA1*01:02/DQB1*06:02, HLA-DQA1*03:01/DQB1*03:02, HLA-DQA1*04:01/DQB1*04:02, HLA-DQA1*05:01/DQB1*02:01, HLA-DQA1*05:01/DQB1*03:01, HLA-DRB1*01:01, HLA-DRB1*03:01, HLA-DRB1*04:01, HLA-DRB1*04:05, HLA-DRB1*07:01, HLA-DRB1*08:02, HLA-DRB1*09:01, HLA-DRB1*11:01, HLA-DRB1*12:01, HLA-DRB1*13:02, HLA-DRB1*15:01, HLA-DRB3*01:01, HLA-DRB3*02:02, HLA-DRB4*01:01, HLA-DRB5*01:01 |
| LLDILDTTDHEEYSA | HLA-DPA1*01:03/DPB1*02:01, HLA-DPA1*01:03/DPB1*04:01, HLA-DPA1*02:01/DPB1*01:01, HLA-DPA1*02:01/DPB1*05:01, HLA-DPA1*02:01/DPB1*14:01, HLA-DPA1*03:01/DPB1*04:02, HLA-DQA1*01:01/DQB1*05:01, HLA-DQA1*01:02/DQB1*06:02, HLA-DQA1*03:01/DQB1*03:02, HLA-DQA1*04:01/DQB1*04:02, HLA-DQA1*05:01/DQB1*02:01, HLA-DQA1*05:01/DQB1*03:01, HLA-DRB1*01:01, |

| | |
|---|---|
| | HLA-DRB1*03:01, HLA-DRB1*04:01, HLA-DRB1*04:05, HLA-DRB1*07:01, HLA-DRB1*08:02, HLA-DRB1*09:01, HLA-DRB1*11:01, HLA-DRB1*12:01, HLA-DRB1*13:02, HLA-DRB1*15:01, HLA-DRB3*01:01, HLA-DRB3*02:02, HLA-DRB4*01:01, HLA-DRB5*01:01 |
| LPSRTVDTKQAQDLA | HLA-DPA1*01:03/DPB1*02:01, HLA-DPA1*01:03/DPB1*04:01, HLA-DPA1*02:01/DPB1*01:01, HLA-DPA1*02:01/DPB1*05:01, HLA-DPA1*02:01/DPB1*14:01, HLA-DPA1*03:01/DPB1*04:02, HLA-DQA1*01:01/DQB1*05:01, HLA-DQA1*01:02/DQB1*06:02, HLA-DQA1*03:01/DQB1*03:02, HLA-DQA1*04:01/DQB1*04:02, HLA-DQA1*05:01/DQB1*02:01, HLA-DQA1*05:01/DQB1*03:01, HLA-DRB1*01:01, HLA-DRB1*03:01, HLA-DRB1*04:01, HLA-DRB1*04:05, HLA-DRB1*07:01, HLA-DRB1*08:02, HLA-DRB1*09:01, HLA-DRB1*11:01, HLA-DRB1*12:01, HLA-DRB1*13:02, HLA-DRB1*15:01, HLA-DRB3*01:01, HLA-DRB3*02:02, HLA-DRB4*01:01, HLA-DRB5*01:01 |
| LVGNNCDLPSRTVDT | HLA-DPA1*01:03/DPB1*02:01, HLA-DPA1*01:03/DPB1*04:01, HLA-DPA1*02:01/DPB1*01:01, HLA-DPA1*02:01/DPB1*05:01, HLA-DPA1*02:01/DPB1*14:01, HLA-DPA1*03:01/DPB1*04:02, HLA-DQA1*01:01/DQB1*05:01, HLA-DQA1*01:02/DQB1*06:02, HLA-DQA1*03:01/DQB1*03:02, HLA-DQA1*04:01/DQB1*04:02, HLA-DQA1*05:01/DQB1*02:01, HLA-DQA1*05:01/DQB1*03:01, HLA-DRB1*01:01, HLA-DRB1*03:01, HLA-DRB1*04:01, HLA-DRB1*04:05, HLA-DRB1*07:01, HLA-DRB1*08:02, HLA-DRB1*09:01, HLA-DRB1*11:01, HLA-DRB1*12:01, HLA-DRB1*13:02, HLA-DRB1*15:01, HLA-DRB3*01:01, HLA-DRB3*02:02, HLA-DRB4*01:01, HLA-DRB5*01:01 |
| LVVVGADDVGKSAFT | HLA-DPA1*01:03/DPB1*02:01, HLA-DPA1*01:03/DPB1*04:01, HLA-DPA1*02:01/DPB1*01:01, HLA-DPA1*02:01/DPB1*05:01, HLA-DPA1*02:01/DPB1*14:01, HLA-DPA1*03:01/DPB1*04:02, HLA-DQA1*01:01/DQB1*05:01, HLA-DQA1*01:02/DQB1*06:02, HLA-DQA1*03:01/DQB1*03:02, HLA-DQA1*04:01/DQB1*04:02, HLA-DQA1*05:01/DQB1*02:01, HLA-DQA1*05:01/DQB1*03:01, HLA-DRB1*01:01, HLA-DRB1*03:01, HLA-DRB1*04:01, HLA-DRB1*04:05, HLA-DRB1*07:01, HLA-DRB1*08:02, HLA-DRB1*09:01, HLA-DRB1*11:01, HLA-DRB1*12:01, HLA-DRB1*13:02, HLA-DRB1*15:01, HLA-DRB3*01:01, HLA-DRB3*02:02, HLA-DRB4*01:01, HLA-DRB5*01:01 |
| LYREIRQYFIKKISK | HLA-DPA1*01:03/DPB1*02:01, HLA-DPA1*01:03/DPB1*04:01, HLA-DPA1*02:01/DPB1*01:01, HLA-DPA1*02:01/DPB1*05:01, HLA-DPA1*02:01/DPB1*14:01, HLA-DPA1*03:01/DPB1*04:02, HLA-DQA1*01:01/DQB1*05:01, HLA-DQA1*01:02/DQB1*06:02, HLA-DQA1*03:01/DQB1*03:02, HLA-DQA1*04:01/DQB1*04:02, HLA-DQA1*05:01/DQB1*02:01, HLA-DQA1*05:01/DQB1*03:01, HLA-DRB1*01:01, HLA-DRB1*03:01, HLA-DRB1*04:01, HLA-DRB1*04:05, HLA-DRB1*07:01, HLA-DRB1*08:02, HLA-DRB1*09:01, HLA-DRB1*11:01, HLA-DRB1*12:01, HLA-DRB1*13:02, HLA-DRB1*15:01, HLA-DRB3*01:01, HLA-DRB3*02:02, HLA-DRB4*01:01, HLA-DRB5*01:01 |
| MRDQYMRTGEGFLCV | HLA-DPA1*01:03/DPB1*02:01, HLA-DPA1*01:03/DPB1*04:01, HLA-DPA1*02:01/DPB1*01:01, HLA-DPA1*02:01/DPB1*05:01, HLA-DPA1*02:01/DPB1*14:01, HLA-DPA1*03:01/DPB1*04:02, HLA-DQA1*01:01/DQB1*05:01, HLA-DQA1*01:02/DQB1*06:02, HLA-DQA1*03:01/DQB1*03:02, HLA-DQA1*04:01/DQB1*04:02, HLA-DQA1*05:01/DQB1*02:01, HLA-DQA1*05:01/DQB1*03:01, HLA-DRB1*01:01, HLA-DRB1*03:01, HLA-DRB1*04:01, HLA-DRB1*04:05, HLA-DRB1*07:01, HLA-DRB1*08:02, HLA-DRB1*09:01, HLA-DRB1*11:01, HLA-DRB1*12:01, HLA-DRB1*13:02, HLA-DRB1*15:01, HLA-DRB3*01:01, HLA-DRB3*02:02, HLA-DRB4*01:01, HLA-DRB5*01:01 |
| MRTGEGFLCVFAINN | HLA-DPA1*01:03/DPB1*02:01, HLA-DPA1*01:03/DPB1*04:01, HLA-DPA1*02:01/DPB1*01:01, HLA-DPA1*02:01/DPB1*05:01, HLA-DPA1*02:01/DPB1*14:01, HLA-DPA1*03:01/DPB1*04:02, HLA-DQA1*01:01/DQB1*05:01, HLA-DQA1*01:02/DQB1*06:02, HLA-DQA1*03:01/DQB1*03:02, HLA-DQA1*04:01/DQB1*04:02, HLA-DQA1*05:01/DQB1*02:01, HLA-DQA1*05:01/DQB1*03:01, HLA-DRB1*01:01, |

| | |
|---|---|
| | HLA-DRB1*03:01, HLA-DRB1*04:01, HLA-DRB1*04:05, HLA-DRB1*07:01, HLA-DRB1*08:02, HLA-DRB1*09:01, HLA-DRB1*11:01, HLA-DRB1*12:01, HLA-DRB1*13:02, HLA-DRB1*15:01, HLA-DRB3*01:01, HLA-DRB3*02:02, HLA-DRB4*01:01, HLA-DRB5*01:01 |
| MTEYKLVVVGADDV G | HLA-DPA1*01:03/DPB1*02:01, HLA-DPA1*01:03/DPB1*04:01, HLA-DPA1*02:01/DPB1*01:01, HLA-DPA1*02:01/DPB1*05:01, HLA-DPA1*02:01/DPB1*14:01, HLA-DPA1*03:01/DPB1*04:02, HLA-DQA1*01:01/DQB1*05:01, HLA-DQA1*01:02/DQB1*06:02, HLA-DQA1*03:01/DQB1*03:02, HLA-DQA1*04:01/DQB1*04:02, HLA-DQA1*05:01/DQB1*02:01, HLA-DQA1*05:01/DQB1*03:01, HLA-DRB1*01:01, HLA-DRB1*03:01, HLA-DRB1*04:01, HLA-DRB1*04:05, HLA-DRB1*07:01, HLA-DRB1*08:02, HLA-DRB1*09:01, HLA-DRB1*11:01, HLA-DRB1*12:01, HLA-DRB1*13:02, HLA-DRB1*15:01, HLA-DRB3*01:01, HLA-DRB3*02:02, HLA-DRB4*01:01, HLA-DRB5*01:01 |
| MVLVGNNCDLPSRTV | HLA-DPA1*01:03/DPB1*02:01, HLA-DPA1*01:03/DPB1*04:01, HLA-DPA1*02:01/DPB1*01:01, HLA-DPA1*02:01/DPB1*05:01, HLA-DPA1*02:01/DPB1*14:01, HLA-DPA1*03:01/DPB1*04:02, HLA-DQA1*01:01/DQB1*05:01, HLA-DQA1*01:02/DQB1*06:02, HLA-DQA1*03:01/DQB1*03:02, HLA-DQA1*04:01/DQB1*04:02, HLA-DQA1*05:01/DQB1*02:01, HLA-DQA1*05:01/DQB1*03:01, HLA-DRB1*01:01, HLA-DRB1*03:01, HLA-DRB1*04:01, HLA-DRB1*04:05, HLA-DRB1*07:01, HLA-DRB1*08:02, HLA-DRB1*09:01, HLA-DRB1*11:01, HLA-DRB1*12:01, HLA-DRB1*13:02, HLA-DRB1*15:01, HLA-DRB3*01:01, HLA-DRB3*02:02, HLA-DRB4*01:01, HLA-DRB5*01:01 |
| NCDLPSRTVDTKQAQ | HLA-DPA1*01:03/DPB1*02:01, HLA-DPA1*01:03/DPB1*04:01, HLA-DPA1*02:01/DPB1*01:01, HLA-DPA1*02:01/DPB1*05:01, HLA-DPA1*02:01/DPB1*14:01, HLA-DPA1*03:01/DPB1*04:02, HLA-DQA1*01:01/DQB1*05:01, HLA-DQA1*01:02/DQB1*06:02, HLA-DQA1*03:01/DQB1*03:02, HLA-DQA1*04:01/DQB1*04:02, HLA-DQA1*05:01/DQB1*02:01, HLA-DQA1*05:01/DQB1*03:01, HLA-DRB1*01:01, HLA-DRB1*03:01, HLA-DRB1*04:01, HLA-DRB1*04:05, HLA-DRB1*07:01, HLA-DRB1*08:02, HLA-DRB1*09:01, HLA-DRB1*11:01, HLA-DRB1*12:01, HLA-DRB1*13:02, HLA-DRB1*15:01, HLA-DRB3*01:01, HLA-DRB3*02:02, HLA-DRB4*01:01, HLA-DRB5*01:01 |
| NHFVDEYDPTIEDSY | HLA-DPA1*01:03/DPB1*02:01, HLA-DPA1*01:03/DPB1*04:01, HLA-DPA1*02:01/DPB1*01:01, HLA-DPA1*02:01/DPB1*05:01, HLA-DPA1*02:01/DPB1*14:01, HLA-DPA1*03:01/DPB1*04:02, HLA-DQA1*01:01/DQB1*05:01, HLA-DQA1*01:02/DQB1*06:02, HLA-DQA1*03:01/DQB1*03:02, HLA-DQA1*04:01/DQB1*04:02, HLA-DQA1*05:01/DQB1*02:01, HLA-DQA1*05:01/DQB1*03:01, HLA-DRB1*01:01, HLA-DRB1*03:01, HLA-DRB1*04:01, HLA-DRB1*04:05, HLA-DRB1*07:01, HLA-DRB1*08:02, HLA-DRB1*09:01, HLA-DRB1*11:01, HLA-DRB1*12:01, HLA-DRB1*13:02, HLA-DRB1*15:01, HLA-DRB3*01:01, HLA-DRB3*02:02, HLA-DRB4*01:01, HLA-DRB5*01:01 |
| NNCDLPSRTVDTKQA | HLA-DPA1*01:03/DPB1*02:01, HLA-DPA1*01:03/DPB1*04:01, HLA-DPA1*02:01/DPB1*01:01, HLA-DPA1*02:01/DPB1*05:01, HLA-DPA1*02:01/DPB1*14:01, HLA-DPA1*03:01/DPB1*04:02, HLA-DQA1*01:01/DQB1*05:01, HLA-DQA1*01:02/DQB1*06:02, HLA-DQA1*03:01/DQB1*03:02, HLA-DQA1*04:01/DQB1*04:02, HLA-DQA1*05:01/DQB1*02:01, HLA-DQA1*05:01/DQB1*03:01, HLA-DRB1*01:01, HLA-DRB1*03:01, HLA-DRB1*04:01, HLA-DRB1*04:05, HLA-DRB1*07:01, HLA-DRB1*08:02, HLA-DRB1*09:01, HLA-DRB1*11:01, HLA-DRB1*12:01, HLA-DRB1*13:02, HLA-DRB1*15:01, HLA-DRB3*01:01, HLA-DRB3*02:02, HLA-DRB4*01:01, HLA-DRB5*01:01 |
| NNTKSFEDIHHYREQ | HLA-DPA1*01:03/DPB1*02:01, HLA-DPA1*01:03/DPB1*04:01, HLA-DPA1*02:01/DPB1*01:01, HLA-DPA1*02:01/DPB1*05:01, HLA-DPA1*02:01/DPB1*14:01, HLA-DPA1*03:01/DPB1*04:02, HLA-DQA1*01:01/DQB1*05:01, HLA-DQA1*01:02/DQB1*06:02, HLA-DQA1*03:01/DQB1*03:02, HLA-DQA1*04:01/DQB1*04:02, HLA-DQA1*05:01/DQB1*02:01, HLA-DQA1*05:01/DQB1*03:01, HLA-DRB1*01:01, |

| | |
|---|---|
| | HLA-DRB1*03:01, HLA-DRB1*04:01, HLA-DRB1*04:05, HLA-DRB1*07:01, HLA-DRB1*08:02, HLA-DRB1*09:01, HLA-DRB1*11:01, HLA-DRB1*12:01, HLA-DRB1*13:02, HLA-DRB1*15:01, HLA-DRB3*01:01, HLA-DRB3*02:02, HLA-DRB4*01:01, HLA-DRB5*01:01 |
| NTKSFEDIHHYREQI | HLA-DPA1*01:03/DPB1*02:01, HLA-DPA1*01:03/DPB1*04:01, HLA-DPA1*02:01/DPB1*01:01, HLA-DPA1*02:01/DPB1*05:01, HLA-DPA1*02:01/DPB1*14:01, HLA-DPA1*03:01/DPB1*04:02, HLA-DQA1*01:01/DQB1*05:01, HLA-DQA1*01:02/DQB1*06:02, HLA-DQA1*03:01/DQB1*03:02, HLA-DQA1*04:01/DQB1*04:02, HLA-DQA1*05:01/DQB1*02:01, HLA-DQA1*05:01/DQB1*03:01, HLA-DRB1*01:01, HLA-DRB1*03:01, HLA-DRB1*04:01, HLA-DRB1*04:05, HLA-DRB1*07:01, HLA-DRB1*08:02, HLA-DRB1*09:01, HLA-DRB1*11:01, HLA-DRB1*12:01, HLA-DRB1*13:02, HLA-DRB1*15:01, HLA-DRB3*01:01, HLA-DRB3*02:02, HLA-DRB4*01:01, HLA-DRB5*01:01 |
| PMVLVGNNCDLPSRT | HLA-DPA1*01:03/DPB1*02:01, HLA-DPA1*01:03/DPB1*04:01, HLA-DPA1*02:01/DPB1*01:01, HLA-DPA1*02:01/DPB1*05:01, HLA-DPA1*02:01/DPB1*14:01, HLA-DPA1*03:01/DPB1*04:02, HLA-DQA1*01:01/DQB1*05:01, HLA-DQA1*01:02/DQB1*06:02, HLA-DQA1*03:01/DQB1*03:02, HLA-DQA1*04:01/DQB1*04:02, HLA-DQA1*05:01/DQB1*02:01, HLA-DQA1*05:01/DQB1*03:01, HLA-DRB1*01:01, HLA-DRB1*03:01, HLA-DRB1*04:01, HLA-DRB1*04:05, HLA-DRB1*07:01, HLA-DRB1*08:02, HLA-DRB1*09:01, HLA-DRB1*11:01, HLA-DRB1*12:01, HLA-DRB1*13:02, HLA-DRB1*15:01, HLA-DRB3*01:01, HLA-DRB3*02:02, HLA-DRB4*01:01, HLA-DRB5*01:01 |
| PSRTVDTKQAQDLAR | HLA-DPA1*01:03/DPB1*02:01, HLA-DPA1*01:03/DPB1*04:01, HLA-DPA1*02:01/DPB1*01:01, HLA-DPA1*02:01/DPB1*05:01, HLA-DPA1*02:01/DPB1*14:01, HLA-DPA1*03:01/DPB1*04:02, HLA-DQA1*01:01/DQB1*05:01, HLA-DQA1*01:02/DQB1*06:02, HLA-DQA1*03:01/DQB1*03:02, HLA-DQA1*04:01/DQB1*04:02, HLA-DQA1*05:01/DQB1*02:01, HLA-DQA1*05:01/DQB1*03:01, HLA-DRB1*01:01, HLA-DRB1*03:01, HLA-DRB1*04:01, HLA-DRB1*04:05, HLA-DRB1*07:01, HLA-DRB1*08:02, HLA-DRB1*09:01, HLA-DRB1*11:01, HLA-DRB1*12:01, HLA-DRB1*13:02, HLA-DRB1*15:01, HLA-DRB3*01:01, HLA-DRB3*02:02, HLA-DRB4*01:01, HLA-DRB5*01:01 |
| PTIEDSYRKQVVIDG | HLA-DPA1*01:03/DPB1*02:01, HLA-DPA1*01:03/DPB1*04:01, HLA-DPA1*02:01/DPB1*01:01, HLA-DPA1*02:01/DPB1*05:01, HLA-DPA1*02:01/DPB1*14:01, HLA-DPA1*03:01/DPB1*04:02, HLA-DQA1*01:01/DQB1*05:01, HLA-DQA1*01:02/DQB1*06:02, HLA-DQA1*03:01/DQB1*03:02, HLA-DQA1*04:01/DQB1*04:02, HLA-DQA1*05:01/DQB1*02:01, HLA-DQA1*05:01/DQB1*03:01, HLA-DRB1*01:01, HLA-DRB1*03:01, HLA-DRB1*04:01, HLA-DRB1*04:05, HLA-DRB1*07:01, HLA-DRB1*08:02, HLA-DRB1*09:01, HLA-DRB1*11:01, HLA-DRB1*12:01, HLA-DRB1*13:02, HLA-DRB1*15:01, HLA-DRB3*01:01, HLA-DRB3*02:02, HLA-DRB4*01:01, HLA-DRB5*01:01 |
| QAQDLARSYGIPFIE | HLA-DPA1*01:03/DPB1*02:01, HLA-DPA1*01:03/DPB1*04:01, HLA-DPA1*02:01/DPB1*01:01, HLA-DPA1*02:01/DPB1*05:01, HLA-DPA1*02:01/DPB1*14:01, HLA-DPA1*03:01/DPB1*04:02, HLA-DQA1*01:01/DQB1*05:01, HLA-DQA1*01:02/DQB1*06:02, HLA-DQA1*03:01/DQB1*03:02, HLA-DQA1*04:01/DQB1*04:02, HLA-DQA1*05:01/DQB1*02:01, HLA-DQA1*05:01/DQB1*03:01, HLA-DRB1*01:01, HLA-DRB1*03:01, HLA-DRB1*04:01, HLA-DRB1*04:05, HLA-DRB1*07:01, HLA-DRB1*08:02, HLA-DRB1*09:01, HLA-DRB1*11:01, HLA-DRB1*12:01, HLA-DRB1*13:02, HLA-DRB1*15:01, HLA-DRB3*01:01, HLA-DRB3*02:02, HLA-DRB4*01:01, HLA-DRB5*01:01 |
| QDLARSYGIPFIETS | HLA-DPA1*01:03/DPB1*02:01, HLA-DPA1*01:03/DPB1*04:01, HLA-DPA1*02:01/DPB1*01:01, HLA-DPA1*02:01/DPB1*05:01, HLA-DPA1*02:01/DPB1*14:01, HLA-DPA1*03:01/DPB1*04:02, HLA-DQA1*01:01/DQB1*05:01, HLA-DQA1*01:02/DQB1*06:02, HLA-DQA1*03:01/DQB1*03:02, HLA-DQA1*04:01/DQB1*04:02, HLA-DQA1*05:01/DQB1*02:01, HLA-DQA1*05:01/DQB1*03:01, HLA-DRB1*01:01, |

| | |
|---|---|
| | HLA-DRB1*03:01, HLA-DRB1*04:01, HLA-DRB1*04:05, HLA-DRB1*07:01, HLA-DRB1*08:02, HLA-DRB1*09:01, HLA-DRB1*11:01, HLA-DRB1*12:01, HLA-DRB1*13:02, HLA-DRB1*15:01, HLA-DRB3*01:01, HLA-DRB3*02:02, HLA-DRB4*01:01, HLA-DRB5*01:01 |
| QLIQNHFVDEYDPTI | HLA-DPA1*01:03/DPB1*02:01, HLA-DPA1*01:03/DPB1*04:01, HLA-DPA1*02:01/DPB1*01:01, HLA-DPA1*02:01/DPB1*05:01, HLA-DPA1*02:01/DPB1*14:01, HLA-DPA1*03:01/DPB1*04:02, HLA-DQA1*01:01/DQB1*05:01, HLA-DQA1*01:02/DQB1*06:02, HLA-DQA1*03:01/DQB1*03:02, HLA-DQA1*04:01/DQB1*04:02, HLA-DQA1*05:01/DQB1*02:01, HLA-DQA1*05:01/DQB1*03:01, HLA-DRB1*01:01, HLA-DRB1*03:01, HLA-DRB1*04:01, HLA-DRB1*04:05, HLA-DRB1*07:01, HLA-DRB1*08:02, HLA-DRB1*09:01, HLA-DRB1*11:01, HLA-DRB1*12:01, HLA-DRB1*13:02, HLA-DRB1*15:01, HLA-DRB3*01:01, HLA-DRB3*02:02, HLA-DRB4*01:01, HLA-DRB5*01:01 |
| QNHFVDEYDPTIEDS | HLA-DPA1*01:03/DPB1*02:01, HLA-DPA1*01:03/DPB1*04:01, HLA-DPA1*02:01/DPB1*01:01, HLA-DPA1*02:01/DPB1*05:01, HLA-DPA1*02:01/DPB1*14:01, HLA-DPA1*03:01/DPB1*04:02, HLA-DQA1*01:01/DQB1*05:01, HLA-DQA1*01:02/DQB1*06:02, HLA-DQA1*03:01/DQB1*03:02, HLA-DQA1*04:01/DQB1*04:02, HLA-DQA1*05:01/DQB1*02:01, HLA-DQA1*05:01/DQB1*03:01, HLA-DRB1*01:01, HLA-DRB1*03:01, HLA-DRB1*04:01, HLA-DRB1*04:05, HLA-DRB1*07:01, HLA-DRB1*08:02, HLA-DRB1*09:01, HLA-DRB1*11:01, HLA-DRB1*12:01, HLA-DRB1*13:02, HLA-DRB1*15:01, HLA-DRB3*01:01, HLA-DRB3*02:02, HLA-DRB4*01:01, HLA-DRB5*01:01 |
| QYFIKKISKEEKTPG | HLA-DPA1*01:03/DPB1*02:01, HLA-DPA1*01:03/DPB1*04:01, HLA-DPA1*02:01/DPB1*01:01, HLA-DPA1*02:01/DPB1*05:01, HLA-DPA1*02:01/DPB1*14:01, HLA-DPA1*03:01/DPB1*04:02, HLA-DQA1*01:01/DQB1*05:01, HLA-DQA1*01:02/DQB1*06:02, HLA-DQA1*03:01/DQB1*03:02, HLA-DQA1*04:01/DQB1*04:02, HLA-DQA1*05:01/DQB1*02:01, HLA-DQA1*05:01/DQB1*03:01, HLA-DRB1*01:01, HLA-DRB1*03:01, HLA-DRB1*04:01, HLA-DRB1*04:05, HLA-DRB1*07:01, HLA-DRB1*08:02, HLA-DRB1*09:01, HLA-DRB1*11:01, HLA-DRB1*12:01, HLA-DRB1*13:02, HLA-DRB1*15:01, HLA-DRB3*01:01, HLA-DRB3*02:02, HLA-DRB4*01:01, HLA-DRB5*01:01 |
| QYMRTGEGFLCVFAI | HLA-DPA1*01:03/DPB1*02:01, HLA-DPA1*01:03/DPB1*04:01, HLA-DPA1*02:01/DPB1*01:01, HLA-DPA1*02:01/DPB1*05:01, HLA-DPA1*02:01/DPB1*14:01, HLA-DPA1*03:01/DPB1*04:02, HLA-DQA1*01:01/DQB1*05:01, HLA-DQA1*01:02/DQB1*06:02, HLA-DQA1*03:01/DQB1*03:02, HLA-DQA1*04:01/DQB1*04:02, HLA-DQA1*05:01/DQB1*02:01, HLA-DQA1*05:01/DQB1*03:01, HLA-DRB1*01:01, HLA-DRB1*03:01, HLA-DRB1*04:01, HLA-DRB1*04:05, HLA-DRB1*07:01, HLA-DRB1*08:02, HLA-DRB1*09:01, HLA-DRB1*11:01, HLA-DRB1*12:01, HLA-DRB1*13:02, HLA-DRB1*15:01, HLA-DRB3*01:01, HLA-DRB3*02:02, HLA-DRB4*01:01, HLA-DRB5*01:01 |
| RDQYMRTGEGFLCVF | HLA-DPA1*01:03/DPB1*02:01, HLA-DPA1*01:03/DPB1*04:01, HLA-DPA1*02:01/DPB1*01:01, HLA-DPA1*02:01/DPB1*05:01, HLA-DPA1*02:01/DPB1*14:01, HLA-DPA1*03:01/DPB1*04:02, HLA-DQA1*01:01/DQB1*05:01, HLA-DQA1*01:02/DQB1*06:02, HLA-DQA1*03:01/DQB1*03:02, HLA-DQA1*04:01/DQB1*04:02, HLA-DQA1*05:01/DQB1*02:01, HLA-DQA1*05:01/DQB1*03:01, HLA-DRB1*01:01, HLA-DRB1*03:01, HLA-DRB1*04:01, HLA-DRB1*04:05, HLA-DRB1*07:01, HLA-DRB1*08:02, HLA-DRB1*09:01, HLA-DRB1*11:01, HLA-DRB1*12:01, HLA-DRB1*13:02, HLA-DRB1*15:01, HLA-DRB3*01:01, HLA-DRB3*02:02, HLA-DRB4*01:01, HLA-DRB5*01:01 |
| REIRQYFIKKISKEE | HLA-DPA1*01:03/DPB1*02:01, HLA-DPA1*01:03/DPB1*04:01, HLA-DPA1*02:01/DPB1*01:01, HLA-DPA1*02:01/DPB1*05:01, HLA-DPA1*02:01/DPB1*14:01, HLA-DPA1*03:01/DPB1*04:02, HLA-DQA1*01:01/DQB1*05:01, HLA-DQA1*01:02/DQB1*06:02, HLA-DQA1*03:01/DQB1*03:02, HLA-DQA1*04:01/DQB1*04:02, HLA-DQA1*05:01/DQB1*02:01, HLA-DQA1*05:01/DQB1*03:01, HLA-DRB1*01:01, |

| | |
|---|---|
| | HLA-DRB1*03:01, HLA-DRB1*04:01, HLA-DRB1*04:05, HLA-DRB1*07:01, HLA-DRB1*08:02, HLA-DRB1*09:01, HLA-DRB1*11:01, HLA-DRB1*12:01, HLA-DRB1*13:02, HLA-DRB1*15:01, HLA-DRB3*01:01, HLA-DRB3*02:02, HLA-DRB4*01:01, HLA-DRB5*01:01 |
| RQYFIKKISKEEKTP | HLA-DPA1*01:03/DPB1*02:01, HLA-DPA1*01:03/DPB1*04:01, HLA-DPA1*02:01/DPB1*01:01, HLA-DPA1*02:01/DPB1*05:01, HLA-DPA1*02:01/DPB1*14:01, HLA-DPA1*03:01/DPB1*04:02, HLA-DQA1*01:01/DQB1*05:01, HLA-DQA1*01:02/DQB1*06:02, HLA-DQA1*03:01/DQB1*03:02, HLA-DQA1*04:01/DQB1*04:02, HLA-DQA1*05:01/DQB1*02:01, HLA-DQA1*05:01/DQB1*03:01, HLA-DRB1*01:01, HLA-DRB1*03:01, HLA-DRB1*04:01, HLA-DRB1*04:05, HLA-DRB1*07:01, HLA-DRB1*08:02, HLA-DRB1*09:01, HLA-DRB1*11:01, HLA-DRB1*12:01, HLA-DRB1*13:02, HLA-DRB1*15:01, HLA-DRB3*01:01, HLA-DRB3*02:02, HLA-DRB4*01:01, HLA-DRB5*01:01 |
| RSYGIPFIETSTKTR | HLA-DPA1*01:03/DPB1*02:01, HLA-DPA1*01:03/DPB1*04:01, HLA-DPA1*02:01/DPB1*01:01, HLA-DPA1*02:01/DPB1*05:01, HLA-DPA1*02:01/DPB1*14:01, HLA-DPA1*03:01/DPB1*04:02, HLA-DQA1*01:01/DQB1*05:01, HLA-DQA1*01:02/DQB1*06:02, HLA-DQA1*03:01/DQB1*03:02, HLA-DQA1*04:01/DQB1*04:02, HLA-DQA1*05:01/DQB1*02:01, HLA-DQA1*05:01/DQB1*03:01, HLA-DRB1*01:01, HLA-DRB1*03:01, HLA-DRB1*04:01, HLA-DRB1*04:05, HLA-DRB1*07:01, HLA-DRB1*08:02, HLA-DRB1*09:01, HLA-DRB1*11:01, HLA-DRB1*12:01, HLA-DRB1*13:02, HLA-DRB1*15:01, HLA-DRB3*01:01, HLA-DRB3*02:02, HLA-DRB4*01:01, HLA-DRB5*01:01 |
| RTGEGFLCVFAINNT | HLA-DPA1*01:03/DPB1*02:01, HLA-DPA1*01:03/DPB1*04:01, HLA-DPA1*02:01/DPB1*01:01, HLA-DPA1*02:01/DPB1*05:01, HLA-DPA1*02:01/DPB1*14:01, HLA-DPA1*03:01/DPB1*04:02, HLA-DQA1*01:01/DQB1*05:01, HLA-DQA1*01:02/DQB1*06:02, HLA-DQA1*03:01/DQB1*03:02, HLA-DQA1*04:01/DQB1*04:02, HLA-DQA1*05:01/DQB1*02:01, HLA-DQA1*05:01/DQB1*03:01, HLA-DRB1*01:01, HLA-DRB1*03:01, HLA-DRB1*04:01, HLA-DRB1*04:05, HLA-DRB1*07:01, HLA-DRB1*08:02, HLA-DRB1*09:01, HLA-DRB1*11:01, HLA-DRB1*12:01, HLA-DRB1*13:02, HLA-DRB1*15:01, HLA-DRB3*01:01, HLA-DRB3*02:02, HLA-DRB4*01:01, HLA-DRB5*01:01 |
| RTVDTKQAQDLARSY | HLA-DPA1*01:03/DPB1*02:01, HLA-DPA1*01:03/DPB1*04:01, HLA-DPA1*02:01/DPB1*01:01, HLA-DPA1*02:01/DPB1*05:01, HLA-DPA1*02:01/DPB1*14:01, HLA-DPA1*03:01/DPB1*04:02, HLA-DQA1*01:01/DQB1*05:01, HLA-DQA1*01:02/DQB1*06:02, HLA-DQA1*03:01/DQB1*03:02, HLA-DQA1*04:01/DQB1*04:02, HLA-DQA1*05:01/DQB1*02:01, HLA-DQA1*05:01/DQB1*03:01, HLA-DRB1*01:01, HLA-DRB1*03:01, HLA-DRB1*04:01, HLA-DRB1*04:05, HLA-DRB1*07:01, HLA-DRB1*08:02, HLA-DRB1*09:01, HLA-DRB1*11:01, HLA-DRB1*12:01, HLA-DRB1*13:02, HLA-DRB1*15:01, HLA-DRB3*01:01, HLA-DRB3*02:02, HLA-DRB4*01:01, HLA-DRB5*01:01 |
| RVEDAFYTLYREIRQ | HLA-DPA1*01:03/DPB1*02:01, HLA-DPA1*01:03/DPB1*04:01, HLA-DPA1*02:01/DPB1*01:01, HLA-DPA1*02:01/DPB1*05:01, HLA-DPA1*02:01/DPB1*14:01, HLA-DPA1*03:01/DPB1*04:02, HLA-DQA1*01:01/DQB1*05:01, HLA-DQA1*01:02/DQB1*06:02, HLA-DQA1*03:01/DQB1*03:02, HLA-DQA1*04:01/DQB1*04:02, HLA-DQA1*05:01/DQB1*02:01, HLA-DQA1*05:01/DQB1*03:01, HLA-DRB1*01:01, HLA-DRB1*03:01, HLA-DRB1*04:01, HLA-DRB1*04:05, HLA-DRB1*07:01, HLA-DRB1*08:02, HLA-DRB1*09:01, HLA-DRB1*11:01, HLA-DRB1*12:01, HLA-DRB1*13:02, HLA-DRB1*15:01, HLA-DRB3*01:01, HLA-DRB3*02:02, HLA-DRB4*01:01, HLA-DRB5*01:01 |
| RVKDSEDVPMVLVGN | HLA-DPA1*01:03/DPB1*02:01, HLA-DPA1*01:03/DPB1*04:01, HLA-DPA1*02:01/DPB1*01:01, HLA-DPA1*02:01/DPB1*05:01, HLA-DPA1*02:01/DPB1*14:01, HLA-DPA1*03:01/DPB1*04:02, HLA-DQA1*01:01/DQB1*05:01, HLA-DQA1*01:02/DQB1*06:02, HLA-DQA1*03:01/DQB1*03:02, HLA-DQA1*04:01/DQB1*04:02, HLA-DQA1*05:01/DQB1*02:01, HLA-DQA1*05:01/DQB1*03:01, HLA-DRB1*01:01, |

| | |
|---|---|
| | HLA-DRB1*03:01, HLA-DRB1*04:01, HLA-DRB1*04:05, HLA-DRB1*07:01,<br>HLA-DRB1*08:02, HLA-DRB1*09:01, HLA-DRB1*11:01, HLA-DRB1*12:01,<br>HLA-DRB1*13:02, HLA-DRB1*15:01, HLA-DRB3*01:01, HLA-DRB3*02:02,<br>HLA-DRB4*01:01, HLA-DRB5*01:01 |
| SAFTIQLIQNHFVDE | HLA-DPA1*01:03/DPB1*02:01, HLA-DPA1*01:03/DPB1*04:01, HLA-<br>DPA1*02:01/DPB1*01:01, HLA-DPA1*02:01/DPB1*05:01, HLA-<br>DPA1*02:01/DPB1*14:01, HLA-DPA1*03:01/DPB1*04:02, HLA-<br>DQA1*01:01/DQB1*05:01, HLA-DQA1*01:02/DQB1*06:02, HLA-<br>DQA1*03:01/DQB1*03:02, HLA-DQA1*04:01/DQB1*04:02, HLA-<br>DQA1*05:01/DQB1*02:01, HLA-DQA1*05:01/DQB1*03:01, HLA-DRB1*01:01,<br>HLA-DRB1*03:01, HLA-DRB1*04:01, HLA-DRB1*04:05, HLA-DRB1*07:01,<br>HLA-DRB1*08:02, HLA-DRB1*09:01, HLA-DRB1*11:01, HLA-DRB1*12:01,<br>HLA-DRB1*13:02, HLA-DRB1*15:01, HLA-DRB3*01:01, HLA-DRB3*02:02,<br>HLA-DRB4*01:01, HLA-DRB5*01:01 |
| SAMRDQYMRTGEGFL | HLA-DPA1*01:03/DPB1*02:01, HLA-DPA1*01:03/DPB1*04:01, HLA-<br>DPA1*02:01/DPB1*01:01, HLA-DPA1*02:01/DPB1*05:01, HLA-<br>DPA1*02:01/DPB1*14:01, HLA-DPA1*03:01/DPB1*04:02, HLA-<br>DQA1*01:01/DQB1*05:01, HLA-DQA1*01:02/DQB1*06:02, HLA-<br>DQA1*03:01/DQB1*03:02, HLA-DQA1*04:01/DQB1*04:02, HLA-<br>DQA1*05:01/DQB1*02:01, HLA-DQA1*05:01/DQB1*03:01, HLA-DRB1*01:01,<br>HLA-DRB1*03:01, HLA-DRB1*04:01, HLA-DRB1*04:05, HLA-DRB1*07:01,<br>HLA-DRB1*08:02, HLA-DRB1*09:01, HLA-DRB1*11:01, HLA-DRB1*12:01,<br>HLA-DRB1*13:02, HLA-DRB1*15:01, HLA-DRB3*01:01, HLA-DRB3*02:02,<br>HLA-DRB4*01:01, HLA-DRB5*01:01 |
| SEDVPMVLVGNNCDL | HLA-DPA1*01:03/DPB1*02:01, HLA-DPA1*01:03/DPB1*04:01, HLA-<br>DPA1*02:01/DPB1*01:01, HLA-DPA1*02:01/DPB1*05:01, HLA-<br>DPA1*02:01/DPB1*14:01, HLA-DPA1*03:01/DPB1*04:02, HLA-<br>DQA1*01:01/DQB1*05:01, HLA-DQA1*01:02/DQB1*06:02, HLA-<br>DQA1*03:01/DQB1*03:02, HLA-DQA1*04:01/DQB1*04:02, HLA-<br>DQA1*05:01/DQB1*02:01, HLA-DQA1*05:01/DQB1*03:01, HLA-DRB1*01:01,<br>HLA-DRB1*03:01, HLA-DRB1*04:01, HLA-DRB1*04:05, HLA-DRB1*07:01,<br>HLA-DRB1*08:02, HLA-DRB1*09:01, HLA-DRB1*11:01, HLA-DRB1*12:01,<br>HLA-DRB1*13:02, HLA-DRB1*15:01, HLA-DRB3*01:01, HLA-DRB3*02:02,<br>HLA-DRB4*01:01, HLA-DRB5*01:01 |
| SKEEKTPGCVKIKKC | HLA-DPA1*01:03/DPB1*02:01, HLA-DPA1*01:03/DPB1*04:01, HLA-<br>DPA1*02:01/DPB1*01:01, HLA-DPA1*02:01/DPB1*05:01, HLA-<br>DPA1*02:01/DPB1*14:01, HLA-DPA1*03:01/DPB1*04:02, HLA-<br>DQA1*01:01/DQB1*05:01, HLA-DQA1*01:02/DQB1*06:02, HLA-<br>DQA1*03:01/DQB1*03:02, HLA-DQA1*04:01/DQB1*04:02, HLA-<br>DQA1*05:01/DQB1*02:01, HLA-DQA1*05:01/DQB1*03:01, HLA-DRB1*01:01,<br>HLA-DRB1*03:01, HLA-DRB1*04:01, HLA-DRB1*04:05, HLA-DRB1*07:01,<br>HLA-DRB1*08:02, HLA-DRB1*09:01, HLA-DRB1*11:01, HLA-DRB1*12:01,<br>HLA-DRB1*13:02, HLA-DRB1*15:01, HLA-DRB3*01:01, HLA-DRB3*02:02,<br>HLA-DRB4*01:01, HLA-DRB5*01:01 |
| SRTVDTKQAQDLARS | HLA-DPA1*01:03/DPB1*02:01, HLA-DPA1*01:03/DPB1*04:01, HLA-<br>DPA1*02:01/DPB1*01:01, HLA-DPA1*02:01/DPB1*05:01, HLA-<br>DPA1*02:01/DPB1*14:01, HLA-DPA1*03:01/DPB1*04:02, HLA-<br>DQA1*01:01/DQB1*05:01, HLA-DQA1*01:02/DQB1*06:02, HLA-<br>DQA1*03:01/DQB1*03:02, HLA-DQA1*04:01/DQB1*04:02, HLA-<br>DQA1*05:01/DQB1*02:01, HLA-DQA1*05:01/DQB1*03:01, HLA-DRB1*01:01,<br>HLA-DRB1*03:01, HLA-DRB1*04:01, HLA-DRB1*04:05, HLA-DRB1*07:01,<br>HLA-DRB1*08:02, HLA-DRB1*09:01, HLA-DRB1*11:01, HLA-DRB1*12:01,<br>HLA-DRB1*13:02, HLA-DRB1*15:01, HLA-DRB3*01:01, HLA-DRB3*02:02,<br>HLA-DRB4*01:01, HLA-DRB5*01:01 |
| SYGIPFIETSTKTRQ | HLA-DPA1*01:03/DPB1*02:01, HLA-DPA1*01:03/DPB1*04:01, HLA-<br>DPA1*02:01/DPB1*01:01, HLA-DPA1*02:01/DPB1*05:01, HLA-<br>DPA1*02:01/DPB1*14:01, HLA-DPA1*03:01/DPB1*04:02, HLA-<br>DQA1*01:01/DQB1*05:01, HLA-DQA1*01:02/DQB1*06:02, HLA-<br>DQA1*03:01/DQB1*03:02, HLA-DQA1*04:01/DQB1*04:02, HLA-<br>DQA1*05:01/DQB1*02:01, HLA-DQA1*05:01/DQB1*03:01, HLA-DRB1*01:01, |

| | |
|---|---|
| | HLA-DRB1*03:01, HLA-DRB1*04:01, HLA-DRB1*04:05, HLA-DRB1*07:01, HLA-DRB1*08:02, HLA-DRB1*09:01, HLA-DRB1*11:01, HLA-DRB1*12:01, HLA-DRB1*13:02, HLA-DRB1*15:01, HLA-DRB3*01:01, HLA-DRB3*02:02, HLA-DRB4*01:01, HLA-DRB5*01:01 |
| TDHEEYSAMRDQYMR | HLA-DPA1*01:03/DPB1*02:01, HLA-DPA1*01:03/DPB1*04:01, HLA-DPA1*02:01/DPB1*01:01, HLA-DPA1*02:01/DPB1*05:01, HLA-DPA1*02:01/DPB1*14:01, HLA-DPA1*03:01/DPB1*04:02, HLA-DQA1*01:01/DQB1*05:01, HLA-DQA1*01:02/DQB1*06:02, HLA-DQA1*03:01/DQB1*03:02, HLA-DQA1*04:01/DQB1*04:02, HLA-DQA1*05:01/DQB1*02:01, HLA-DQA1*05:01/DQB1*03:01, HLA-DRB1*01:01, HLA-DRB1*03:01, HLA-DRB1*04:01, HLA-DRB1*04:05, HLA-DRB1*07:01, HLA-DRB1*08:02, HLA-DRB1*09:01, HLA-DRB1*11:01, HLA-DRB1*12:01, HLA-DRB1*13:02, HLA-DRB1*15:01, HLA-DRB3*01:01, HLA-DRB3*02:02, HLA-DRB4*01:01, HLA-DRB5*01:01 |
| TEYKLVVVGADDVGK | HLA-DPA1*01:03/DPB1*02:01, HLA-DPA1*01:03/DPB1*04:01, HLA-DPA1*02:01/DPB1*01:01, HLA-DPA1*02:01/DPB1*05:01, HLA-DPA1*02:01/DPB1*14:01, HLA-DPA1*03:01/DPB1*04:02, HLA-DQA1*01:01/DQB1*05:01, HLA-DQA1*01:02/DQB1*06:02, HLA-DQA1*03:01/DQB1*03:02, HLA-DQA1*04:01/DQB1*04:02, HLA-DQA1*05:01/DQB1*02:01, HLA-DQA1*05:01/DQB1*03:01, HLA-DRB1*01:01, HLA-DRB1*03:01, HLA-DRB1*04:01, HLA-DRB1*04:05, HLA-DRB1*07:01, HLA-DRB1*08:02, HLA-DRB1*09:01, HLA-DRB1*11:01, HLA-DRB1*12:01, HLA-DRB1*13:02, HLA-DRB1*15:01, HLA-DRB3*01:01, HLA-DRB3*02:02, HLA-DRB4*01:01, HLA-DRB5*01:01 |
| TGEGFLCVFAINNTK | HLA-DPA1*01:03/DPB1*02:01, HLA-DPA1*01:03/DPB1*04:01, HLA-DPA1*02:01/DPB1*01:01, HLA-DPA1*02:01/DPB1*05:01, HLA-DPA1*02:01/DPB1*14:01, HLA-DPA1*03:01/DPB1*04:02, HLA-DQA1*01:01/DQB1*05:01, HLA-DQA1*01:02/DQB1*06:02, HLA-DQA1*03:01/DQB1*03:02, HLA-DQA1*04:01/DQB1*04:02, HLA-DQA1*05:01/DQB1*02:01, HLA-DQA1*05:01/DQB1*03:01, HLA-DRB1*01:01, HLA-DRB1*03:01, HLA-DRB1*04:01, HLA-DRB1*04:05, HLA-DRB1*07:01, HLA-DRB1*08:02, HLA-DRB1*09:01, HLA-DRB1*11:01, HLA-DRB1*12:01, HLA-DRB1*13:02, HLA-DRB1*15:01, HLA-DRB3*01:01, HLA-DRB3*02:02, HLA-DRB4*01:01, HLA-DRB5*01:01 |
| TIEDSYRKQVVIDGE | HLA-DPA1*01:03/DPB1*02:01, HLA-DPA1*01:03/DPB1*04:01, HLA-DPA1*02:01/DPB1*01:01, HLA-DPA1*02:01/DPB1*05:01, HLA-DPA1*02:01/DPB1*14:01, HLA-DPA1*03:01/DPB1*04:02, HLA-DQA1*01:01/DQB1*05:01, HLA-DQA1*01:02/DQB1*06:02, HLA-DQA1*03:01/DQB1*03:02, HLA-DQA1*04:01/DQB1*04:02, HLA-DQA1*05:01/DQB1*02:01, HLA-DQA1*05:01/DQB1*03:01, HLA-DRB1*01:01, HLA-DRB1*03:01, HLA-DRB1*04:01, HLA-DRB1*04:05, HLA-DRB1*07:01, HLA-DRB1*08:02, HLA-DRB1*09:01, HLA-DRB1*11:01, HLA-DRB1*12:01, HLA-DRB1*13:02, HLA-DRB1*15:01, HLA-DRB3*01:01, HLA-DRB3*02:02, HLA-DRB4*01:01, HLA-DRB5*01:01 |
| TIQLIQNHFVDEYDP | HLA-DPA1*01:03/DPB1*02:01, HLA-DPA1*01:03/DPB1*04:01, HLA-DPA1*02:01/DPB1*01:01, HLA-DPA1*02:01/DPB1*05:01, HLA-DPA1*02:01/DPB1*14:01, HLA-DPA1*03:01/DPB1*04:02, HLA-DQA1*01:01/DQB1*05:01, HLA-DQA1*01:02/DQB1*06:02, HLA-DQA1*03:01/DQB1*03:02, HLA-DQA1*04:01/DQB1*04:02, HLA-DQA1*05:01/DQB1*02:01, HLA-DQA1*05:01/DQB1*03:01, HLA-DRB1*01:01, HLA-DRB1*03:01, HLA-DRB1*04:01, HLA-DRB1*04:05, HLA-DRB1*07:01, HLA-DRB1*08:02, HLA-DRB1*09:01, HLA-DRB1*11:01, HLA-DRB1*12:01, HLA-DRB1*13:02, HLA-DRB1*15:01, HLA-DRB3*01:01, HLA-DRB3*02:02, HLA-DRB4*01:01, HLA-DRB5*01:01 |
| TKQAQDLARSYGIPF | HLA-DPA1*01:03/DPB1*02:01, HLA-DPA1*01:03/DPB1*04:01, HLA-DPA1*02:01/DPB1*01:01, HLA-DPA1*02:01/DPB1*05:01, HLA-DPA1*02:01/DPB1*14:01, HLA-DPA1*03:01/DPB1*04:02, HLA-DQA1*01:01/DQB1*05:01, HLA-DQA1*01:02/DQB1*06:02, HLA-DQA1*03:01/DQB1*03:02, HLA-DQA1*04:01/DQB1*04:02, HLA-DQA1*05:01/DQB1*02:01, HLA-DQA1*05:01/DQB1*03:01, HLA-DRB1*01:01, |

| | |
|---|---|
| | HLA-DRB1*03:01, HLA-DRB1*04:01, HLA-DRB1*04:05, HLA-DRB1*07:01, HLA-DRB1*08:02, HLA-DRB1*09:01, HLA-DRB1*11:01, HLA-DRB1*12:01, HLA-DRB1*13:02, HLA-DRB1*15:01, HLA-DRB3*01:01, HLA-DRB3*02:02, HLA-DRB4*01:01, HLA-DRB5*01:01 |
| TLYREIRQYFIKKIS | HLA-DPA1*01:03/DPB1*02:01, HLA-DPA1*01:03/DPB1*04:01, HLA-DPA1*02:01/DPB1*01:01, HLA-DPA1*02:01/DPB1*05:01, HLA-DPA1*02:01/DPB1*14:01, HLA-DPA1*03:01/DPB1*04:02, HLA-DQA1*01:01/DQB1*05:01, HLA-DQA1*01:02/DQB1*06:02, HLA-DQA1*03:01/DQB1*03:02, HLA-DQA1*04:01/DQB1*04:02, HLA-DQA1*05:01/DQB1*02:01, HLA-DQA1*05:01/DQB1*03:01, HLA-DRB1*01:01, HLA-DRB1*03:01, HLA-DRB1*04:01, HLA-DRB1*04:05, HLA-DRB1*07:01, HLA-DRB1*08:02, HLA-DRB1*09:01, HLA-DRB1*11:01, HLA-DRB1*12:01, HLA-DRB1*13:02, HLA-DRB1*15:01, HLA-DRB3*01:01, HLA-DRB3*02:02, HLA-DRB4*01:01, HLA-DRB5*01:01 |
| TTDHEEYSAMRDQYM | HLA-DPA1*01:03/DPB1*02:01, HLA-DPA1*01:03/DPB1*04:01, HLA-DPA1*02:01/DPB1*01:01, HLA-DPA1*02:01/DPB1*05:01, HLA-DPA1*02:01/DPB1*14:01, HLA-DPA1*03:01/DPB1*04:02, HLA-DQA1*01:01/DQB1*05:01, HLA-DQA1*01:02/DQB1*06:02, HLA-DQA1*03:01/DQB1*03:02, HLA-DQA1*04:01/DQB1*04:02, HLA-DQA1*05:01/DQB1*02:01, HLA-DQA1*05:01/DQB1*03:01, HLA-DRB1*01:01, HLA-DRB1*03:01, HLA-DRB1*04:01, HLA-DRB1*04:05, HLA-DRB1*07:01, HLA-DRB1*08:02, HLA-DRB1*09:01, HLA-DRB1*11:01, HLA-DRB1*12:01, HLA-DRB1*13:02, HLA-DRB1*15:01, HLA-DRB3*01:01, HLA-DRB3*02:02, HLA-DRB4*01:01, HLA-DRB5*01:01 |
| TVDTKQAQDLARSYG | HLA-DPA1*01:03/DPB1*02:01, HLA-DPA1*01:03/DPB1*04:01, HLA-DPA1*02:01/DPB1*01:01, HLA-DPA1*02:01/DPB1*05:01, HLA-DPA1*02:01/DPB1*14:01, HLA-DPA1*03:01/DPB1*04:02, HLA-DQA1*01:01/DQB1*05:01, HLA-DQA1*01:02/DQB1*06:02, HLA-DQA1*03:01/DQB1*03:02, HLA-DQA1*04:01/DQB1*04:02, HLA-DQA1*05:01/DQB1*02:01, HLA-DQA1*05:01/DQB1*03:01, HLA-DRB1*01:01, HLA-DRB1*03:01, HLA-DRB1*04:01, HLA-DRB1*04:05, HLA-DRB1*07:01, HLA-DRB1*08:02, HLA-DRB1*09:01, HLA-DRB1*11:01, HLA-DRB1*12:01, HLA-DRB1*13:02, HLA-DRB1*15:01, HLA-DRB3*01:01, HLA-DRB3*02:02, HLA-DRB4*01:01, HLA-DRB5*01:01 |
| VDEYDPTIEDSYRKQ | HLA-DPA1*01:03/DPB1*02:01, HLA-DPA1*01:03/DPB1*04:01, HLA-DPA1*02:01/DPB1*01:01, HLA-DPA1*02:01/DPB1*05:01, HLA-DPA1*02:01/DPB1*14:01, HLA-DPA1*03:01/DPB1*04:02, HLA-DQA1*01:01/DQB1*05:01, HLA-DQA1*01:02/DQB1*06:02, HLA-DQA1*03:01/DQB1*03:02, HLA-DQA1*04:01/DQB1*04:02, HLA-DQA1*05:01/DQB1*02:01, HLA-DQA1*05:01/DQB1*03:01, HLA-DRB1*01:01, HLA-DRB1*03:01, HLA-DRB1*04:01, HLA-DRB1*04:05, HLA-DRB1*07:01, HLA-DRB1*08:02, HLA-DRB1*09:01, HLA-DRB1*11:01, HLA-DRB1*12:01, HLA-DRB1*13:02, HLA-DRB1*15:01, HLA-DRB3*01:01, HLA-DRB3*02:02, HLA-DRB4*01:01, HLA-DRB5*01:01 |
| VDTKQAQDLARSYGI | HLA-DPA1*01:03/DPB1*02:01, HLA-DPA1*01:03/DPB1*04:01, HLA-DPA1*02:01/DPB1*01:01, HLA-DPA1*02:01/DPB1*05:01, HLA-DPA1*02:01/DPB1*14:01, HLA-DPA1*03:01/DPB1*04:02, HLA-DQA1*01:01/DQB1*05:01, HLA-DQA1*01:02/DQB1*06:02, HLA-DQA1*03:01/DQB1*03:02, HLA-DQA1*04:01/DQB1*04:02, HLA-DQA1*05:01/DQB1*02:01, HLA-DQA1*05:01/DQB1*03:01, HLA-DRB1*01:01, HLA-DRB1*03:01, HLA-DRB1*04:01, HLA-DRB1*04:05, HLA-DRB1*07:01, HLA-DRB1*08:02, HLA-DRB1*09:01, HLA-DRB1*11:01, HLA-DRB1*12:01, HLA-DRB1*13:02, HLA-DRB1*15:01, HLA-DRB3*01:01, HLA-DRB3*02:02, HLA-DRB4*01:01, HLA-DRB5*01:01 |
| VEDAFYTLYREIRQY | HLA-DPA1*01:03/DPB1*02:01, HLA-DPA1*01:03/DPB1*04:01, HLA-DPA1*02:01/DPB1*01:01, HLA-DPA1*02:01/DPB1*05:01, HLA-DPA1*02:01/DPB1*14:01, HLA-DPA1*03:01/DPB1*04:02, HLA-DQA1*01:01/DQB1*05:01, HLA-DQA1*01:02/DQB1*06:02, HLA-DQA1*03:01/DQB1*03:02, HLA-DQA1*04:01/DQB1*04:02, HLA-DQA1*05:01/DQB1*02:01, HLA-DQA1*05:01/DQB1*03:01, HLA-DRB1*01:01, |

| | |
|---|---|
| | HLA-DRB1*03:01, HLA-DRB1*04:01, HLA-DRB1*04:05, HLA-DRB1*07:01, HLA-DRB1*08:02, HLA-DRB1*09:01, HLA-DRB1*11:01, HLA-DRB1*12:01, HLA-DRB1*13:02, HLA-DRB1*15:01, HLA-DRB3*01:01, HLA-DRB3*02:02, HLA-DRB4*01:01, HLA-DRB5*01:01 |
| VFAINNTKSFEDIHH | HLA-DPA1*01:03/DPB1*02:01, HLA-DPA1*01:03/DPB1*04:01, HLA-DPA1*02:01/DPB1*01:01, HLA-DPA1*02:01/DPB1*05:01, HLA-DPA1*02:01/DPB1*14:01, HLA-DPA1*03:01/DPB1*04:02, HLA-DQA1*01:01/DQB1*05:01, HLA-DQA1*01:02/DQB1*06:02, HLA-DQA1*03:01/DQB1*03:02, HLA-DQA1*04:01/DQB1*04:02, HLA-DQA1*05:01/DQB1*02:01, HLA-DQA1*05:01/DQB1*03:01, HLA-DRB1*01:01, HLA-DRB1*03:01, HLA-DRB1*04:01, HLA-DRB1*04:05, HLA-DRB1*07:01, HLA-DRB1*08:02, HLA-DRB1*09:01, HLA-DRB1*11:01, HLA-DRB1*12:01, HLA-DRB1*13:02, HLA-DRB1*15:01, HLA-DRB3*01:01, HLA-DRB3*02:02, HLA-DRB4*01:01, HLA-DRB5*01:01 |
| VGADDVGKSAFTIQL | HLA-DPA1*01:03/DPB1*02:01, HLA-DPA1*01:03/DPB1*04:01, HLA-DPA1*02:01/DPB1*01:01, HLA-DPA1*02:01/DPB1*05:01, HLA-DPA1*02:01/DPB1*14:01, HLA-DPA1*03:01/DPB1*04:02, HLA-DQA1*01:01/DQB1*05:01, HLA-DQA1*01:02/DQB1*06:02, HLA-DQA1*03:01/DQB1*03:02, HLA-DQA1*04:01/DQB1*04:02, HLA-DQA1*05:01/DQB1*02:01, HLA-DQA1*05:01/DQB1*03:01, HLA-DRB1*01:01, HLA-DRB1*03:01, HLA-DRB1*04:01, HLA-DRB1*04:05, HLA-DRB1*07:01, HLA-DRB1*08:02, HLA-DRB1*09:01, HLA-DRB1*11:01, HLA-DRB1*12:01, HLA-DRB1*13:02, HLA-DRB1*15:01, HLA-DRB3*01:01, HLA-DRB3*02:02, HLA-DRB4*01:01, HLA-DRB5*01:01 |
| VGKSAFTIQLIQNHF | HLA-DPA1*01:03/DPB1*02:01, HLA-DPA1*01:03/DPB1*04:01, HLA-DPA1*02:01/DPB1*01:01, HLA-DPA1*02:01/DPB1*05:01, HLA-DPA1*02:01/DPB1*14:01, HLA-DPA1*03:01/DPB1*04:02, HLA-DQA1*01:01/DQB1*05:01, HLA-DQA1*01:02/DQB1*06:02, HLA-DQA1*03:01/DQB1*03:02, HLA-DQA1*04:01/DQB1*04:02, HLA-DQA1*05:01/DQB1*02:01, HLA-DQA1*05:01/DQB1*03:01, HLA-DRB1*01:01, HLA-DRB1*03:01, HLA-DRB1*04:01, HLA-DRB1*04:05, HLA-DRB1*07:01, HLA-DRB1*08:02, HLA-DRB1*09:01, HLA-DRB1*11:01, HLA-DRB1*12:01, HLA-DRB1*13:02, HLA-DRB1*15:01, HLA-DRB3*01:01, HLA-DRB3*02:02, HLA-DRB4*01:01, HLA-DRB5*01:01 |
| VGNNCDLPSRTVDTK | HLA-DPA1*01:03/DPB1*02:01, HLA-DPA1*01:03/DPB1*04:01, HLA-DPA1*02:01/DPB1*01:01, HLA-DPA1*02:01/DPB1*05:01, HLA-DPA1*02:01/DPB1*14:01, HLA-DPA1*03:01/DPB1*04:02, HLA-DQA1*01:01/DQB1*05:01, HLA-DQA1*01:02/DQB1*06:02, HLA-DQA1*03:01/DQB1*03:02, HLA-DQA1*04:01/DQB1*04:02, HLA-DQA1*05:01/DQB1*02:01, HLA-DQA1*05:01/DQB1*03:01, HLA-DRB1*01:01, HLA-DRB1*03:01, HLA-DRB1*04:01, HLA-DRB1*04:05, HLA-DRB1*07:01, HLA-DRB1*08:02, HLA-DRB1*09:01, HLA-DRB1*11:01, HLA-DRB1*12:01, HLA-DRB1*13:02, HLA-DRB1*15:01, HLA-DRB3*01:01, HLA-DRB3*02:02, HLA-DRB4*01:01, HLA-DRB5*01:01 |
| VKDSEDVPMVLVGNN | HLA-DPA1*01:03/DPB1*02:01, HLA-DPA1*01:03/DPB1*04:01, HLA-DPA1*02:01/DPB1*01:01, HLA-DPA1*02:01/DPB1*05:01, HLA-DPA1*02:01/DPB1*14:01, HLA-DPA1*03:01/DPB1*04:02, HLA-DQA1*01:01/DQB1*05:01, HLA-DQA1*01:02/DQB1*06:02, HLA-DQA1*03:01/DQB1*03:02, HLA-DQA1*04:01/DQB1*04:02, HLA-DQA1*05:01/DQB1*02:01, HLA-DQA1*05:01/DQB1*03:01, HLA-DRB1*01:01, HLA-DRB1*03:01, HLA-DRB1*04:01, HLA-DRB1*04:05, HLA-DRB1*07:01, HLA-DRB1*08:02, HLA-DRB1*09:01, HLA-DRB1*11:01, HLA-DRB1*12:01, HLA-DRB1*13:02, HLA-DRB1*15:01, HLA-DRB3*01:01, HLA-DRB3*02:02, HLA-DRB4*01:01, HLA-DRB5*01:01 |
| VLVGNNCDLPSRTVD | HLA-DPA1*01:03/DPB1*02:01, HLA-DPA1*01:03/DPB1*04:01, HLA-DPA1*02:01/DPB1*01:01, HLA-DPA1*02:01/DPB1*05:01, HLA-DPA1*02:01/DPB1*14:01, HLA-DPA1*03:01/DPB1*04:02, HLA-DQA1*01:01/DQB1*05:01, HLA-DQA1*01:02/DQB1*06:02, HLA-DQA1*03:01/DQB1*03:02, HLA-DQA1*04:01/DQB1*04:02, HLA-DQA1*05:01/DQB1*02:01, HLA-DQA1*05:01/DQB1*03:01, HLA-DRB1*01:01, |

| | |
|---|---|
| | HLA-DRB1*03:01, HLA-DRB1*04:01, HLA-DRB1*04:05, HLA-DRB1*07:01, HLA-DRB1*08:02, HLA-DRB1*09:01, HLA-DRB1*11:01, HLA-DRB1*12:01, HLA-DRB1*13:02, HLA-DRB1*15:01, HLA-DRB3*01:01, HLA-DRB3*02:02, HLA-DRB4*01:01, HLA-DRB5*01:01 |
| VPMVLVGNNCDLPSR | HLA-DPA1*01:03/DPB1*02:01, HLA-DPA1*01:03/DPB1*04:01, HLA-DPA1*02:01/DPB1*01:01, HLA-DPA1*02:01/DPB1*05:01, HLA-DPA1*02:01/DPB1*14:01, HLA-DPA1*03:01/DPB1*04:02, HLA-DQA1*01:01/DQB1*05:01, HLA-DQA1*01:02/DQB1*06:02, HLA-DQA1*03:01/DQB1*03:02, HLA-DQA1*04:01/DQB1*04:02, HLA-DQA1*05:01/DQB1*02:01, HLA-DQA1*05:01/DQB1*03:01, HLA-DRB1*01:01, HLA-DRB1*03:01, HLA-DRB1*04:01, HLA-DRB1*04:05, HLA-DRB1*07:01, HLA-DRB1*08:02, HLA-DRB1*09:01, HLA-DRB1*11:01, HLA-DRB1*12:01, HLA-DRB1*13:02, HLA-DRB1*15:01, HLA-DRB3*01:01, HLA-DRB3*02:02, HLA-DRB4*01:01, HLA-DRB5*01:01 |
| VVGADDVGKSAFTIQ | HLA-DPA1*01:03/DPB1*02:01, HLA-DPA1*01:03/DPB1*04:01, HLA-DPA1*02:01/DPB1*01:01, HLA-DPA1*02:01/DPB1*05:01, HLA-DPA1*02:01/DPB1*14:01, HLA-DPA1*03:01/DPB1*04:02, HLA-DQA1*01:01/DQB1*05:01, HLA-DQA1*01:02/DQB1*06:02, HLA-DQA1*03:01/DQB1*03:02, HLA-DQA1*04:01/DQB1*04:02, HLA-DQA1*05:01/DQB1*02:01, HLA-DQA1*05:01/DQB1*03:01, HLA-DRB1*01:01, HLA-DRB1*03:01, HLA-DRB1*04:01, HLA-DRB1*04:05, HLA-DRB1*07:01, HLA-DRB1*08:02, HLA-DRB1*09:01, HLA-DRB1*11:01, HLA-DRB1*12:01, HLA-DRB1*13:02, HLA-DRB1*15:01, HLA-DRB3*01:01, HLA-DRB3*02:02, HLA-DRB4*01:01, HLA-DRB5*01:01 |
| VVVGADDVGKSAFTI | HLA-DPA1*01:03/DPB1*02:01, HLA-DPA1*01:03/DPB1*04:01, HLA-DPA1*02:01/DPB1*01:01, HLA-DPA1*02:01/DPB1*05:01, HLA-DPA1*02:01/DPB1*14:01, HLA-DPA1*03:01/DPB1*04:02, HLA-DQA1*01:01/DQB1*05:01, HLA-DQA1*01:02/DQB1*06:02, HLA-DQA1*03:01/DQB1*03:02, HLA-DQA1*04:01/DQB1*04:02, HLA-DQA1*05:01/DQB1*02:01, HLA-DQA1*05:01/DQB1*03:01, HLA-DRB1*01:01, HLA-DRB1*03:01, HLA-DRB1*04:01, HLA-DRB1*04:05, HLA-DRB1*07:01, HLA-DRB1*08:02, HLA-DRB1*09:01, HLA-DRB1*11:01, HLA-DRB1*12:01, HLA-DRB1*13:02, HLA-DRB1*15:01, HLA-DRB3*01:01, HLA-DRB3*02:02, HLA-DRB4*01:01, HLA-DRB5*01:01 |
| YDPTIEDSYRKQVVI | HLA-DPA1*01:03/DPB1*02:01, HLA-DPA1*01:03/DPB1*04:01, HLA-DPA1*02:01/DPB1*01:01, HLA-DPA1*02:01/DPB1*05:01, HLA-DPA1*02:01/DPB1*14:01, HLA-DPA1*03:01/DPB1*04:02, HLA-DQA1*01:01/DQB1*05:01, HLA-DQA1*01:02/DQB1*06:02, HLA-DQA1*03:01/DQB1*03:02, HLA-DQA1*04:01/DQB1*04:02, HLA-DQA1*05:01/DQB1*02:01, HLA-DQA1*05:01/DQB1*03:01, HLA-DRB1*01:01, HLA-DRB1*03:01, HLA-DRB1*04:01, HLA-DRB1*04:05, HLA-DRB1*07:01, HLA-DRB1*08:02, HLA-DRB1*09:01, HLA-DRB1*11:01, HLA-DRB1*12:01, HLA-DRB1*13:02, HLA-DRB1*15:01, HLA-DRB3*01:01, HLA-DRB3*02:02, HLA-DRB4*01:01, HLA-DRB5*01:01 |
| YFIKKISKEEKTPGC | HLA-DPA1*01:03/DPB1*02:01, HLA-DPA1*01:03/DPB1*04:01, HLA-DPA1*02:01/DPB1*01:01, HLA-DPA1*02:01/DPB1*05:01, HLA-DPA1*02:01/DPB1*14:01, HLA-DPA1*03:01/DPB1*04:02, HLA-DQA1*01:01/DQB1*05:01, HLA-DQA1*01:02/DQB1*06:02, HLA-DQA1*03:01/DQB1*03:02, HLA-DQA1*04:01/DQB1*04:02, HLA-DQA1*05:01/DQB1*02:01, HLA-DQA1*05:01/DQB1*03:01, HLA-DRB1*01:01, HLA-DRB1*03:01, HLA-DRB1*04:01, HLA-DRB1*04:05, HLA-DRB1*07:01, HLA-DRB1*08:02, HLA-DRB1*09:01, HLA-DRB1*11:01, HLA-DRB1*12:01, HLA-DRB1*13:02, HLA-DRB1*15:01, HLA-DRB3*01:01, HLA-DRB3*02:02, HLA-DRB4*01:01, HLA-DRB5*01:01 |
| YKLVVVGADDVGKSA | HLA-DPA1*01:03/DPB1*02:01, HLA-DPA1*01:03/DPB1*04:01, HLA-DPA1*02:01/DPB1*01:01, HLA-DPA1*02:01/DPB1*05:01, HLA-DPA1*02:01/DPB1*14:01, HLA-DPA1*03:01/DPB1*04:02, HLA-DQA1*01:01/DQB1*05:01, HLA-DQA1*01:02/DQB1*06:02, HLA-DQA1*03:01/DQB1*03:02, HLA-DQA1*04:01/DQB1*04:02, HLA-DQA1*05:01/DQB1*02:01, HLA-DQA1*05:01/DQB1*03:01, HLA-DRB1*01:01, |

| | |
|---|---|
| | HLA-DRB1*03:01, HLA-DRB1*04:01, HLA-DRB1*04:05, HLA-DRB1*07:01, HLA-DRB1*08:02, HLA-DRB1*09:01, HLA-DRB1*11:01, HLA-DRB1*12:01, HLA-DRB1*13:02, HLA-DRB1*15:01, HLA-DRB3*01:01, HLA-DRB3*02:02, HLA-DRB4*01:01, HLA-DRB5*01:01 |
| YMRTGEGFLCVFAIN | HLA-DPA1*01:03/DPB1*02:01, HLA-DPA1*01:03/DPB1*04:01, HLA-DPA1*02:01/DPB1*01:01, HLA-DPA1*02:01/DPB1*05:01, HLA-DPA1*02:01/DPB1*14:01, HLA-DPA1*03:01/DPB1*04:02, HLA-DQA1*01:01/DQB1*05:01, HLA-DQA1*01:02/DQB1*06:02, HLA-DQA1*03:01/DQB1*03:02, HLA-DQA1*04:01/DQB1*04:02, HLA-DQA1*05:01/DQB1*02:01, HLA-DQA1*05:01/DQB1*03:01, HLA-DRB1*01:01, HLA-DRB1*03:01, HLA-DRB1*04:01, HLA-DRB1*04:05, HLA-DRB1*07:01, HLA-DRB1*08:02, HLA-DRB1*09:01, HLA-DRB1*11:01, HLA-DRB1*12:01, HLA-DRB1*13:02, HLA-DRB1*15:01, HLA-DRB3*01:01, HLA-DRB3*02:02, HLA-DRB4*01:01, HLA-DRB5*01:01 |
| YREIRQYFIKKISKE | HLA-DPA1*01:03/DPB1*02:01, HLA-DPA1*01:03/DPB1*04:01, HLA-DPA1*02:01/DPB1*01:01, HLA-DPA1*02:01/DPB1*05:01, HLA-DPA1*02:01/DPB1*14:01, HLA-DPA1*03:01/DPB1*04:02, HLA-DQA1*01:01/DQB1*05:01, HLA-DQA1*01:02/DQB1*06:02, HLA-DQA1*03:01/DQB1*03:02, HLA-DQA1*04:01/DQB1*04:02, HLA-DQA1*05:01/DQB1*02:01, HLA-DQA1*05:01/DQB1*03:01, HLA-DRB1*01:01, HLA-DRB1*03:01, HLA-DRB1*04:01, HLA-DRB1*04:05, HLA-DRB1*07:01, HLA-DRB1*08:02, HLA-DRB1*09:01, HLA-DRB1*11:01, HLA-DRB1*12:01, HLA-DRB1*13:02, HLA-DRB1*15:01, HLA-DRB3*01:01, HLA-DRB3*02:02, HLA-DRB4*01:01, HLA-DRB5*01:01 |
| YSAMRDQYMRTGEGF | HLA-DPA1*01:03/DPB1*02:01, HLA-DPA1*01:03/DPB1*04:01, HLA-DPA1*02:01/DPB1*01:01, HLA-DPA1*02:01/DPB1*05:01, HLA-DPA1*02:01/DPB1*14:01, HLA-DPA1*03:01/DPB1*04:02, HLA-DQA1*01:01/DQB1*05:01, HLA-DQA1*01:02/DQB1*06:02, HLA-DQA1*03:01/DQB1*03:02, HLA-DQA1*04:01/DQB1*04:02, HLA-DQA1*05:01/DQB1*02:01, HLA-DQA1*05:01/DQB1*03:01, HLA-DRB1*01:01, HLA-DRB1*03:01, HLA-DRB1*04:01, HLA-DRB1*04:05, HLA-DRB1*07:01, HLA-DRB1*08:02, HLA-DRB1*09:01, HLA-DRB1*11:01, HLA-DRB1*12:01, HLA-DRB1*13:02, HLA-DRB1*15:01, HLA-DRB3*01:01, HLA-DRB3*02:02, HLA-DRB4*01:01, HLA-DRB5*01:01 |
| YTLYREIRQYFIKKI | HLA-DPA1*01:03/DPB1*02:01, HLA-DPA1*01:03/DPB1*04:01, HLA-DPA1*02:01/DPB1*01:01, HLA-DPA1*02:01/DPB1*05:01, HLA-DPA1*02:01/DPB1*14:01, HLA-DPA1*03:01/DPB1*04:02, HLA-DQA1*01:01/DQB1*05:01, HLA-DQA1*01:02/DQB1*06:02, HLA-DQA1*03:01/DQB1*03:02, HLA-DQA1*04:01/DQB1*04:02, HLA-DQA1*05:01/DQB1*02:01, HLA-DQA1*05:01/DQB1*03:01, HLA-DRB1*01:01, HLA-DRB1*03:01, HLA-DRB1*04:01, HLA-DRB1*04:05, HLA-DRB1*07:01, HLA-DRB1*08:02, HLA-DRB1*09:01, HLA-DRB1*11:01, HLA-DRB1*12:01, HLA-DRB1*13:02, HLA-DRB1*15:01, HLA-DRB3*01:01, HLA-DRB3*02:02, HLA-DRB4*01:01, HLA-DRB5*01:01 |

**Supplementary Table 2:** The predicted HTL epitope of pan-ras mRNA cancer vaccine (WO 2022/081764 A1 (PCT/US 2021/054859) with their corresponding Alleles as predicted by NetMHCpan v4.0 tools.

| #Acceptor | Donor H | Donor | Frames | Frac | Avg Dist | Avg Ang |
|---|---|---|---|---|---|---|
| RX8_1545@N1 | LYS_406@HZ3 | LYS_406@NZ | 3216 | 0.0643 | 2.8977 | 158.9601 |
| RX8_1545@N1 | LYS_406@HZ2 | LYS_406@NZ | 3153 | 0.0631 | 2.8974 | 158.7939 |
| RX8_1545@N1 | LYS_406@HZ1 | LYS_406@NZ | 2915 | 0.0583 | 2.8986 | 158.9627 |
| RX8_1545@N | LYS_406@HZ3 | LYS_406@NZ | 831 | 0.0166 | 2.9077 | 151.8168 |
| RX8_1545@N | LYS_406@HZ2 | LYS_406@NZ | 801 | 0.016 | 2.9125 | 152.3035 |
| RX8_1545@N | LYS_406@HZ1 | LYS_406@NZ | 706 | 0.0141 | 2.9094 | 151.5168 |
| RX8_1545@N2 | GLN_328@HE22 | GLN_328@NE2 | 549 | 0.011 | 2.9356 | 161.5525 |
| RX8_1545@N2 | TYR_238@HH | TYR_238@OH | 394 | 0.0079 | 2.8572 | 159.799 |
| RX8_1545@H22 | PRO_409@HA | PRO_409@CA | 378 | 0.0076 | 2.934 | 143.2209 |
| RX8_1545@H22 | LYS_406@HE2 | LYS_406@CE | 349 | 0.007 | 2.9141 | 146.8488 |
| RX8_1545@H17 | LYS_406@HZ3 | LYS_406@NZ | 310 | 0.0062 | 2.881 | 144.0158 |
| RX8_1545@H17 | LYS_406@HZ2 | LYS_406@NZ | 304 | 0.0061 | 2.8827 | 144.5113 |
| RX8_1545@H21 | GLU_1184@H | GLU_1184@N | 275 | 0.0055 | 2.8313 | 147.0995 |
| RX8_1545@H17 | LYS_406@HZ1 | LYS_406@NZ | 208 | 0.0042 | 2.8904 | 144.666 |
| RX8_1545@H18 | LYS_406@HZ2 | LYS_406@NZ | 169 | 0.0034 | 2.8836 | 144.7307 |

**Supplementary Table 3:** The list of Average distance (Å) and Average bond angle ($^0$) for the donor (resiquimod) and receptors (TLR7–pan-RAS mRNA vaccine) complexes. RX8 represents resiquimod.

| #Acceptor | Donor H | Donor | Frames | Frac | Avg Dist | Avg Ang |
|---|---|---|---|---|---|---|
| RX8_609@O1 | TYR_196@HH | TYR_196@OH | 738 | 0.0148 | 2.8111 | 162.0569 |
| RX8_609@N1 | ARG_41@HH11 | ARG_41@NH1 | 661 | 0.0132 | 2.9075 | 158.6441 |
| RX8_609@N1 | GLN_311@HE22 | GLN_311@NE2 | 519 | 0.0104 | 2.9 | 161.164 |
| RX8_609@N1 | GLN_311@HE21 | GLN_311@NE2 | 370 | 0.0074 | 2.9059 | 158.0504 |
| RX8_609@N | ARG_41@HH11 | ARG_41@NH1 | 256 | 0.0051 | 2.9056 | 152.7814 |
| RX8_609@N1 | SER_287@HG | SER_287@OG | 233 | 0.0047 | 2.7849 | 163.2786 |
| RX8_609@N2 | SER_39@HG | SER_39@OG | 209 | 0.0042 | 2.8552 | 160.4176 |
| RX8_609@H13 | ASP_30@HB3 | ASP_30@CB | 205 | 0.0041 | 2.9333 | 143.6333 |
| RX8_609@O1 | ARG_474@HH21 | ARG_474@NH2 | 106 | 0.0021 | 2.8987 | 151.5914 |
| RX8_609@H1 | ARG_474@HE | ARG_474@NE | 104 | 0.0021 | 2.8917 | 149.2019 |
| RX8_609@N1 | THR_87@HG1 | THR_87@OG1 | 79 | 0.0016 | 2.8516 | 164.2773 |
| RX8_609@H9 | PHE_453@HZ | PHE_453@CZ | 77 | 0.0015 | 2.9296 | 142.5141 |
| RX8_609@N1 | ARG_390@HH21 | ARG_390@NH2 | 76 | 0.0015 | 2.9205 | 149.2174 |
| RX8_609@N1 | HIE_455@H | HIE_455@N | 74 | 0.0015 | 2.9261 | 159.7856 |
| RX8_609@O1 | ASN_85@HD21 | ASN_85@ND2 | 68 | 0.0014 | 2.887 | 158.1256 |

**Supplementary Table 4:** The list of Average distance (Å) and Average bond angle ($^0$) for the donor (resiquimod) and receptors (TLR7–pan-RAS mRNA vaccine) complexes. RX8 represents resiquimod.

Click the[±lto see the duster contents

| | Cluster Composition | Cluster Ancestor | Cluster Representative Sequence | Max Score | Total Score | Query Cover | E value | Per. Ident | Acc. Len | Accession |
|---|---|---|---|---|---|---|---|---|---|---|
| � | 1±1243 member(s), 210 organism(s) | !221 | GTPase KRas isoform a [�lllit!!§) | 350 | 350 | 100% | 2e-124 | 94.18% | 189 | NP QQ115§715 1 |
| � | 1±11 member(s), 1 organism(s) | human | UBE2L3/KRAS fusion P-rolein [Homo saP-iens) | 351 | 351 | 100% | 5e-123 | 94.18% | 296 | AEA35014.1 |
| � | 1±187 member(s), 87 organism(s) | VVill� | GTPase KRas isoform X3 (Pleurodeles walll] | 346 | 346 | 100% | 3e-122 | 92.59% | 227 | XP Q§2Q84§2l 1 |
| � | 1±110 member(s), 7 organism(s) | bonv: vertebrates | Chain A GTPase KRas N-terminaltv P-rocessed [Homo sa� | 328 | 328 | 89% | 4e-116 | 94.08% | 169 | � |
| � | 1±11 member(s), 1 organism(s) | human | Chain A GTPase KRas N-terminally �[tl2m2...lip�] | 326 | 326 | 89% | 3e-115 | 94.05% | 170 | � |
| � | 1±121 member(s), 3 organism(s) | P-lacentals | Chain A, GTPase KRas [Homo sap� | 319 | 319 | 88% | 2e-112 | 92.77% | 166 | fil!flLI |
| � | 1±133 member(s), 3 organism(s) | animals | Chain A GTPase KRas [Homo saJUfill§J | 317 | 317 | 88% | 2e-111 | 92.17% | 170 | � |
| � | 1±112 member(s), 1 organism(s) | human | Chain B GTPase KRas (Homo sapkfili | 315 | 315 | 88% | le-110 | 92.17% | 189 | � |
| � | 1±14 member(s), 2 organism(s) | jawed vertebrates | Chain A GTPase KRas [�JUfill§J | 300 | 300 | 88% | 9e-105 | 92.17% | 169 | §YRS A |
| � | 1±11 member(s), 1 organism(s) | human | Chain E GTPase KRas isoform X2 (Homo saP.:iens] | 300 | 300 | 88% | le-104 | 92.17% | 191 | 8DGS E |
| � | 1±117 member(s), 16 organism(s) | amniotes | GTPase KRas isoform X2 (e ogona vittice�] | 301 | 301 | 88% | 2e-104 | 92.17% | 209 | XP Q,Q§5§§8§.1 |
| � | 1±1155 member(s), 148 organism(s) | jawed vertebrates | GTPase NRas (Mus musculus) | 300 | 300 | 100% | 2e-104 | 81.58% | 189 | NP 0011555§7.1 |
| � | 1±1305 member(s), 162 organism(s) | !Q2! | GTPase KRas isoform 1 [Mus mu5cvlus) | 298 | 298 | 88% | 7e-104 | 91.57% | 188 | NP 0Q112Q1§2 1 |
| � | 1±150 member(s), 44 organism(s) | jawed vertebrates | kirsten rat sarcoma viral oncogene homolog L homeolog �enoP.us laevis) | 295 | 295 | 88% | 2e-102 | 89.76% | 186 | NP 001081J1§.1 |
| � | 1±14 member(s), 4 organism(s) | jawed vertebrates | GTPase HRas P.:artial (C!!!..e.mydotis mac�i] | 294 | 294 | 88% | 2e-102 | 89.76% | 167 | � |
| � | 1±1156 member(s), 72 organism(s) | root | �P.:rolo-oncog.fill.e. GTPase like 1 (Rattus norveg�) | 293 | 293 | 96% | 7e-102 | 81.87% | 189 | NP 0Q11252,8.1 |
| � | 1±12 member(s), 1 organism(s) | human | Chain R GTPASE HRAS (Homo saP.:igm] | 290 | 290 | 88% | 2e-100 | 88.55% | 185 | 4URU R |
| � | 1±125 member(s), 17 organism(s) | root | Chain A GTPase NRas (Homo sa�) | 289 | 289 | 91% | 2e-100 | 86.05% | 173 | 6ZIZ A |
| � | 1±111 member(s), 9 organism(s) | tetrap. 2!1§ | Chain A GTPase NRas (Homo sap�) | 288 | 288 | 88% | 5e-100 | 88.55% | 166 | fili.!:ILI |
| � | 1±129 member(s), 27 organism(s) | bony: vertebrates | GTPase KRas isoform X4 (Cy.!!2glossus semilaevis) | 279 | 279 | 79% | le-96 | 94.67% | 151 | XP 024211277 1 |
| � | 1±137 member(s), 35 organism(s) | P-lacentals | GTPase HRas isoform 2 [Homo sa�] | 265 | 265 | 79% | 9e-91 | 89.33% | 170 | � |
| � | 1±14 member(s), 4 organism(s) | bony: vertebrates | GTPase KRas isoform X3 (Pseudorca crassidens) | 246 | 246 | 65% | 2e-84 | 95.12% | 123 | XP 0§7552Q47.1 |
| � | 1±11 member(s), 1 organism(s) | human | filp..w.el.n..P.:artial lli.9..m.9...il� | 197 | 197 | 59% | 3e-65 | 92.86% | 112 | � |
| � | 1±16 member(s), 6 organism(s) | tetraP-ods | GTPase KRas isoform 3 (Mus musculus) | 196 | 196 | 53% | 2e-64 | 92.00% | 122 | NP 0Q112Q17J.1 |
| � | 1±1184 member(s}, 147 organism(s) | animals | ras-related P.:rotein RaR.:.1!2..� | 186 | 186 | 86% | 9e-60 | 53.33% | 184 | � |
| � | 1±178 member(s}, 72 organism(s) | jawed vertebrates | ras-related P-rotein RaR•1A (Rattus norveg�J | 186 | 186 | 86% | 1e-59 | 53.94% | 184 | NP 0Q10Q57§5.1 |
| � | 1±11 member(s), 1 organism(s) | human | ��RroteinRaP-:.1.!tlli.9..m.9...ilP.J.eMl | 185 | 185 | 86% | 5e-59 | 53.33% | 199 | � |
| � | f±l5 member(s), 5 organism(s) | tel.@p...rut§ | !:m:1othetical protein GDQ86_008658 (Hymenochirus boett9fil_i] | 181 | 181 | 62% | 2e-58 | 72.03% | 118 | KAG8418Q55.1 |
| � | 1±12 member(s), 2 organism(s) | � | N.:!il_�..Rartial lli.9..m.9...iloJ.e.n.ru | 179 | 179 | 51% | 7e-58 | 89.58% | 96 | AAAJ§5481 |

| | | | | | | | | | |
|---|---|---|---|---|---|---|---|---|---|
| f±l19 member(s), 18 organism(s) | animals | ras-related i:1rotein RaP-:.lll[Eq)lnQps tetfairi] | 178 | 178 | 85% | 8e-57 | 51.83% | 164 | XP 004718161.2 |
| f±l9 member(s), 9 organism(s) | � | RAP1A member of RAS oncogene family transcript variant X3 [!c!l_g_om... | 176 | 176 | 81% | 3e-56 | 53.85% | 157 | KAG3281269.1 |
| f±l5 member(s), 5 organism(s) | .!29.!!� | GTPase KRas isoform 1 lt!2!!!2...ll�] | 174 | 174 | 51% | 5e-56 | 93.81% | 105 | � |
| f±l256 member(s), 206 organism(s) | jawed vertebrates | v-ral simian leukemia viral oncog�g& �fQ.a.!Jig__r... | 175 | 175 | 85% | 4e-55 | 50.62% | 202 | NP 001007322.1 |
| f±l1 member(s), 1 organism(s) | hJm!fil! | ��� ..p...fil1lfil_[�p�) | 171 | 171 | 46% | 1e-54 | 93.10% | 103 | WPW61478.1 |
| 1±117 member(s), 16 organism(s) | tetrap...Q!!l | r.a..s:re!a.1fiP.rotein Ral-A-1ike [Marmota monax) | 172 | 172 | 85% | 4e-54 | 50.00% | 184 | XP 046316600.1 |
| 1±12 member(s), 1 organism(s) | hJm!fil! | RAP1B member of RAS oncog�v..P.artial [�W§fil) | 170 | 170 | 72% | 5e-54 | 56.52% | 139 | KAI2566889.1 |
| f±l12 member(s), 10 organism(s) | bony vertebrates | GTPase NRas isoform 3 (Homo saP-ifills] | 165 | 165 | 51% | 2e-52 | 89.69% | 106 | � |
| 1±111 member(s), 8 organism(s) | jawed vertebrates | RAS related 2 S homeolog (Xeno�] | 167 | 167 | 86% | 2e-52 | 55.21% | 170 | NP 0010857641 |
| f±l44 member(s), 41 organism(s) | bony vertebrates | Ras-related protein Ral-B i:1artial �P-s gistatus) | 167 | 167 | 90% | 4e-52 | 48.26% | 183 | � |
| f±l132 member(s), 124 organism(s) | jawed vertebrates | ras-related i:1rotein R-Ras2 (Rattus norvegicus] | 166 | 166 | 87% | 2e-51 | 53.94% | 204 | NP 0010134521 |
| 1±188 member(s), 88 organism(s) | amniotes | ras-related protein Ral-B (Macaca mulatta) | 166 | 166 | 85% | 3e-51 | 49.08% | 206 | NP 0012447281 |
| 1±111 member(s), 7 organism(s) | jawed vertebrates | ras-related i:1rotein Ral-B isoform X1 [Homo SaP.iens] | 166 | 166 | 90% | 3e-51 | 48.26% | 229 | XP 047301313.1 |
| f±l1 member(s), 1 organism(s) | hJm!fil! | GTPase HRas isoform 5 l!:::!.9.m..2..� | 162 | 162 | 44% | 4e-51 | 88.10% | 104 | � |
| f±l1 member(s), 1 organism(s) | human | Chain A GTP-binding protein Rit1 (t!.Qmo_saruefil) | 162 | 162 | 86% | 2e-50 | 47.56% | 173 | � |
| f±l14 member(s), 12 organism(s) | amniotes | RALAisoform 3...�(�g�y_!es) | 161 | 161 | 79% | 5e-50 | 49.67% | 163 | � |
| f±l5 member(s), 5 organism(s) | amniotes | �proteinRaQ:.1A...i:1artial �f'Y1h!o.!2�] | 159 | 159 | 63% | 5e-50 | 60.00% | 122 | � |
| 1±1160 member(s), 149 organism(s) | � | GTP-binding protein Rit1 isoform 1 (t:12!n2...li�] | 163 | 163 | 86% | 6e-50 | 47.56% | 236 | NP 001243750.1 |
| 1±11 member(s), 1 organism(s) | human | � P.rotein R-Ras2 isoform d l��] | 161 | 161 | 85% | 4e-49 | 54.32% | 250 | NP 001427637.1 |
| 1±130 member(s), 28 organism(s) | placentals | GTP-binding protein Rit2 [Macaca mulatta) | 160 | 160 | 86% | 5e-49 | 47.56% | 217 | NP 001244636.1 |
| 1±189 member(s), 81 organism(s) | � | GTP-binding.rJrotein Rit2 isoform 1 (t:12!n2...li�] | 159 | 159 | 86% | 1e-48 | 47.56% | 217 | � |
| 1±116 member(s), 11 organism(s) | placentals | GTP-binding protein Rit1 isoform X3 (Cebus imitator] | 159 | 159 | 86% | 2e-48 | 47.27% | 237 | XP 037587815.1 |
| 1±11 member(s), 1 organism(s) | human | GTPase KRas isoform 2 (Homo sa�] | 154 | 154 | 42% | 2e-48 | 93.67% | 79 | � |
| f±l8 member(s), 8 organism(s) | amniotes | ras-related g:rotein RaQ-1b isoform 3 (Homo sa�) | 155 | 155 | 86% | 2e-47 | 47.88% | 165 | NP 001238850.1 |
| f±l10 member(s), 10 organism(s) | jawed vertebrates | �groteinM-Ras [filipenser ruthenus] | 155 | 155 | 87% | 2e-47 | 51.81% | 176 | RXM27770.1 |
| f±l137 member(s), 130 organism(s) | placentals | r.a..s:.re!a.1fi:1rotein R-Ras [��) | 155 | 155 | 86% | 5e-47 | 53.05% | 218 | 1'!!"_0Q626Ll |
| 1±12 member(s), 1 organism(s) | hJm!fil! | �.� protein R-Ras [�ru.e.n.s] | 152 | 152 | 86% | 1e-46 | 52.44% | 179 | � |
| f±l2 member(s), 2 organism(s) | � | PREDICTED: ras-related protein Ral-B isoform X1 [�g�... | 154 | 154 | 85% | 3e-46 | 43.48% | 227 | XP 011797028.1 |
| 1±15 member(s), 5 organism(s) | placentals | ras-related P.rotein R-Ras2 isoform X1 lAiY.l.opoda melanoleuca) | 152 | 152 | 85% | 6e-46 | 46.91% | 197 | XP 034501760.1 |
| 1±12 member(s), 2 organism(s) | � | bv:�protein MC885 01§$46 [�gjg.fill.W] | 152 | 152 | 86% | 7e-46 | 45.12% | 203 | KAK24967311 |
| 1±117 member(s), 9 organism(s) | amniotes | ras-related protein M-Ras isoform X2 rDelphinapterus leucasl | 150 | 150 | 87% | 8e-46 | 51.20% | 176 | XP 030617384.1 |
| f±l211 member(s), 193 organism(s) | jawed vertebrates | ras-related i:1rotein M-Ras isoform 1 12recursor (�p.lfill§) | 151 | 151 | 87% | 1e-45 | 50.90% | 208 | NP 0010785181 |
| f±l128 member(s), 117 organism(s) | jawed vertebrates | �P-rotein R-Ras2 isoform c l!:::!.9.m..2..ru.e.n.sJ | 149 | 149 | 75% | 3e-45 | 51.41% | 169 | NP 0011707851 |
| 1±1149 member(s), 140 organism(s) | jawed vertebrates | RAP2C member of RAS oncogene family S homeolog (Xenopus laevis) | 149 | 149 | 86% | 3e-45 | 46.34% | 183 | NP 001080480.1 |
| 1±14 member(s), 4 organism(s) | P.lacentals | RRAS2 isoform 8 (!artial [e.Qngo abelii) | 148 | 148 | 74% | 4e-45 | 51.43% | 156 | PNJ474011 |
| 1±1233 member(s), 200 organism(s) | jawed vertebrates | ras-related wotein RaP-:Z !! [Danio rerio) | 144 | 144 | 86% | 3e-43 | 45.12% | 182 | NP 0010017291 |
| 1±13 member(s), 3 organism(s) | bony vertebrates | hygothetical protein cyQCar 00044341 [C.y:prinus carpiQ.] | 141 | 141 | 58% | 1e-42 | 57.80% | 133 | KTF72573.1 |
| f±l1 member(s), 1 organism(s) | fil!.filfil! | @.S:!e!a.te.g_ wotein Ral-B isoform XG [ti2m.9..si!�l | 143 | 143 | 73% | 2e-42 | 50.00% | 195 | XP 047301321.1 |
| [±) 10 member(s), 10 organism(s) | �g� | RAP18 lll9!ei!L(Aegithal9s caydatus] | 138 | 138 | 58% | 1e-41 | 56.88% | 116 | � |
| f±l36 member(s), 34 organism(s) | !filra� | GTP-binding P.:rotein Rit1 isoform 3 [t:l..9..m.o...l�) | 139 | 139 | 77% | 3e-41 | 44.90% | 183 | NP 001243749.1 |
| f±l147 member(s), 128 organism(s) | jawed vertebrates | @.S:!e!a.te.g_p..!9!el!!...Bi!P.::U_[t!orno_sa� | 139 | 139 | 86% | 4e-41 | 45.12% | 183 | � |
| 1±125 member(s), 25 organism(s) | .!20nv vertebrates | Ras-related protein Rai2:2b...P.:artial (�y.rt:ly�] | 137 | 137 | 77% | 1e-40 | 42.86% | 165 | � |
| f±l2 member(s), 2 organism(s) | great aw | RAS l'ke ruo.19..::2.!J.g�..P.:artial /Homo sai:1iens] | 137 | 137 | 63% | 2e-40 | 52.07% | 171 | KAI2524913.1 |
| 1±16 member(s), 6 organism(s) | amniotes | GTP-binding protein Rit1 isoform X1 (!:ptesicus fuscus] | 133 | 133 | 62% | 2e-39 | 50.85% | 146 | XP 054567432.1 |
| 1±127 member(s), 21 organism(s) | placentals | GTP-binding protein Rit2 isoform 2 [t:!orno_sapiens,] | 131 | 131 | 62% | 2e-38 | 50.00% | 153 | NP 001259006.1 |
| [±) 14 member(s), 14 organism(s) | placentals | @.S:!e!a.te.g_ protein M-Ras isoform X3 (Qnvcruu!Jy�) | 131 | 131 | 74% | 4e-38 | 50.71% | 185 | XP 036048782.1 |
| 1±19 member(s), 9 organism(s) | grimates | GTPase ERas ore.cY fS.QL(Homo sa� | 131 | 131 | 95% | 2e-37 | 42.86% | 233 | � |
| f±l2 member(s), 2 organism(s) | P.:rimates | RAP1B member of RAS onco™1filnlly:..p...fil!@L(t:i.9.m.9..W.lifil!$] | 127 | 127 | 53% | 2e-37 | 56.00% | 101 | KAI2566892.1 |
| f±l1 member(s), 1 organism(s) | hY.!!1fil! | RAS related isoform 3 [�P-iefil:) | 132 | 132 | 63% | 2e-37 | 60.00% | 267 | � |
| f±l9 member(s), 2 organism(s) | ban� | K-Ras �[t!2.fil.o..... s.R!fill§) | 121 | 121 | 32% | 9e-36 | 93.33% | 60 | � |
| f±l5 member(s), 4 organism(s) | animals | !.!.!!.!lar!!li�.�...p. a.!1@! (Adineta ricciae] | 122 | 122 | 53% | 2e-35 | 53.00% | 101 | CAF1625239.1 |
| 1±11 member(s), 1 organism(s) | human | groto-oncogene GTPase partial (Homo sagiens] | 119 | 119 | 31% | 7e-35 | 93.22% | 59 | UBF41788.1 |
| 1±11 member(s), 1 organism(s) | human | GTPase NRas i5oform 1 [Homo sa�) | 115 | 115 | 42% | 4e-33 | 70.00% | 79 | � |
| 1±160 member(s), 57 organism(s) | jawed vertebrates | ras-related and estro�gulated growth inhibitor {Rattus norveg�] | 118 | 118 | 87% | 7e-33 | 38.55% | 199 | NP 0013858581 |
| 1±13 member(s), 2 organism(s) | cellular organisms | RAP1B member of RAS oncogene family...gartial (Homo sa�] | 114 | 114 | 45% | 1e-32 | 58.82% | 85 | KAI2566896.1 |
| f±l76 member(s), 73 organism(s) | !§1.@p_Qd_s; | ras-related and estro9§!!.:!e�growth inhibitor isoform a[�... | 117 | 117 | 87% | 2e-32 | 38.55% | 199 | NP 001157684.1 |
| [±)2 member(s), 2 organism(s) | .!29.!!y� | �ruo.19..::2.!J.gfille.J3,.�l�P�l | 114 | 114 | 51% | 4e-32 | 52.58% | 112 | KAI2524917.1 |
| f±l1 member(s), 1 organism(s) | human | Ras like without CAAX 1 partial (Homo sapiens] | 114 | 114 | 54% | 5e-32 | 50.00% | 122 | KAI2519588.1 |
| f±l1 member(s), 1 organism(s) | human | Chain A RAS-like estroge.!!.:[eg�growth inhibitor [Homo saQ!fil§] | 116 | 116 | 87% | 5e-32 | 37.95% | 196 | 2ATV A |
| f±l1 member(s), 1 organism(s) | hYm.fil! | �l!:::!.9.m..2..� | 113 | 113 | 54% | 8e-32 | 52.88% | 110 | EAW79760.1 |
| f±l11 member(s), 10 organism(s) | jawed vertebrates | ras-related i:1rotein RaP.:-1b isoform 4 (t:i.Qm.Q_sa_�) | 113 | 113 | 86% | 2e-31 | 39.39% | 137 | NP 0012388511 |
| f±l8 member(s), 7 organism(s) | Q_QDY..Yfli_e_h@tg_� | Rcts�fateJt pJ:olein Ral-A [GJIYLa�llata) | 111 | 111 | 50% | 3e-31 | 52.13% | 108 | KFV417991 |

| | | | | | | | | | | |
|---|---|---|---|---|---|---|---|---|---|---|
| ☑ | ⊞1 member(s), 1 organism(s) | human | GTPase KRas isoform 1 [Homo sapiens] | 104 | 104 | 30% | 6e-29 | 87.50% | 78 | UJY53486.1 |
| ☑ | ⊞2 member(s), 1 organism(s) | human | hCG2005194, partial [Homo sapiens] | 108 | 108 | 76% | 7e-29 | 37.24% | 229 | EAW69371.1 |
| ☑ | ⊞4 member(s), 2 organism(s) | bony vertebrates | hypothetical protein M9458_048955, partial [Cirrhinus mrigala] | 102 | 102 | 28% | 3e-28 | 88.68% | 53 | KAL0154692.1 |
| ☑ | ⊞84 member(s), 66 organism(s) | jawed vertebrates | GTP-binding protein Rheb [Danio rerio] | 106 | 106 | 80% | 3e-28 | 34.64% | 184 | NP_957023.1 |
| ☑ | ⊞244 member(s), 178 organism(s) | chordates | ras-related protein Rab-1A [Xenopus tropicalis] | 105 | 105 | 86% | 7e-28 | 34.15% | 204 | NP_001004787.1 |
| ☑ | ⊞16 member(s), 15 organism(s) | bony vertebrates | ras-related protein Rap-2a isoform 2 [Rattus norvegicus] | 102 | 102 | 55% | 8e-28 | 49.04% | 105 | NP_446193.1 |
| ☑ | ⊞2 member(s), 1 organism(s) | human | Chain B, Ras-related protein Rab-1A [Homo sapiens] | 104 | 104 | 86% | 9e-28 | 33.54% | 171 | 4FMB_B |

**Supplementary Table 5: The** cross-reactivity mapping of the studied pan-ras mRNA vaccine carried out with Blastp tools.
